# Simultaneous characterization of cellular RNA structure and function with in-cell SHAPE-Seq

**DOI:** 10.1093/nar/gkv879

**Published:** 2015-09-08

**Authors:** Kyle E. Watters, Timothy R. Abbott, Julius B. Lucks

**Affiliations:** School of Chemical and Biomolecular Engineering, Cornell University, Ithaca, NY 14853, USA

## Abstract

Many non-coding RNAs form structures that interact with cellular machinery to control gene expression. A central goal of molecular and synthetic biology is to uncover design principles linking RNA structure to function to understand and engineer this relationship. Here we report a simple, high-throughput method called in-cell SHAPE-Seq that combines in-cell probing of RNA structure with a measurement of gene expression to simultaneously characterize RNA structure and function in bacterial cells. We use in-cell SHAPE-Seq to study the structure–function relationship of two RNA mechanisms that regulate translation in *Escherichia coli*. We find that nucleotides that participate in RNA–RNA interactions are highly accessible when their binding partner is absent and that changes in RNA structure due to RNA–RNA interactions can be quantitatively correlated to changes in gene expression. We also characterize the cellular structures of three endogenously expressed non-coding RNAs: 5S rRNA, RNase P and the *btuB* riboswitch. Finally, a comparison between in-cell and *in vitro* folded RNA structures revealed remarkable similarities for synthetic RNAs, but significant differences for RNAs that participate in complex cellular interactions. Thus, in-cell SHAPE-Seq represents an easily approachable tool for biologists and engineers to uncover relationships between sequence, structure and function of RNAs in the cell.

## INTRODUCTION

Non-coding RNAs (ncRNAs) have diverse functions, ranging from regulatory roles in transcription, translation and messenger stability in prokaryotes (1,2) to gene silencing, transcript splicing and chromatin remodeling in eukaryotes (3–5). This recognized importance of ncRNAs is accelerating as high-throughput genomics techniques continue to discover new ncRNAs and their roles in globally tuning genome expression (6). Synthetic biologists, in turn, have started to take advantage of this diversity of ncRNA mechanisms to design sophisticated RNA regulators that can precisely control gene expression (7–13). Such widespread use of RNA-based gene regulation in both natural and engineered cellular systems has thus prompted a large effort to understand the relationship between RNA structure and function within the cell (14–16).

This effort has recently accelerated with the advent of high-throughput RNA structure characterization technologies that combine chemical probing with next-generation sequencing (17–24). In one such method, called selective 2′-hydroxyl acylation analyzed by primer extension sequencing (SHAPE-Seq), SHAPE reagents (25) modify the 2′-OH of less-structured RNA nucleotides, which causes reverse transcription (RT) to halt one nucleotide before the modification (26–28). Next-generation sequencing of the resulting cDNA fragments is then used to determine the location and frequency of modifications across each RNA under study. These modification frequencies are then used to estimate a ‘reactivity’ that quantifies the propensity of each nucleotide in an RNA to be modified by the chemical probe (29,30). High reactivities reflect nucleotides that are unstructured, while low reactivities suggest structural constraints such as base pairing, stacking or RNA–ligand interactions (17,22,31).

The use of next-generation sequencing has allowed these methods to be highly multiplexed, which has offered some of the first ‘transcriptome-wide’ glimpses of RNA structure (19–21,23,24). However, the current methods are designed for asking broad questions about cellular RNA structure and are not well suited for extensive structure–function analysis of specific RNA targets. Further, the current monetary costs and computational complexity of analyzing chemical probing data over the entire transcriptome are a significant barrier to overcome for studies requiring many replicates, such as mutational analysis of select RNAs. Yet, simpler methods based on capillary or gel electrophoresis cannot be multiplexed to characterize multiple RNAs at once or remove off-target cDNA products. In addition, other current techniques that use next-generation sequencing often rely on many time-consuming steps for sequencing library preparation (19–21,23,24), such as successive gel purifications that increase turnaround time, cost and skill required to analyze RNA structures inside the cell. Finally, many current techniques focus on characterizing cellular RNA structures, without an explicit measurement of RNA function.

**Figure 1.**
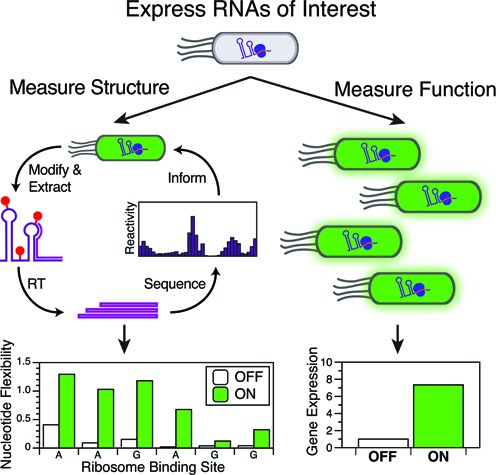
In-cell SHAPE-Seq overview. The in-cell SHAPE-Seq pipeline yields information about RNA structure within the cell by detecting regions of RNA flexibility using an in-cell chemical probe, reverse transcription (RT), next-generation sequencing and bioinformatics. Gene expression measurements yield information about RNA function. Coupling the two measurements provides quantitative information about cellular RNA structure–function relationships.

To address these issues for researchers interested in studying the structure–function relationship of select RNAs in depth, we have developed in-cell SHAPE-Seq. In-cell SHAPE-Seq combines in-cell probing of RNA structure with SHAPE-Seq (22) and a measurement of gene expression through fluorescent reporter assays to characterize RNA regulatory function. By measuring fluorescence and performing the chemical probing experiment on the exact same cell culture, in-cell SHAPE-Seq is able to link changes in cellular RNA structure to changes in gene expression (Figure 1). The use of a new selective polymerase chain reaction (PCR) method during library construction further simplifies the experiment by removing gel-based purification steps. In-cell SHAPE-Seq thus provides nucleotide-resolution structural data for multiple RNAs at a time in a simple experiment that leverages many of the recent technical advances in SHAPE-Seq as well as existing data analysis pipelines (22,29,30).

In this work, we develop and apply in-cell SHAPE-Seq to study the structure–function relationship of two well-characterized RNA regulatory systems in *E. coli*: the synthetic RNA riboregulator translational activator system developed by Isaacs *et al*. (8), and the natural IS10 translational repressor system recently engineered by Mutalik *et al*. (9). To perform these studies, we established a general two-plasmid expression system for studying RNA regulator pairs. Both plasmids contain convenient RT-priming sites that facilitate in-cell SHAPE-Seq measurements, as well as a fluorescent protein reporter on one of the plasmids for coupling to gene expression measurements (Supplementary Figure S1). Using the two-plasmid system, we show how in-cell SHAPE-Seq can be used to derive structural models of cellular RNA folding for these systems. We also show that in-cell SHAPE-Seq data can be used to generate quantitative links between RNA structural changes in the cell and their functional consequences. We then assess the sensitivity of the method by targeting three endogenously expressed RNAs in *E. coli*: 5S rRNA, RNase P and the riboswitch domain of the *btuB* mRNA leader sequence. We show that in-cell SHAPE-Seq reactivity data can be used to corroborate and refine structural models of these functional ncRNAs. Next, we compare data from *in vitro* equilibrium refolding experiments to in-cell SHAPE-Seq reactivities and find intriguing similarities and differences between these folding environments. We end by discussing how in-cell SHAPE-Seq could be immediately applied to uncovering the cellular RNA structure–function relationship of a broad array of RNA regulatory systems.

## MATERIALS AND METHODS

See the Supplementary Methods for a step-by-step protocol of the complete in-cell SHAPE-Seq experiment (Supplementary Figure S2).

### Platform (plasmid) construction

Supplementary Figure S1 describes our standardized platform for expressing sense/antisense regulatory RNA pairs that are not endogenously expressed in *E. coli*. Specific primer designs and detailed cloning procedures to construct the plasmids used in this work, or plasmids for other RNA regulatory systems, can be found in Supplementary Methods. The *cis*-repressed sense RNA (crRNA) and *trans*-activating RNA (taRNA) plasmids were generated by introducing the riboregulator sequences from Isaacs *et al*. (8) into the sense and antisense expression platforms with Gibson Assembly (32). To create the RNA-IN sense plasmids, the original sequence from Mutalik *et al*. (9) of variant S1 was mutated using standard PCR amplification-ligation methods. The antisense RNA-OUT sequences from Mutalik *et al*. (9) were cloned into the antisense plasmid architecture with Gibson Assembly (32). All plasmid sequences are listed in Supplementary Table S2.

### Strains, growth media and RNA expression

Each sense and antisense plasmid was transformed separately, or in combination, into chemically competent *E. coli* TG1 cells. Where indicated, a control antisense plasmid, lacking the antisense RNA sequence but containing a promoter and terminator (Supplementary Figure S1), was used to maintain a consistent cellular load to properly compare fluorescence levels with or without the antisense RNA present. Transformed cells were plated on LB+Agar media with 100 μg/ml carbenicillin, 34 μg/ml chloramphenicol or both and incubated overnight at 37°C. The next day, individual colonies were picked and grown in 1 ml of the appropriate LB+antibiotic in a 2 ml 96-well block (Costar) and grown approximately 17 h overnight at 37°C at 1000 rpm in a VorTemp 56 (Labnet) benchtop shaker. Twenty-four microliters of this overnight culture was then used to subculture into 1.2 ml of LB+antibiotic. *E. coli* TG1 cells without plasmids were prepared in the same way without antibiotic for probing endogenously expressed RNAs. The subculture was grown under the same conditions as the overnight culture for at least 3 h before measuring fluorescence (for cultures containing the sense plasmid with superfolder GFP; SFGFP) and performing structure probing. See Supplementary Methods (steps 17–21) for more details.

### *In vitro* RNA purification

*In vitro* transcription templates for crR12, taR12, RNA-IN S3 C24A A25C and RNA-OUT A3 were prepared using PCR with Taq polymerase (NEB), replacing the *E. coli* promoter with the T7 promoter (TAATACGACTCACTATAGG), followed by ethanol precipitation. The *in vitro* transcription reaction contained 5 μg of template, 40 mM tris-HCl pH 8.0, 20 mM MgCl_2_, 10 mM DTT, 20 mM spermidine, 0.01% Triton X-100, 5 mM NTPs, 60 U of SUPERase-In, 20 μl of purified T7 RNA polymerase, brought to a total of 1 ml with MilliQ H_2_O. The shorter RNAs (taR12 and RNA-OUT A3) were gel purified and passively eluted before ethanol precipitation. The longer RNAs containing the SFGFP sequence (crR12 and RNA-IN S3 C24A A25C) were purified from the transcription reaction using AMPure XP RNA beads according to the manufacturer's instructions.

### RNA modification and fluorescence assay

Fluorescence was measured after at least 3 h of growth by pelleting 150 μl of each subculture and resuspending it in 200 μl PBS buffer with 100 μg/ml kanamycin (to prevent further translation) and assaying for fluorescence (485/520 nm) and optical density (OD_600_), which typically ranged from 0.1 to 0.5. Fluorescence and OD_600_ were first corrected by subtracting values measured for a media blank. Relative fluorescence levels of each culture (except those used for endogenous RNA characterization) were determined by normalizing the fluorescence readout by optical density (FL/OD) and subtracting the FL/OD of the antisense plasmid containing cultures (which did not contain SFGFP) to correct for cell autofluorescence.

For RNA modification with 1-methyl-7-nitroisatoic anhydride (1M7), 500 μl of each 3 h subculture was modified in the 96-well block with 13.3 μl 250 mM 1M7 in DMSO (6.5 mM final) (+) or 13.3 μl DMSO (−) for 3 min before RNA extraction. For the DMS modification, the 1M7 was replaced with 27.75 μl of 13% DMS in ethanol and the DMSO replaced with 27.75 μl ethanol. After 3 min of incubation with DMS, the reaction was quenched with 240 μl 2-mercaptoethanol. See Supplementary Methods (steps 22–32) for a more detailed in-cell modification protocol.

For *in vitro* transcribed RNAs, 10 pmol of RNA total (1 pmol of sense, 9 pmol of antisense for bimolecular experiments) were diluted in 12 μl H_2_O total before denaturing at 95°C for 2 min. The RNAs were than snap-cooled on ice for 1 min before adding 6 μl 3.3X Folding Buffer (333 mM HEPES, 333 mM NaCl, 33 mM MgCl_2_, pH 8.0) and incubated at 37°C for 20 min. The resulting 18 μl were split and added to 1 μl 65 mM 1M7 (6.5 mM final) or 1 μl DMSO and incubated at 37°C for 1 min. The modified *in vitro* RNAs were then ethanol precipitated and dissolved in 10 μl H_2_O before reverse transcription.

### RNA extraction

For in-cell probing experiments, both modified (+) and control (−) samples were pelleted, then resuspended in 100 μl of hot Max Bacterial Enhancement Reagent (Life Technologies) before extraction with TRIzol Reagent (Life Technologies) according to the manufacturer's protocol. Extracted RNA was dissolved in 10 μl of water. See Supplementary Methods (steps 33–39) for more details.

### Reverse transcription

For each RNA sample, 3 μl of 0.5 μM oligonucleotide were added for reverse transcription (RT), except for the *btuB* riboswitch samples to which 3 μl of 50 nM oligonucleotide were added instead. Sense RNAs were extended with an RT primer for SFGFP, antisense RNAs were extended with a primer for the ECK120051404 terminator, and endogenously expressed RNAs were extended with an RNA-specific primer (Supplementary Table S3). For samples containing sense-antisense pairs, 1.5 μl of each primer were mixed together. All RNAs were denatured at 95°C for 2 min, then 65°C for 5 min. After denaturing, each RNA sample was then snap-cooled on ice for 1 min before extension with Superscript III (Life Technologies) at 52°C for 25 min. After RT the RNA was hydrolyzed with 1 μl 10 M NaOH. The solution was then partially neutralized with 5 μl of 1 M hydrochloric acid and ethanol precipitated. See Supplementary Methods (steps 40–51) for more details.

### Adapter ligation

The cDNA from each RT reaction was separately ligated to a ssDNA adapter for Illumina sequencing with CircLigase I ssDNA ligase (Epicentre). Each ligation reaction was incubated at 60°C for 2 h, followed by deactivation at 80°C for 10 min. The ligated cDNA was then ethanol precipitated and dissolved in 20 μl water. Unligated oligonucleotides were removed by purification with 36 μl of Agencourt AMPure XP beads (Beckman Coulter) according to manufacturer's protocol. See Supplementary Methods (steps 52–57) for details.

### Quality control

Each single-stranded cDNA library from a highly expressed RNA was PCR amplified with Phusion polymerase (NEB) for 15 cycles with two forward primers, a selection primer (containing an RNA-specific sequence and part of the forward Illumina adapter) and a longer primer containing all of the forward Illumina adapter, and a fluorescent reverse primer that binds to the reverse Illumina adapter sequence as part of the ligated ssDNA adapter (Supplementary Table S3, Supplementary Figure S3, Supplementary Methods (step 58)). Moderate to weakly expressed RNAs (RNase P and the *btuB* riboswitch) were amplified for 15 cycles without the complete forward Illumina adapter primer first, which was then added for a second set of 15 cycles. Libraries that were derived from cultures that contained both sense and antisense plasmids were amplified separately with one selection primer to visually separate the library qualities of the independent priming locations. The fluorescently tagged amplifications were run on an ABI 3730xl Analyzer with GeneScan 500 LIZ standard (Life Technologies) and checked for the correct full-length product (indicating good RT and PCR) and minimal side product formation. See Supplementary Methods (steps 58–67) for further details.

### dsDNA sequencing library construction

Highly expressed RNA libraries passing quality analysis were PCR amplified with Phusion polymerase (NEB) for 15 cycles using three primers: a forward primer that contained an Illumina adapter, another RNA-specific forward selection primer, and a reverse primer that contained the other Illumina adapter and one of 24 TruSeq indexes (Supplementary Table S3, Supplementary Figure S3). Moderate to weakly expressed RNAs (RNase P and the *btuB* riboswitch) were amplified for 15 cycles without the complete forward Illumina adapter primer first, before it was added for a second set of 15 cycles. Excess primer was removed with ExoI (NEB) before purification with 90 μl of Agencourt AMPure XP beads (Beckman Coulter) according to the manufacturer's protocol. See Supplementary Methods (steps 68–75) for more details.

### Next-generation sequencing

The molarity of the individual libraries was estimated from the lengths and intensity of peaks in the fluorescent quality traces and the concentration of each library measured with the Qubit fluorometer (Life Technologies). All libraries were mixed to have the same final molar concentration and sequenced with the Illumina MiSeq v3 kit or HiSeq 2500 rapid run using 2×35 bp paired end reads. Adapter trimming was turned off during Illumina post-sequencing processing.

### Data analysis

Reactivity spectra were calculated using Spats v0.8.0 and a number of utility scripts to prepare the Illumina output for Spats following previous work (22). Illumina adapter sequences were trimmed from each read using the FASTX toolkit [http://hannonlab.cshl.edu/fastx_toolkit/], then aligned to the target RNA sequences with Bowtie 0.12.8 (33) based on the input RNAs to determine locations of modifications. Spats separates the (+) and (−) channel reads according to the handle sequence, and calculates θ for each nucleotide using statistical corrections for RT drop-off, where θ represents the probability of modification at a particular nucleotide (29,30). Resulting θ values were then normalized to ρ values according to Supplementary Equations 1–3. Reactivities (ρ) greater than 1.25 are considered highly reactive, between 0.5 and 1.25 moderately reactive and less than 0.5 weakly reactive. All data are freely accessible from the RNA Mapping Database (RMDB) (http://rmdb.stanford.edu/repository/) (34) using the IDs in Supplementary Table S4.

### Structure folding predictions

RNA secondary structure predictions were performed using the RNAStructure web server (35). In-cell SHAPE-Seq reactivities (ρ) were used to constrain predictions with the pseudo free energy parameters m (1.1) and b (−0.3) (22) where indicated (Supplementary Equation 4). All computationally predicted folds shown represent the minimum free energy structure.

## RESULTS

### A standardized platform for characterizing RNA structures, interactions and regulatory function in cells

One goal of the in-cell SHAPE-Seq platform is to characterize cellular structural and functional states of regulatory RNAs simultaneously (Figure 1). Often, RNA regulatory function is mediated by structural changes in mRNA targets brought about by specific interactions with other cellular molecules such as ligands (16), small RNAs (sRNAs) (36) or ribosomes (37). We began by first focusing on the natural IS10 and the synthetic riboregulator bacterial sRNA systems that regulate translation in response to RNA–RNA interactions that occur in *trans*. In these systems, translation is controlled by specific RNA structures in the 5′ untranslated region (5′ UTR) of a ‘sense’ target mRNA. Interaction with a *trans*-acting complementary ‘antisense’ RNA sequence causes structural rearrangements to occur, turning downstream gene expression ON in the case of riboregulators, or OFF in the case of the IS10 system.

To characterize RNA regulator function, we began by constructing a standardized platform to separately express both the sense and antisense RNAs of each system in *E. coli* (Supplementary Figure S1) (8,9). In this platform, the sense regulatory RNA sequences were placed downstream of a constitutive promoter and upstream of the superfolder GFP (SFGFP) coding sequence (CDS) (38) on a medium-copy plasmid. The antisense RNAs were placed on a separate high-copy plasmid downstream of the same constitutive promoter (Supplementary Figure S1). Gene expression was then characterized by measuring differences in fluorescence between cultures containing the sense plasmid with the antisense plasmid or an antisense control plasmid (see Materials and Methods).

To characterize cellular RNA structures, we adapted the SHAPE-Seq experiment (17,22,39) to perform the chemical probing step on bacterial cell cultures rather than on *in vitro* pools of purified RNAs, using the ability of certain SHAPE reagents to penetrate living cells (Supplementary Figure S2) (28,40,41). To directly couple RNA structure and function characterization, we added 1-methyl-7-nitroisatoic anhydride (1M7; (+) reaction), or the control solvent dimethyl sulfoxide (DMSO; (−) control), to the same *E. coli* cultures that were assayed for SFGFP fluorescence (Figure 1). While this probing step modifies all RNAs in the cell, our goal was to target the structural measurement to our regulatory RNAs. To do this, we designed highly specific RT primers that would not exhibit non-specific binding to other RNAs in the transcriptome. To target the sense RNA, we chose an RT primer binding site near the 5′ end of the SFGFP CDS from a set of four designed sequences. To target the antisense RNA, we tested a set of efficient transcription terminators (Supplementary Table S1) for specific RT priming capability and found that the synthetic ECK120051404 terminator (42) produced a good quantity of cDNA while remaining highly specific as an RT priming site. Thus, the antisense plasmid contained the ECK120051404 terminator at the 3′ end of the antisense RNA immediately followed by the t500 terminator (42) to improve termination efficiency (Supplementary Figure S1). After chemical probing and RNA extraction, reverse transcription was performed with primers targeting one or both of the priming sites described above, and the resultant cDNAs were input into the standard SHAPE-Seq experimental and data analysis pipelines (Supplementary Figure S2) (17,22,39).

While successful, initial versions of our protocol suffered from an excess of RT primer-sequencing adapter ligation dimers, making it difficult to accumulate enough sequencing reads with our libraries for computational reactivity analysis (29,30). In some cases, the amount of ligation dimer could exceed 90% of the total sequencing reads. To overcome this problem, we developed a simple method of selecting against these unwanted ssDNA dimers by using a mismatch-based selective PCR amplification in place of the normal SHAPE-Seq PCR step (Supplementary Figure S3, Supplementary Methods). By using this mismatch PCR as a filter, we removed the need for laborious gel purification steps typically used in other methods (19–21,23,24), and reduced amplification of potential off-target RT products. With selective PCR, we observed a 10-40-fold reduction in ligation dimer amplification, with a greater reduction observed for cases where higher quantities of cDNA were obtained. Typically, the PCR selection step reduced the amount of ligation dimer to less than 10% of the total sequencing reads. Together, the PCR selection step and the multiplexing capabilities of SHAPE-Seq allowed many in-cell SHAPE-Seq experiments, containing multiple RNAs probed simultaneously, to be sequenced in a single MiSeq run with deep read coverage.

### Characterizing cellular RNA structures of synthetic riboregulators that activate translation

We first used in-cell SHAPE-Seq to examine a synthetic riboregulator system that activates translation in bacteria (8). In the riboregulator system, the 5′ UTR of the sense mRNA is designed to form a hairpin structure that occludes the RBS and blocks translation (Supplementary Figure S4). This *cis*-repressed RNA (crRNA) is thus OFF in the basal state. To activate translation, a *trans*-activating antisense RNA (taRNA) is expressed that base pairs with the crRNA, causing structural rearrangements that expose the RBS and allow translation (ON state).

**Figure 2.**
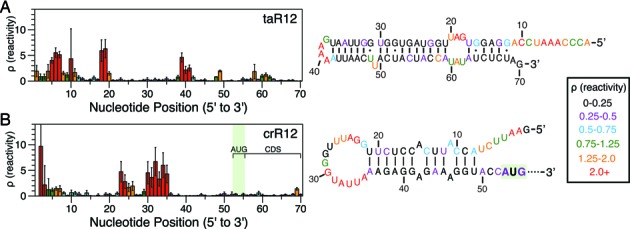
Characterization of the cellular structures of the taR12/crR12 synthetic riboregulator RNA translational activator system. Reactivity maps and constrained secondary structure folds are shown for taR12 (**A**) and crR12 (**B**). Color-coded reactivity spectra represent averages over three independent in-cell SHAPE-Seq experiments, with error bars representing one standard deviation of the reactivities at each position. RNA structures represent minimum free energy structures generated by RNAStructure (35) using average in-cell SHAPE-Seq reactivity data as constraints (see Materials and Methods). Comparisons to the original structural designs from Isaacs *et al*. (8) are shown in Supplementary Figure S5. The crR12 structural model was generated from the first 70 nts of the sequence (55 nt shown). Similarly, the terminators following taR12 were not included in the structural analysis. The start codon (AUG) location is boxed and the coding sequence (CDS) is labeled.

As the riboregulators were first designed *in silico* using computational models of RNA folding (8), we first sought to characterize the cellular structures of crRNAs and taRNAs individually using in-cell SHAPE-Seq. We began our analysis with the taR12/crR12 antisense/sense pair (respectively), which had the highest fold activation of the original riboregulator designs (8). The in-cell SHAPE-Seq reactivity spectra of crR12 and taR12 were largely consistent with the original designed structures, with several notable adjustments (Figure 2, Supplementary Figure S5).

For taR12, designed to be a highly structured hairpin, we observed clusters of high reactivities in all nucleotide positions that were originally expected to be unpaired (Figure 2A, Supplementary Figure S5). In particular, we were able to distinguish the highly unstructured 5′ tail designed to initiate interactions with the crR12 apical loop (8). We could also clearly distinguish the hairpin loop, single nucleotide bulge and inner loop structures within the hairpin. A model of the cellular secondary structure of taR12 generated using in-cell SHAPE-Seq reactivities to constrain computational folding with RNAStructure (35) corroborated these findings, but suggested a larger inner loop structure and an adjustment of the location of the single nucleotide bulge (Supplementary Figure S5).

For crR12, we observed a cluster of high reactivities at the 5′ end and in the middle of the molecule, consistent with the overall hairpin design (Figure 2B). The large cluster of highly reactive positions between nucleotides 22–35 suggested that crR12 contains a larger loop in cells than previously thought, as seen in the reactivity-constrained secondary structure model of the first 70 nts (Figure 2B, Supplementary Figure S5). Notably, this loop structure begins at a designed G-A inner loop which was originally introduced to prevent RNAse cleavage and improve fold activation (8), but may also serve to open the upper portion of the hairpin into a larger loop to improve sense-antisense target recognition. Interestingly, nucleotides 27–29 have lower reactivities than the rest of the loop. These nucleotides are part of a YUNR (Y = pyrimidine, N = nucleotide, R = purine) RNA recognition motif that was included in the riboregulator design to facilitate interactions with the taRNA (8). YUNR motifs are ubiquitous in natural sRNA systems that rely on RNA–RNA interactions to regulate gene expression (11,43), and the lower reactivities could be reflective of stacking interactions between these nucleotides that can occur in this motif (31,44).

When considering the designed structural model, two other regions of crR12 have reactivities lower than expected (Supplementary Figure S5). The first region is the hairpin stem, which is predicted to contain multiple sets of inner loops. Low reactivities in inner loops are not uncommon with SHAPE reactivities (17,31) and could be due to stacking constraints imposed upon the bulged nucleotides by their neighbors or non-canonical base pairing. The second region of low reactivity is from positions 50–70, the majority of which comprise the start of the SFGFP CDS. These low reactivities could be due to several factors, including the binding of cellular proteins, RNA–RNA interactions in the CDS or ribosomes translating at low levels, preventing the chemical probe from accessing this region.

To corroborate our findings, we also examined the taR10/crR10 riboregulator variant, which has a similar overall design and was the second best riboregulator pair in terms of fold activation (8). We repeated the same measurements and found that the in-cell SHAPE-Seq reactivity spectra and constrained structural models were consistent with the taR12/crR12 results (Supplementary Figures S5B and S6).

Additionally, we compared our in-cell SHAPE-Seq results to an equivalent in-cell ‘DMS-Seq-like’ approach (21), where the 1M7 modification was replaced with a DMS modification (Supplementary Figure S7). Overall, we observed very similar reactivities between in-cell SHAPE-Seq and DMS-Seq at comparable nucleotide positions, corroborating our overall in-cell SHAPE-Seq structure probing approach. However, since DMS shows strong preferences for As and Cs (45) the DMS-Seq reactivities show many gaps, especially since the riboregulators are GU-rich. In fact, the DMS-Seq data were unable to uncover the highly reactive loop of crR12 because of its GU-rich nature, further highlighting the benefit of using SHAPE probes to characterize cellular RNA structures.

### Characterizing the cellular RNA interactions and function of synthetic riboregulators that activate translation

**Figure 3.**
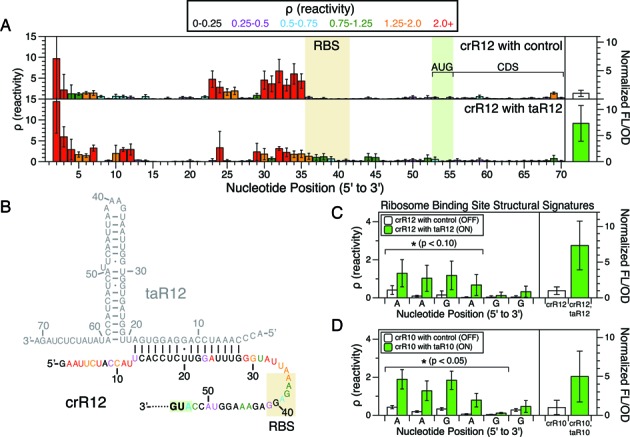
In-cell structure–function characterization of the taR12/crR12 synthetic riboregulator RNA translational activator system. Reactivity maps (A) and a suggested RNA–RNA interaction structure (B) are shown for crR12 of the synthetic riboregulator activator system. (**A**) Color-coded reactivity spectra for crR12 expressed with taR12 or an antisense control plasmid. Reactivities represent averages over three independent in-cell SHAPE-Seq experiments, with error bars representing one standard deviation. Average fluorescence (FL/OD) values (normalized to the crR12 with antisense control plasmid FL/OD value) on the right show a 7.3-fold activation of gene expression when taR12 is expressed, with error bars representing one standard deviation. The ribosome binding site (RBS) (determined in Supplementary Figure S10) and start codon (AUG) locations are boxed. (**B**) Structural model of the taR12/crR12 binding complex derived from the mechanism proposed by Isaacs *et al*. (8) and refined with the average crR12 with taR12 reactivity data in (A). Nucleotides for crR12 are color-coded by reactivity intensity. (**C**) Reactivity and functional data of the RBS region show an increase in RBS reactivity (left) and fluorescence (right) when taR12 is co-expressed with crR12. Nucleotide positions that are significantly different (*P* < 0.10) according to a one-sided Welch's *t*-test are indicated with *. (**D**) RBS reactivity and functional data for the taR10/crR10 variant (see Supplementary Figure S8) Nucleotide positions that are significantly different (*P* < 0.05) are indicated with *.

We next sought to characterize the structural changes of crR12 that occur in the cell when taR12 activates its translation (Figure 3, Supplementary Figure S4). To do this, we performed the full in-cell SHAPE-Seq structure–function measurement in *E. coli* cells expressing both the crR12 sense construct and the taR12 antisense construct. We observed distinct in-cell SHAPE-Seq reactivity changes in several specific regions of crR12 caused by the addition of taR12 that lead to the observed 7.3-fold increase in gene expression (Figure 3A). For example, nucleotides in the 5′ half of the crR12 loop region (nts 22–28) generally decrease in reactivity except for nucleotide 24, which remains high but with large error. The observed reactivity changes in crR12 in the presence of taR12 are consistent with the designed taR12/crR12 structural interaction (Figure 3B) (8). However, nucleotides 4–12, 29 and 30 of crR12 remain or become highly reactive, suggesting that these nucleotides are unbound in the taR12/crR12 complex in the cell. These results from in-cell SHAPE-Seq support a model of the taR12/crR12 complex where a 16 bp duplex forms rather than a 25 bp duplex as originally proposed (8).

Similar features were observed when the structure–function relationship of the taR10/crR10 interaction was characterized with in-cell SHAPE-Seq (Supplementary Figure S8). One difference, however, was a change in the specific nucleotides that were observed to decrease in reactivity as a result of taR10 binding. Overall, more of the 5′ end of crR10 appeared to bind to the taR10 sequence relative to taR12/crR12, though there is a seven nucleotide region from positions 17–23 on crR10 which does not appear to bind as strongly, if at all. One possible explanation for the difference in the interacting structures of these variants is the relative stabilities of the terminal stem loops in taR12 (nt 19–61) and taR10 (nt 22–62). The taR12 hairpin is more stable (ΔG = −20.8 kcal/mol) than the taR10 hairpin (ΔG = −19.6 kcal/mol) as predicted by RNAStructure (35). Therefore, it may be less energetically favorable for taR12 to unwind to the same extent as taR10 when interacting with crR12 or crR10, respectively (Figure 3B, Supplementary Figure S8B). Despite these differences, we observed a similar level of activation of gene expression for each system, suggesting that multiple binding states can achieve the same functional consequence.

Unlike crR12 and crR10, no major reactivity changes were observed for either taR12 or taR10 when expressed with their corresponding crRNA targets (Supplementary Figure S9). Since these RNAs are expressed in excess of their targets, our in-cell SHAPE-Seq experiment is likely capturing a majority of non-interacting taRNA states, as they take up a large portion of the cellular population. We also note that we did not observe significant reactivity changes in either crRNA's CDS when the corresponding taRNA was present.

### Quantitatively linking ribosome binding site reactivity with gene expression

Because the riboregulator mechanism is thought to functionally activate translation in bacteria by removing structural constraints in the crRNA RBS region, we sought to examine how changes in the in-cell SHAPE-Seq reactivities of the RBS region relate to changes in gene expression. However, the AG-rich region between nucleotides 36–46 in crR10 and crR12 has the potential to contain multiple Shine-Dalgarno (SD) sequences. Since the exposure of the RBS turns on gene expression, we hypothesized that the dominant six-nucleotide SD sequence would exhibit the largest reactivity increase. To find this sequence, we summed reactivities over a six nucleotide sliding window for the crR12/crR10 ON and OFF states and looked for the biggest difference between them (Supplementary Figure S10). We found that nucleotides 36–41 showed the largest overall increase, with the most notable increases occurring at nucleotides 36–39 in both crR12 and crR10 (Figure 3C,D, Supplementary Figure S10). These increases correspond to a 6.2-fold and a 4.8-fold change in overall RBS reactivity for the taR12/crR12 and taR10/crR10 systems, respectively, and are linked to 7.3-fold and 5-fold changes in gene expression, respectively (Figure 3C,D).

### Characterizing the cellular RNA structures of the RNA-IN/OUT translational repressor

We next sought to use in-cell SHAPE-Seq to examine a modified version of the natural sRNA translation repression system from the insertion sequence 10 (IS10) transposon (9). In the IS10, or RNA-IN/OUT, system the hairpin loop of an antisense RNA called RNA-OUT initiates interaction with the unstructured 5′ tail of the sense mRNA (RNA-IN) to form a duplex that blocks the RBS and prevents translation in bacteria (Supplementary Figure S11) (46). Recently, six pairs of RNA-IN/OUT variants were designed to be orthogonal, or independently acting, by rationally mutating the sequences that initiate binding (9). We examined two of these pairs with a truncated form of RNA-OUT (first 67 nt) (46,47) using in-cell SHAPE-Seq.

**Figure 4.**
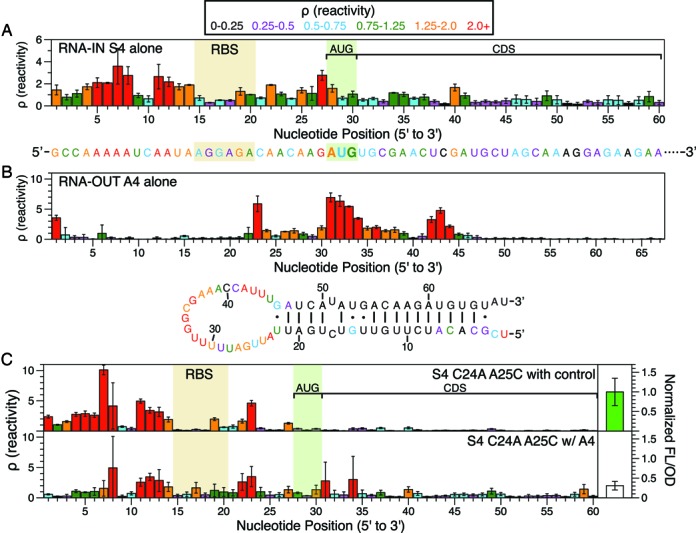
In-cell structure–function characterization of the RNA-IN/OUT translational repressor system. Color-coded reactivity spectra of RNA-IN S4 (A), RNA-OUT A4 (B) and RNA-IN S4 C24A A25C with RNA-OUT A4 or the antisense control plasmid (C) represent averages over three independent in-cell SHAPE-Seq experiments. Error bars represent one standard deviation. All secondary structures are color-coded by reactivity intensity. (**A**) Reactivity spectrum of the first 60 nts of RNA-IN S4 (top), with nucleotides color-coded by reactivity on a single-stranded structural model of this region (bottom). RBS and start codon (AUG) are boxed. (**B**) Reactivity spectrum of RNA-OUT A4 (top), with a minimum free energy structure generated by RNAStructure (35) using in-cell SHAPE-Seq reactivity data as constraints (bottom; see Materials and Methods). The terminators following RNA-OUT A4 were not included in structural analysis. (**C**) Reactivity maps of RNA-IN S4 C24A A25C expressed with RNA-OUT A4 or an antisense control plasmid are on the left. Average fluorescence (FL/OD) values (normalized to the S4 C24A A25C with antisense control plasmid FL/OD value) on the right show 69% repression of gene expression when RNA-OUT A4 is expressed, with error bars representing one standard deviation. The RBS and start codon (AUG) locations are boxed. CDS = coding sequence.

We began by characterizing the in-cell structures of RNA-IN and RNA-OUT individually. Our first observation was that the nucleotides in the RNA-IN S4 5′ UTR were highly reactive and likely unstructured in the cell (Figure 4A). In addition, the RBS region was found to have intermediate reactivities that were similar in magnitude to the riboregulator ON-state RBS reactivities (Figure 3C). For RNA-OUT A4, in-cell SHAPE-Seq reactivities clearly reflected a hairpin structure with a large, highly reactive, loop at the site of RNA-IN recognition (Figure 4B). As with the loops of crR10 and crR12, the secondary structure model of RNA-OUT constrained with in-cell reactivity data showed a much larger loop than previously suggested (47). Similar results were obtained for the S3/A3 RNA-IN/OUT pair analyzed individually (Supplementary Figure S12).

### Characterizing the cellular RNA interactions and function of the IS10 translational repressor

We then characterized how RNA-OUT binding to RNA-IN leads to translation repression by performing the full in-cell SHAPE-Seq structure–function measurement in *E. coli* cells expressing the RNA-IN reporter construct with the RNA-OUT antisense construct. Initially, we performed three replicate experiments with the S4/A4 pair, but observed varying RNA-IN reactivity patterns, despite each replicate exhibiting roughly the same level of translation repression (∼85%) (Supplementary Figure S13). A closer analysis of the raw SHAPE-Seq (+) and (−) channel fragment distributions revealed large spikes at position 26 in both channels, suggesting that RNA-IN S4 was being cleaved between positions 25 and 26 when in complex with RNA-OUT A4. To further confirm this effect was due to cognate RNA–RNA interactions, we examined orthogonal pairs of RNA-IN/RNA-OUT (i.e. pairs S4/A3 and S3/A4) and found no spikes at position 26 or major changes in reactivity compared to the individually measured RNAs (Supplementary Figures S14 and S15).

Previous work showed that the wild-type RNA-IN/RNA-OUT duplex is targeted by RNAse III between nucleotides 15–16 of RNA-IN and 22–23 of RNA-OUT for degradation in the cell (48). However, we did not observe spikes at these positions due to mutations introduced at positions 16 and 17 of RNA-IN that form bulges in the RNA-IN/RNA-OUT complex and abolish RNAse III cleavage (9). Given the propensity for the wild-type system to be cleaved by RNAse III, we hypothesized that a secondary RNAse III site was present between nucleotides 25 and 26 that gave rise to the observed spikes in the fragment distributions from the cognate complexes. To test this hypothesis, we made two different mutations (C24A and A25C) to RNA-IN S4 to prevent RNAse III cleavage (Supplementary Figure S16) and tested them using in-cell SHAPE-Seq. We observed that both mutants were still functional and neither generated a fragment spike at position 26 when expressed with RNA-OUT A4, indicating that cleavage was abolished by these changes. We also tested a double mutant version that functioned similarly (Supplementary Figure S17).

To characterize the cellular RNA–RNA interactions that lead to translation repression, we performed replicate in-cell SHAPE-Seq experiments with the RNA-IN S4 C24A A25C double mutant and RNA-OUT A4 (Figure 4C). Several notable features are apparent when comparing the RNA-IN reactivity spectra with and without RNA-OUT. First, there is a drop in the reactivity spectrum for the first seven nucleotides of RNA-IN where RNA-OUT is predicted to initiate binding, similar to what we observed for the riboregulators (Figure 3A), corresponding to a 69% decrease in measured fluorescence. Second, we observed reactivity increases at positions 16 and 17 in the RBS of RNA-IN, which are predicted to form a bulge when in complex with RNA-OUT (Supplementary Figure S16). We also observed slight increases in reactivity across the CDS and start codon when translation is repressed (Figure 4C). Interestingly, we did not observe a drop in reactivity in the RBS in the presence of RNA-OUT as we might expect, but rather a few nucleotides that increase (Supplementary Figure S18). It could be the case that the interaction between the 5′ end of RNA-IN with the loop of RNA-OUT brings the two RNAs close enough together to hinder ribosome access without directly binding the RBS. We also note that the consistently high reactivities in nucleotides 11–13 are unexpected, suggesting that the duplex between RNA-IN and RNA-OUT may not be as extensive in the cell as originally thought.

Finally, we examined reactivity changes from the perspective of the antisense RNA-OUT RNAs (Supplementary Figure S19). As expected, there are no major differences in the reactivity map of RNA-OUT A4 when the orthogonal RNA-IN S3 is present. However, unlike in the riboregulator system, we did observe subtle reactivity changes in RNA-OUT A4 in the presence of RNA-IN S4 C24A A25C, despite the copy number difference.

### Targeting endogenous RNAs in *E. coli*

**Figure 5.**
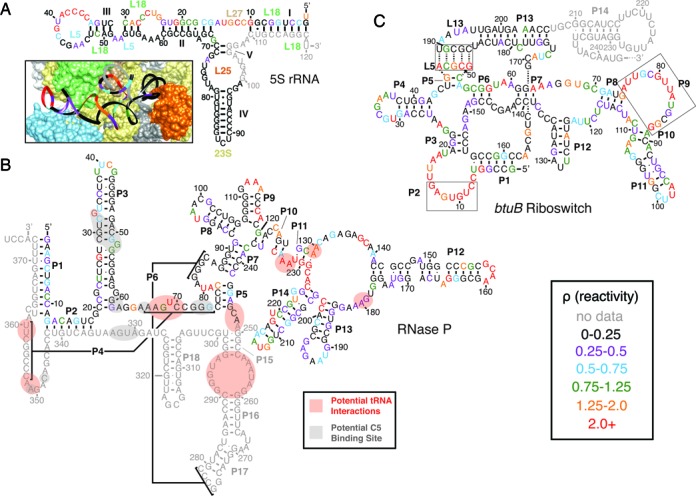
Structural characterization of three endogenously expressed RNAs in *E. coli* with in-cell SHAPE-Seq. RNA secondary structures are color-coded by average in-cell SHAPE-Seq reactivity intensity according to the key in the lower right. Nucleotides not included in the reactivity calculation are marked in gray. Bar charts depicting the average reactivities of each RNA can be found in Supplementary Figure S21. (**A**) 5S rRNA. Reactivities overlaid on the accepted secondary structure (52) and an atomic resolution model of the ribosome derived from cryo-EM data fit with molecular dynamics simulations (inset; from PDB 4V69) (50). Individual ribosomal proteins (L5, L18, L25, L27) and the 23S rRNA are labeled on the secondary structure near their approximate locations and are color-coded to match the three dimensional model. Helices are numbered I-V. (**B**) RNase P. Reactivities overlaid on the accepted secondary structure derived from comparative sequence analysis (54). Potential interactions with tRNAs are highlighted with pink shading according to the crystal structure of the related A-type *T. maritima* RNase P in complex with tRNA^Phe^ (56). Similarly, the expected interactions with the C5 protein measured from hydroxyl-radical mediated cleavage interactions (55) are indicated with gray shading. Helices P1-P18 are labeled. (**C**) *btuB* riboswitch domain. Reactivities overlaid on secondary structure model (60,61). Boxes indicate regions where the structural model was refined according to the high reactivities observed by opening base pairs in those regions. Dashed lines indicate a predicted pseudoknot between L5 and L13 according to the model, though high reactivities are observed in L5 in the cell.

To further test the capabilities of in-cell SHAPE-Seq, we targeted three endogenously expressed functional RNAs that are present at varying levels in *E. coli* cells: 5S rRNA, RNase P and the *btuB* mRNA riboswitch domain (Figure 5). 1M7 probing of *E. coli* cell cultures was performed as before, except that sequence specific RT primers were used for each endogenous target. For the highly abundant 5S rRNA the experiment was straightforward, as the level of cDNA obtained was similar to the synthetic RNAs expressed from plasmids. For the less abundant RNase P and *btuB* riboswitch RNAs, however, it was necessary to modify the PCR steps to prevent the amplification of unwanted side products that began accruing when the amount of correct cDNA product was low and more than 15 cycles of PCR were used. We determined that the side products were due to the Illumina forward primer (primer I in Supplementary Table S3). To remedy this, we first amplified the ssDNA libraries without this primer for 15 cycles to amplify the target of interest, then added primer I for another 15 cycles to build the rest of the adapter required for sequencing (see Materials and Methods). We confirmed the additional cycles did not alter the resulting reactivities (Supplementary Figure S20).

We first examined the highly abundant 5S rRNA (49). As seen in Figure 5A, we observed strong agreement between in-cell SHAPE-Seq reactivities (Supplementary Figure S21A) and the accepted secondary structure and an atomic resolution model of 5S within the ribosome (50–52). Reactivities for the 5S rRNA appeared high in loop regions as expected, except when in close proximity to, or bound locally by, ribosomal proteins such as L5, L18 and L25 (see Supplementary Movie S1). Positions 70–99 were very low in reactivity, which is consistent with helices IV and V being threaded into the interior of the ribosome and the inner loop between helices IV and V interacting with protein L25. We did notice one discrepancy in which nucleotides 28–30 are observed to be highly reactive even though they are predicted to be paired with nucleotides 54–56. In this region however, the 5S rRNA appears to be distorted with nucleotides 54–56 positioned near the L5 protein (see Supplementary Movie S1).

We then characterized the reactivities of RNase P, a ribozyme that complexes with a protein cofactor (C5) to cleave the 5′-leader from precursor tRNAs (pre-tRNAs) (53). The RNase P RNA (RPR) consists of two domains: a catalytic and a specificity domain. We largely focused our analysis on the latter. We found strong agreement between the measured in-cell SHAPE-Seq reactivities (Supplementary Figure S21B) and the secondary structure of the *E. coli* RPR derived from comparative sequence analysis (54) (Figure 5B). Specifically, there is concurrence between highly reactive positions and nucleotides expected to be unpaired in the secondary structure. Because the binding sites for the C5 protein are largely in structured regions or regions not probed (for instance, helices P3 and P4) (55–57), it is difficult to attribute low reactivities that arise in these regions specifically to protein-RPR interactions.

Also shown in Figure 5B are potential sites for tRNA recognition based on the crystal structure of the related A-type *Thermotoga maritima* RNAse P in complex with tRNA^Phe^ (56). Interestingly, we observe several features in this region suggesting that our experiments likely captured the substrate-bound form of RNase P *in vivo*. First, we observe very low reactivity at position A180, which is expected to stack directly with the nucleotides in the T-loop of the pre-tRNA to enable substrate recognition (56,58). Second, we observe low reactivity at position A248, which stabilizes the RPR-pre-tRNA complex through stacking interactions with the pre-tRNA (56). Finally, we observe very high reactivity at position U69, a universally-conserved nucleotide, which is unstacked from pseudoknot P4 to coordinate one of the two divalent metal ions needed for pre-tRNA cleavage (56). Collectively, these observations suggest that our probing experiments have captured the substrate-bound form of RNase P *in vivo*, which could be expected given the large number of pre-tRNAs that need to be processed by RNase P, a low copy-number enzyme (59).

To further test the sensitivity of in-cell SHAPE-Seq, we targeted the endogenously expressed riboswitch domain of the *btuB* mRNA, which regulates the translation of the cobalamin transport protein BtuB in bacteria by sequestering its RBS when adenosylcobalamin (AdoCbl) is present (60). In-cell SHAPE-Seq reactivities (Supplementary Figure S21C) were largely consistent with a secondary structure model of the *btuB* riboswitch derived from comparative sequence analysis and structural probing (60) (Figure 5C). We did, however, observe high reactivities in several areas that are predicted to be paired according to the model. Specifically, the nucleotides comprising the P2 and P9 helices were observed to be highly reactive, indicating that they are unstructured in the cell. In the case of P9, this would suggest this region is disordered as was observed in the crystal structure of the *T. tengcongensis* AdoCbl riboswitch (*Tte*AdoCbl) (61). Most interesting are the high reactivities observed in the loop of P5 (L5), which is expected to form a kissing-loop (KL) interaction with the loop of P13 (L13). Recently, it was shown that this KL interaction is a critical regulatory feature of AdoCbl riboswitches, and crystal structures of the *Tte*AdoCbl riboswitch showed that bound AdoCbl interacts with the groove of the KL in a structure-specific way that promotes its formation (61). While the in-cell SHAPE-Seq reactivities of L13 were observed to be low, the very high reactivities in L5 suggest that there is a significant population of *btuB* riboswitches that are unbound by AdoCbl, or that the KL interaction is flexible enough to allow the riboswitch to significantly sample the non-KL configuration. Additional in-cell SHAPE-Seq analysis on functionally variant mutants of this system would help shed further light on the cellular structural state of this riboswitch.

Overall, these results indicate that in-cell SHAPE-Seq can be used to obtain nucleotide-resolution reactivity maps for endogenous transcripts directly in *E. coli* cells. Our range of examples demonstrate that these reactivity spectra can be used to corroborate existing models of RNA folding and interactions, as well as suggest refinements to our understanding of RNA systems that are less well studied. We thus anticipate in-cell SHAPE-Seq to be useful for the study of a broad array of endogenous RNAs.

### Comparing *in vitro* and in-cell SHAPE-Seq reactivities

Our ability to characterize cellular RNA structures with in-cell SHAPE-Seq gave us an opportunity to compare our results with reactivities generated with *in vitro* SHAPE-Seq experiments (22) to study how the cellular environment affects RNA structure. To begin this study, we performed equilibrium refolding SHAPE-Seq v2.0 experiments on the riboregulators and the RNA-IN/OUT systems following our previously published protocol using the same RT primers as the in-cell experiment (22). Interestingly, we found remarkable agreement between in-cell and *in vitro* refolded SHAPE-Seq reactivities for the riboregulators (Supplementary Figure S22) and the RNA-IN/OUT systems (Supplementary Figure S23). In many cases, the trends in reactivities across the molecules were consistent, with quantitative differences at isolated positions. The biggest deviations were seen when we examined the RNA-IN/OUT complex, which showed significantly lower in-cell reactivities in the region surrounding the RBS of RNA-IN (Supplementary Figure S23B). Overall, the similarity between the in-cell and *in vitro* refolded SHAPE-Seq reactivities suggests that for these regulatory RNAs the complex cellular environment does not play a significant role in altering structures from their equilibrium states.

Next, we performed similar in-cell vs. *in vitro* SHAPE-Seq experimental comparisons for 5S rRNA, which is routinely used as a benchmark for *in vitro* RNA folding (Supplementary Figure S24) (22). In contrast to the above results, we observed dramatic differences in reactivities between these two conditions. In particular, large reactivity differences were observed at positions 35–54 near the site of L5 interactions (Figure 5A) (51). In addition, almost all peaks that are highly reactive downstream of position 54 *in vitro* are near zero in-cell. All of these changes visible in the in-cell reactivity spectra reflect a structural state of the 5S rRNA docked into the ribosome (Figure 5A, inset). It is thus clear that the cellular environment can significantly alter the folding of certain RNAs.

## DISCUSSION

In this work, we established in-cell SHAPE-Seq, which was designed to characterize the cellular RNA structure and function of a set of RNAs in a single experiment. With the coupling of structure and function measurements, we showed how we can use in-cell SHAPE-Seq to directly correlate changes in cellular RNA structure with changes in cellular function in bacteria. The development of in-cell SHAPE-Seq required a number of technical modifications of *in vitro* SHAPE-Seq, including the use of highly specific reverse transcription priming sites to target select RNAs, PCR selection against ligation dimers and off-target cDNAs (Supplementary Figure S3), and a flexible platform for rapid functional characterization of RNA regulators in *E. coli*. All of these improvements enabled deep read coverage for many in-cell SHAPE-Seq experiments in a single MiSeq run with less effort than current in-cell next-generation sequencing-based techniques (19–21,23,24), partly because we removed the need for gel purification in the library construction process. We used these improvements to report some of the first detailed replicate in-cell RNA structure chemical probing data, which we anticipate will be important to the field for understanding the variability of cellular RNA structural states.

We demonstrated the capabilities of our in-cell SHAPE-Seq technique for studying RNA structure–function by applying it to two different RNA regulatory systems: the synthetic riboregulator translational activator (8) and the RNA-IN/OUT translational repressor (9). Each system consists of a pair of RNAs – a sense 5′ UTR containing the RBS of a downstream gene and an antisense RNA that targets the sense RNA to cause structural rearrangements near the RBS, leading to changes in gene expression. In general, we observed that the in-cell SHAPE-Seq reactivity spectra of the isolated sense and antisense RNAs agreed well with the structural models for both systems. For example, the reactivity patterns clearly reflect the hairpin nature of the antisense taRNAs (Figure 2A, Supplementary Figure S6A), the sense crRNAs (Figure 2B, Supplementary Figure S6B) and RNA-OUT (Figure 4B, Supplementary Figure S12B). Interestingly, the loops of the crRNA and RNA-OUT hairpins exhibited a larger span of high reactivities than expected. By constraining computational folding algorithms with in-cell SHAPE-Seq data, we generated structural models that suggested these loops are more unstructured in bacterial cells than originally predicted (Figures 2B and 4B) (8,9). The extensive clusters of high reactivities in these RNAs may actually be an important feature for RNA–RNA recognition, as both loops are involved in initiating interactions between the sense and antisense RNAs of their respective systems.

We also observed low reactivities in the CDS of both sense RNAs in all conditions tested. However, there are many potential causes for low SHAPE reactivity values in these regions including: structures within the CDS, cellular protein binding or the presence of translating ribosomes. In contrast, the transcriptome-wide structural analysis performed by Rouskin *et al*. indicated that translating ribosomes were not associated with lower reactivities, although their experiment was performed in *S. cerevisiae*, not *E. coli* (21). Ding *et al*. alternatively observed a three-nucleotide periodic reactivity pattern in coding sequences across the *Arabidopsis* transcriptome. Although we did not observe any such periodic pattern, our experiments were performed in a different organism and we focused on specific RNAs rather than averaging reactivity signatures over large windows (19).

When antisense RNAs were co-expressed with the matching sense RNAs, we found substantial reactivity changes that could be directly linked to functional changes in gene expression. In the riboregulator system we observed reactivity increases in the RBS that correlated with an increase in SFGFP expression (Figure 3C,D). We also detected other changes in the crRNA reactivity map that led us to refine the model of taRNA/crRNA interactions (Figure 3B). In the RNA-IN/OUT system, this analysis was initially complicated by our discovery of a double-stranded RNAse cleavage site in RNA-IN based on analysis of the raw in-cell SHAPE-Seq fragment alignments (Supplementary Figure S13). Thus, we mutated RNA-IN to remove the cleavage site and performed the in-cell SHAPE-Seq experiment on the cleavage-resistant double mutant and found it exhibited a regular fragment distribution (Supplementary Figure S17). Structurally, we observed reactivity decreases that corresponded to RNA-IN/OUT complex formation, as well as reactivity increases that implied the complex is less structured in parts than the proposed mechanism would suggest (Figure 4C) (9). We note that changes in RBS reactivity between the two functional states of the RNA-IN/OUT system were not as clear as those for the riboregulators (Figure 4C, Supplementary Figure S18). However, we did detect an interaction at the 5′ end of RNA-IN, which could serve to bring RNA-OUT close enough to hinder ribosome access without directly binding the RBS. All together, our in-cell SHAPE-Seq reactivity data speak to the fact that RNA structures typically exist in an ensemble and suggests that different RNA structural states can give rise to similar functional outputs.

We also demonstrated that in-cell SHAPE-Seq could be used to characterize endogenous bacterial RNAs expressed at a range of levels. In particular, we showed that in-cell SHAPE-Seq reactivities recapitulated many of the structural features and interactions of two well-studied RNAs that interact with known proteins: 5S rRNA and RNase P (Figure 5A,B). An additional study of the *btuB* riboswitch suggested interesting refinements to the covariation/*in vitro* probing-based structural model that could reflect differences in folding due to the cellular environment (Figure 5C). To obtain these reactivity spectra, we needed to modify the PCR steps of our library preparation strategy in order to improve selectivity and prevent undesired DNA from dominating the libraries. With minor modifications, we were able to obtain a robust in-cell SHAPE-Seq method that should be applicable to studying a broad range of endogenously expressed RNAs. This could be a particular advantage of the targeted in-cell SHAPE-Seq approach, especially for lowly expressed RNAs, since transcriptome-wide approaches do not capture low abundance transcripts as well, as they inherently distribute reads across a large number of targets. We note that both targeted and transcriptome-wide approaches have distinct advantages and can be viewed as complementary methods to study cellular RNA structure–function principles.

Finally, this work enabled us to study how the complex cellular environment can affect RNA folding. This was most clear in a comparison between SHAPE-Seq reactivities from *in vitro* equilibrium and in-cell experiments on 5S rRNA (Supplementary Figure S24), where a large number of changes were observed that matched well with the known interactions of 5S rRNA within the ribosome (Figure 5A, Supplementary Movie S1). Thus, we found that the cellular environment can significantly affect RNA folding, even for highly expressed RNAs. A similar comparison for the synthetic riboregulator and RNA-IN/OUT systems showed the opposite, with strong agreement observed between *in vitro* and in-cell reactivities (Supplementary Figures S22 and S23). While these systems are designed to interact with ribosomes in the cell, these interactions may be too fleeting, or not present at high enough abundance, to be detected within the population of RNAs probed in these experiments, as was the case with the antisense RNAs for these systems (Supplementary Figures S9 and S19). Consistent with our results, while this manuscript was under review, a complementary in-cell SHAPE probing technique called icSHAPE was used to show that the agreement between *in vitro* and in-cell RNA folds was closer than previously expected, especially near translation initiation regions (23). This intriguing agreement could reflect the robustness of the biophysics of RNA folding to environmental perturbations and warrants further study.

We anticipate in-cell SHAPE-Seq to be applicable to studying cellular RNA structure–function relationships within a broad array of mechanistic and cellular contexts, including other organisms beyond *E. coli* such as *S. cerevisiae, M. musculus* or *A. thaliana* (19,20,23,24). While we focused on regulatory systems containing two RNAs and several endogenously expressed RNAs, the inherent multiplexing and accuracy of SHAPE-Seq (17,22) allows many RNAs to be measured at once, enabling the study of larger mixed populations of cellular RNAs. In its current form, in-cell SHAPE-Seq could be immediately applied to study a host of RNA regulators including ligand-sensing riboswitches, ribozymes, bacterial small RNAs and other RNAs that affect aspects of gene expression (7). In addition, performing in-cell SHAPE-Seq experiments alongside *in vitro* SHAPE-Seq experiments offers a way to reveal interactions and structural changes that may be present in the cellular environment as we demonstrated with 5S rRNA. Further, we have provided a detailed step-by-step protocol in the Supplementary Methods to facilitate the application of in-cell SHAPE-Seq to other systems, including RT primer design guidelines. We expect that in-cell SHAPE-Seq will be an easily approachable tool for biologists and engineers to uncover relationships between the sequence, structure and function of RNAs in the cell.

## Supplementary Material

SUPPLEMENTARY DATA
