# Supplementary material for: Simultaneous characterization of cellular RNA structure and function with in-cell SHAPE-Seq
Source: Nucleic Acids Res. 2015 Sep 8;44(2):e12. doi: 10.1093/nar/gkv879 (PMC4737173; doi:10.1093/nar/gkv879)
Supplement: SUPPLEMENTARY DATA [file supp_44_2_e12__index.html]

Simultaneous characterization of cellular RNA structure and function with in-cell SHAPE-Seq — SUPPLEMENTARY DATA 

# Simultaneous characterization of cellular RNA structure and function with in-cell SHAPE-Seq

## SUPPLEMENTARY DATA

- SUPPLEMENTARY DATA
- SUPPLEMENTARY DATA
