# Supplementary material for: Simultaneous characterization of cellular RNA structure and function with in-cell SHAPE-Seq
Source: Nucleic Acids Res. 2015 Sep 8;44(2):e12. doi: 10.1093/nar/gkv879 (PMC4737173; doi:10.1093/nar/gkv879)
Supplement: SUPPLEMENTARY DATA [file supp_gkv879_Watters_Supplementary_Information_gkv879_FINAL.pdf]

Supplementary Information for:

Simultaneous characterization of cellular RNA structure and function with in-cell SHAPE-Seq

Kyle E. Watters[1], Timothy R. Abbott[1], and Julius B. Lucks[1]*

1 – School of Chemical and Biomolecular Engineering, Cornell University, 120 Olin Hall, Ithaca NY 14853

*To whom correspondence should be addressed. Tel: 607-255-3601, Fax: 607-255-9166, Email: jblucks@cornell.edu

Supplementary Item	Name	Page
Supplementary Figure 1	Standardized platform for expressing sense/antisense regulatory RNA pairs in *E. coli*	3
Supplementary Figure 2	In-cell SHAPE-Seq structural characterization overview	4
Supplementary Table 1	List of terminators screened for building the antisense platform	5
Supplementary Figure 3	Selective PCR amplification strategy for cDNA libraries	6
Supplementary Figure 4	Mechanism of the synthetic translation-activating riboregulator system	7
Supplementary Figure 5	Structural analysis of the synthetic riboregulator translational activation system using in-cell SHAPE-Seq data	8
Supplementary Figure 6	Characterization of the cellular structures of the taR10/crR10 synthetic riboregulator RNA translational activator system	9
Supplementary Figure 7	Characterization of the cellular structures of the taR12/crR12 synthetic riboregulator RNA translational activator system using in-cell dimethyl sulfate (DMS) probing	10
Supplementary Figure 8	Structure-function characterization of the taR10/crR10 synthetic riboregulator RNA translational activator system	11
Supplementary Figure 9	In-cell SHAPE-Seq reactivities for the trans-activating RNA variants	12
Supplementary Figure 10	Determining the dominant RBS sequence in crRNAs using in-cell SHAPE-Seq reactivities	13
Supplementary Figure 11	Mechanism of the translation-repressing RNA-IN/OUT system	14
Supplementary Figure 12	Characterization of the cellular structures of the S3/A3 RNA-IN/OUT translational repressor system	15
Supplementary Figure 13	RNA-IN/OUT S4/A4 interaction complexes appear to be cleaved by a double-stranded RNase in the cell	16
Supplementary Figure 14	Structure-function analysis of the S4/A3 interaction from the RNA-IN/OUT translational repressor	17
Supplementary Figure 15	Structure-function analysis of the S3/A4 interaction from the RNA-IN/OUT translational repressor	18
Supplementary Figure 16	RNA-IN mutations to resist RNase cleavage between nucleotides 25 and 26	19
Supplementary Figure 17	RNA-IN S4 mutations resist RNase cleavage and maintain functionality	20
Supplementary Figure 18	Ribosome binding site analysis of RNA-IN S4 mutant C24A A25C	21
Supplementary Figure 19	In-cell SHAPE-Seq reactivities for the antisense RNA-OUT A4 with RNA-IN variants	22
Supplementary Figure 20	Increasing PCR selection does not affect reactivity calculation	23
Supplementary Figure 21	Reactivity maps for endogenous RNA targets	24

Supplementary Figure 22	In-cell vs. equilibrium *in vitro* refolding reactivity maps for riboregulators	25
Supplementary Figure 23	In-cell vs. equilibrium *in vitro* refolding reactivity maps for RNA-IN/OUT	26
Supplementary Figure 24	In-cell vs. equilibrium *in vitro* refolding reactivity maps of 5S rRNA from *E. coli*	27
Supplementary Table 2	RNA sequences and plasmids used in this study	28-30
Supplementary Table 3	Oligonucleotides used in this study	31-32
Supplementary Equations 1-4	Normalization of reactivities and constrained structure prediction	33
Supplementary Table 4	RMDB data deposition table	34
Supplementary Methods	Detailed in-cell SHAPE-Seq protocol	35-46
References		47
Supplementary Movie 1	Atomic resolution model of 5S rRNA in the ribosome (PDB 4V69) colored by in-cell SHAPE-Seq reactivities from Supplementary Figure S21a.	

Supplementary Figure 1. Standardized platform for expressing sense/antisense regulatory RNA pairs in *E. coli*. Sense RNAs that control the translation of a downstream superfolder GFP (SFGFP) sequence (1) are expressed in *E. coli* using a constitutive promoter (σ^{70}) as part of the Sense Platform. The Antisense Platform similarly expresses an antisense RNA that can target the sense RNA. The origins of replications were selected such that the antisense is always in molar excess of the sense to facilitate RNA binding. Specific sequences of RNA regulators are found in Supplementary Table S2. Note that the sense platform shown below is for the riboregulators crR10 and crR12. The TrrnB operon fragment of the sense platform is replaced with a double terminator for the RNA-IN/OUT system. The Antisense Platform contains the ECK120051404 and t500 intrinsic terminators (2). The control antisense plasmid lacks the antisense sRNA sequence, but is otherwise the same as the Antisense Platform vector. A selection of the plasmids used in this study was deposited in the addgene database and can be found by searching for this paper. Other plasmids can be provided upon request.

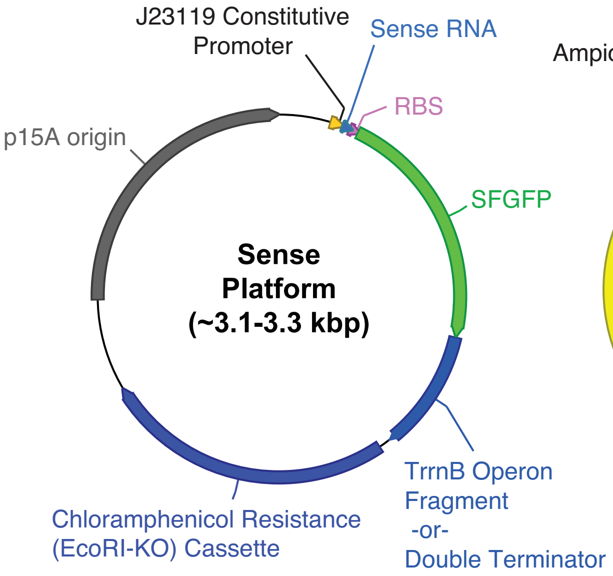

Supplementary Figure 2. In-cell SHAPE-Seq structural characterization overview. To perform in-cell SHAPE-Seq, cells are grown (potentially with a plasmid, or combination of plasmids, containing the RNA(s) of interest) then split for fluorescence measurement and SHAPE probing (Figure 1). Cultures for SHAPE probing are subjected to modification with 1M7 (+) or a DMSO control (-). Subsequent RNA extraction, reverse transcription (halting at modifications), and PCR prepare the cDNA fragments for next-generation sequencing. Bioinformatic analysis of sequenced reads with Spats (http://spats.sourceforge.net/) generates reactivity maps representing the cellular flexibility of each nucleotide in an RNA (3, 4). $tRNA^{phe}$ from *E. coli* is shown as a hypothetical example.

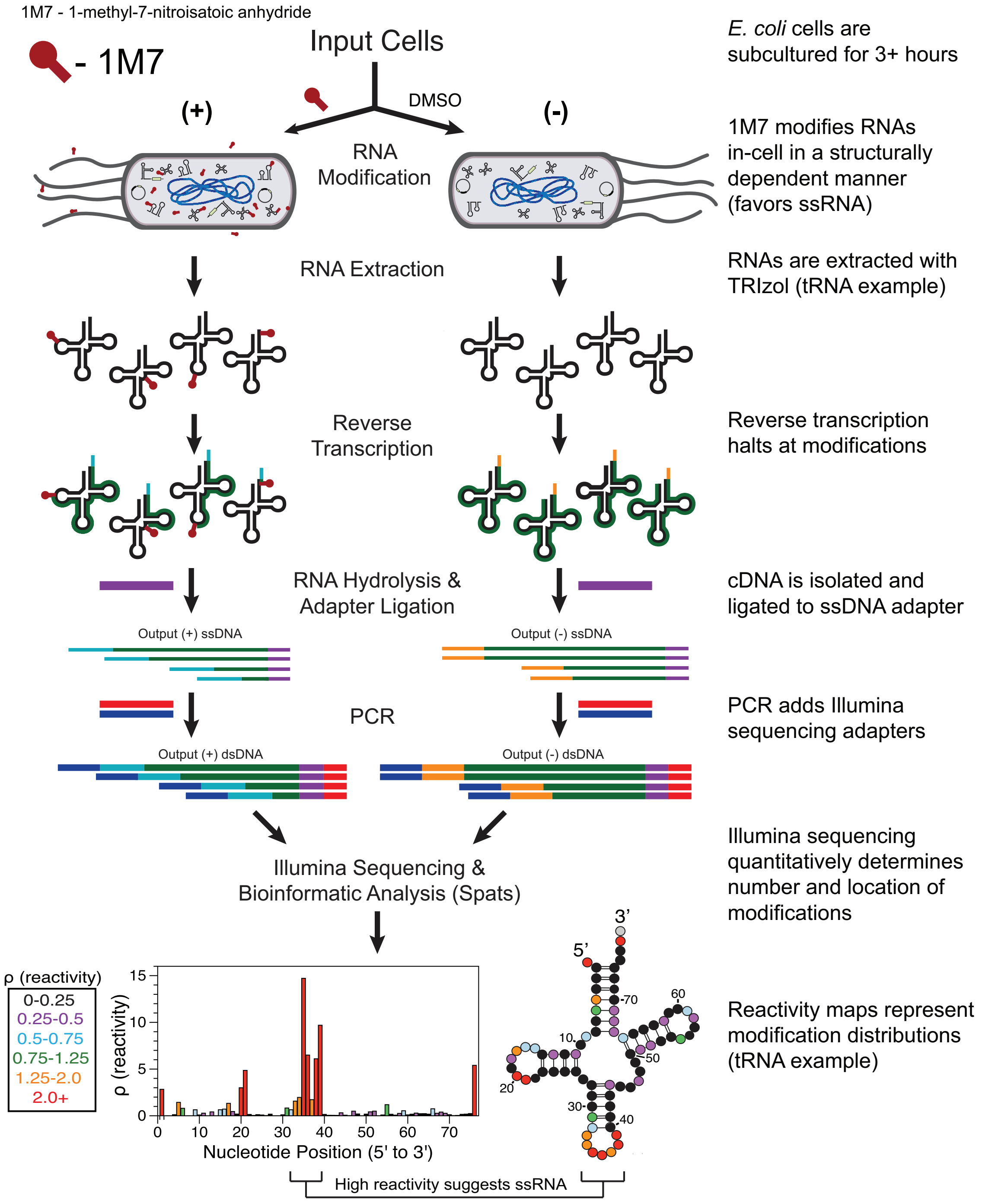

Supplementary Table 1. List of terminators screened for building the antisense platform. During design of the antisense platform, we sought a small intrinsic terminator that would serve as both an RT priming site and an efficient terminator of *E. coli* transcription. Ten different terminators with varying stem-loop properties were tested for RT priming site capability and the maintenance of ON/OFF functionality of the sense-antisense pairs. A double terminator strategy was ultimately used, combining the high termination efficiency of t500 with the RT priming capability of ECK120051404.

Terminator	Source	Sequence
t500	Phage 82 mut	CAAAGCCCGCCGAAAGGCGGGCTTTTTTTT
T7 RNA pol gene	T7 phage	CTGGCTCACCTTCGGGTGGGCCTTTCTGCGTTTATAAGG
T7 RNA pol	T7 phage	CCCTTGGGGCCTCTAAACGGGTCTTGAGGGGTTTTTT
T3 RNA pol	T3 phage	CTGGCTCACCTTCACGGGTGGGCCTTTCTTCGTTCCGGGCA
pT181	*S. aureus*	CGATTCCTTAAACGAAATTGAGATTAAGGAGTCGCTCTTTTTT
ECK120051404	Synthetic (2)	CCTCTACCTGCTTCGGCCGATAAAGCCGACGATAATACTCC
ECK120010812-R	Synthetic (2)	AACGGTTTATTAGTCTGGAGACGGCAGACTATCCTCTTCCC
ECK120010840	Synthetic (2)	CGTACCAGGCCCCTGCAATTTCAACAGGGGCCTTTTTTTATCC
tryptophan attenuator L126	*E.coli* (2)	ACCCAGCCCGCCTAATAAGCGGGCTTTTTTTTGAACA
p81	*S. aureus*	GCGGGGAATGTATACAGTTCATGTATATATTCCCCGCTTTTTTTTT

Supplementary Figure 3. Selective PCR amplification strategy for cDNA libraries. The cDNA generated from the reverse transcription step of in-cell SHAPE-Seq is ligated to an adapter that contains part of the sequence required for Illumina TruSeq barcoding (Supplementary Figure S2). To amplify the ssDNA library, primers containing the rest of the sequences for Illumina TruSeq barcoding are added to a PCR reaction (red, blue), along with a selection primer (purple). The selection primer contains several nucleotides on its 3' end that extend into the expected cDNA sequence. In this way, unextended RT primer ligated to excess adapter (dimer side product) is unable to extend due to a 3' mismatch (right). This selective PCR greatly reduces the amount of dimer side product that is amplified and removes the need for gel extraction.

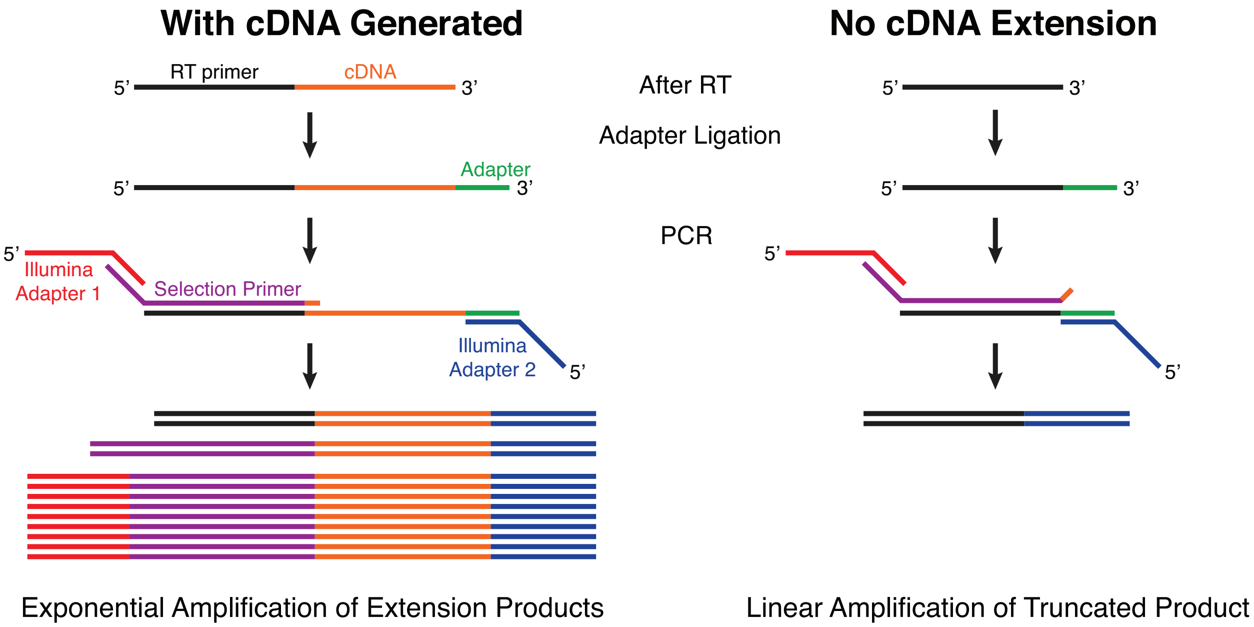

Supplementary Figure 4. Mechanism of the synthetic translation-activating riboregulator system. In the riboregulator system, the *cis*-repressed sense RNA (crRNA) is designed to form a hairpin structure that occludes the RBS, blocking translation. The riboregulator is thus in the OFF state when expressed by itself. A *trans*-activating antisense RNA (taRNA) is designed to base pair with the 5' region of the crRNA through a loop-linear interaction intermediate to expose the RBS on the crRNA and allow translation. The riboregulator switches to the ON state in the presence of a cognate taRNA (5).

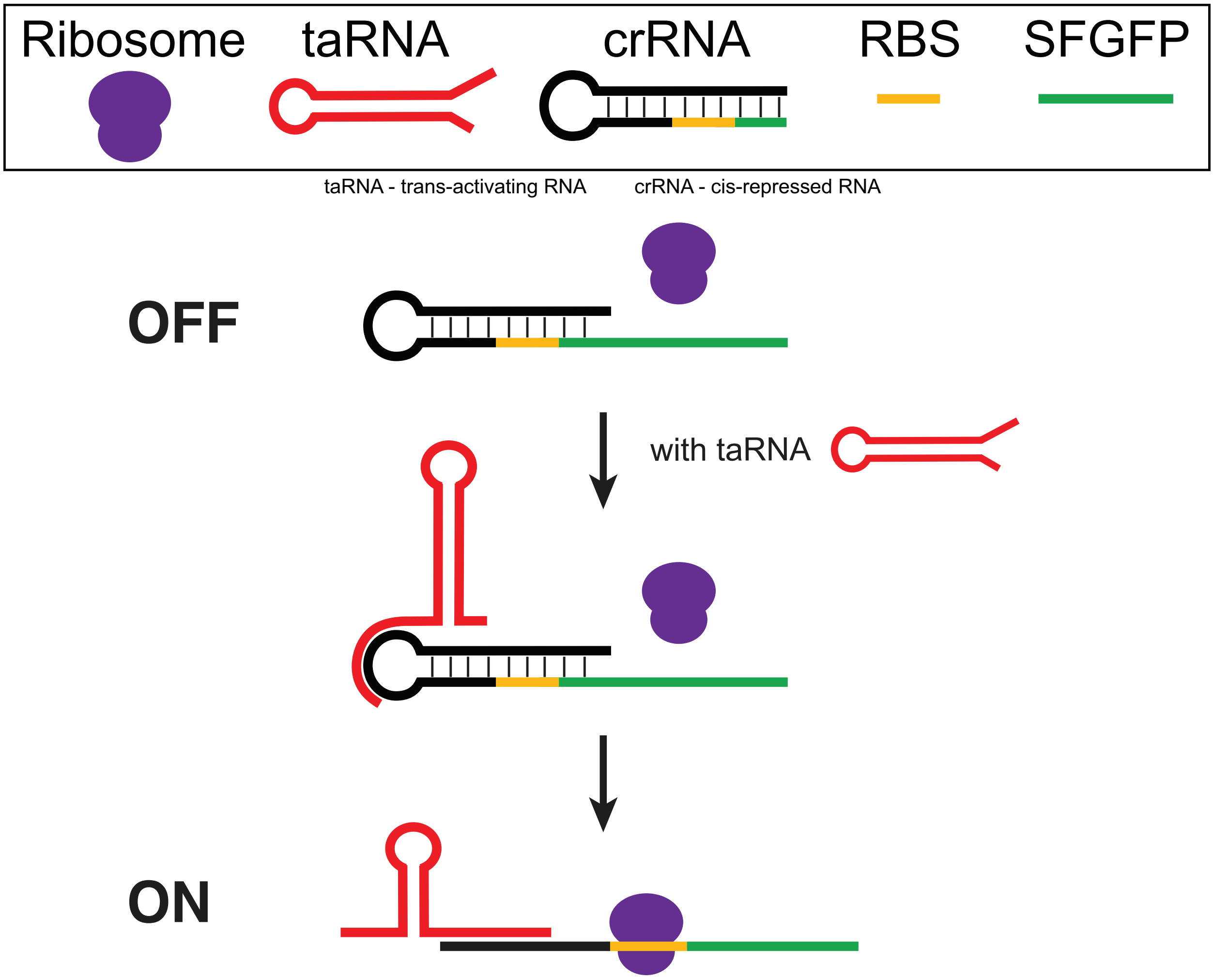

Supplementary Figure 5. Structural analysis of the synthetic riboregulator translational activation system using in-cell SHAPE-Seq data. Structures originally designed by Isaacs, et al. (5) for riboregulator sets taR12/crR12 and taR10/crR10 compared to models of the cellular structural states generated from in-cell SHAPE-Seq reactivities. The reactivities were used to constrain the secondary structure prediction program RNAStructure (6). Nucleotide locations where the structures differ are highlighted in yellow (left). (a) Constraining the crR12 fold with the average reactivities calculated from in-cell SHAPE-Seq indicates that the apical loop may be larger than originally predicted in cells. There are few differences between the predicted and constrained folds for the highly structured taR12: the U bulge near position 50 is in a different position in the cell, and the inner loop near position 20 is predicted to be larger in cells. (b) The same analysis for taR10/crR10. This analysis also found that the apical loop of crR10 was likely less structured in the cell than originally predicted and the same U bulge in taR10 near position 50 was in a different location. However, the taR10 inner loop near positions 22-23 matched the structures predicted by Isaacs, et al. (5, 6).

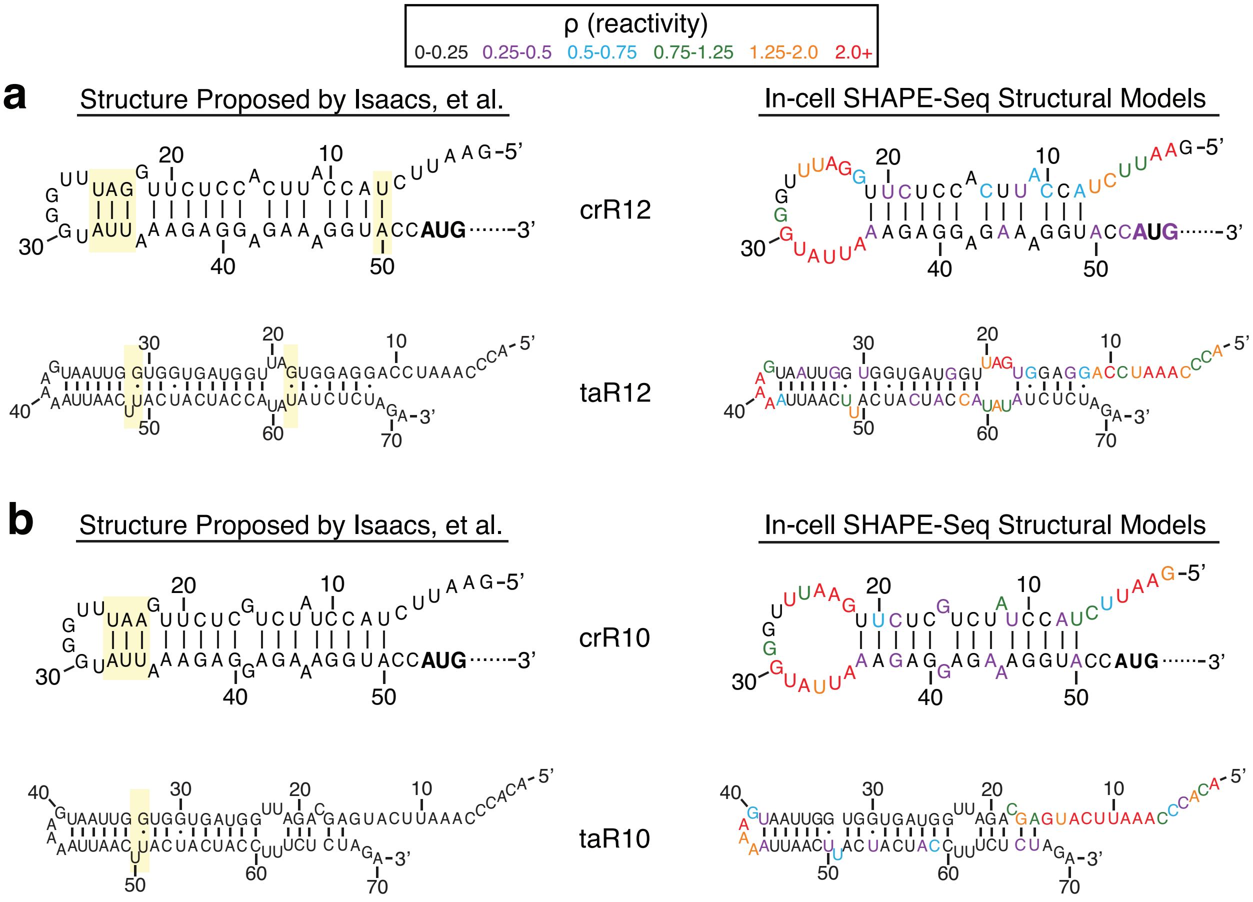

Supplementary Figure 6. Characterization of the cellular structures of the taR10/crR10 synthetic riboregulator RNA translational activator system. Reactivity maps and constrained secondary structure folds are shown for both taR10 (a) and crR10 (b) of the synthetic riboregulator activator system. Color-coded reactivity spectra represent averages over three independent in-cell SHAPE-Seq experiments, with error bars representing one standard deviation of the reactivities at each position. RNA structures represent minimum free energy structures generated by RNAStructure (5, 6) using in-cell SHAPE-Seq reactivity data as constraints (see Methods). Nucleotides are color-coded by reactivity intensity. Comparisons to original structural designs from Issacs, et al. (5) are shown in Supplementary Figure S5. The crR10 structural model was generated from the first 70 nts of the sequence (55 nt shown), and the terminators following taR10 were not included in the structural analysis. The start codon (AUG) location is boxed and the coding sequence (CDS) is labeled.

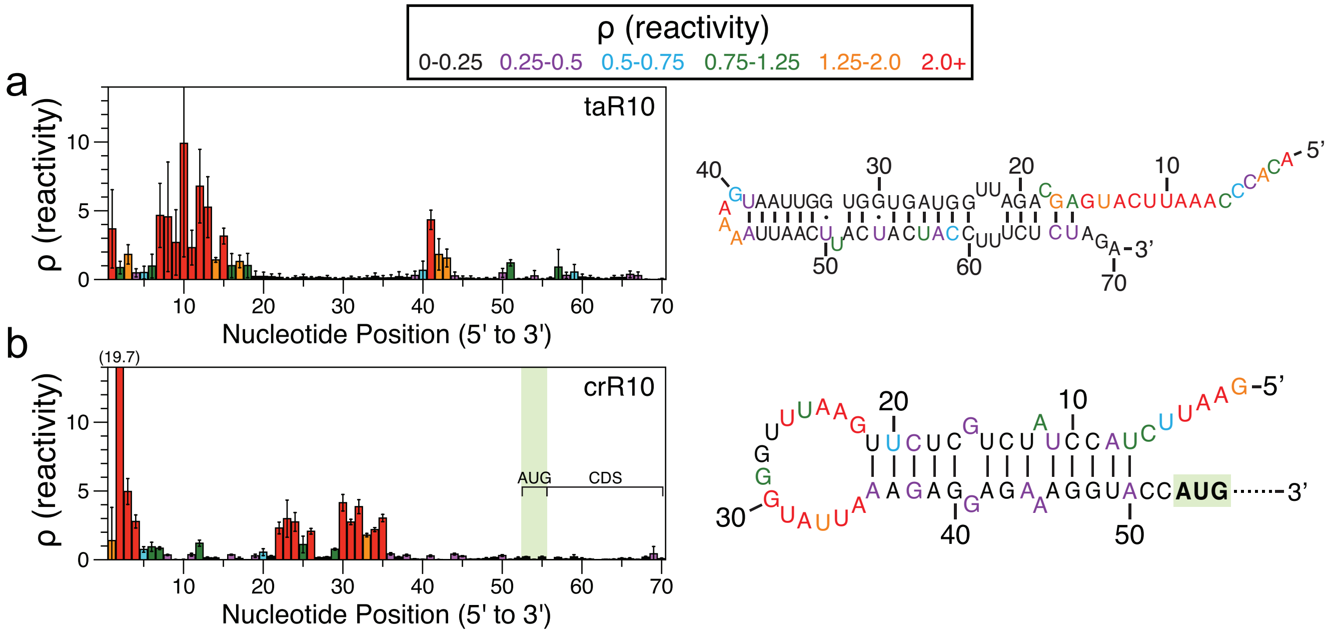

Supplementary Figure 7. Characterization of the cellular structures of the taR12/crR12 synthetic riboregulator RNA translational activator system using in-cell dimethyl sulfate (DMS) probing. Reactivity maps for an in-cell dimethyl sulfate (DMS) probing experiment (see Methods) are shown for taR12 (a) and crR12 (b) of the synthetic riboregulator system. Color-coded reactivity spectra represent the reactivity level at each position, calculated in the same way as in-cell SHAPE-Seq reactivity data. G and U positions are marked with gray, as DMS reacts with strong preference for As and Cs (7). The structures presented are the same from Figure 2 with DMS reactivities overlaid and Gs and Us marked with gray. The start codon (AUG) in crR12 is boxed and the coding sequence (CDS) is labeled. In general, there is good agreement between the in-cell DMS reactivities shown here and the in-cell SHAPE reactivities displayed in Figure 2 at A and C positions.

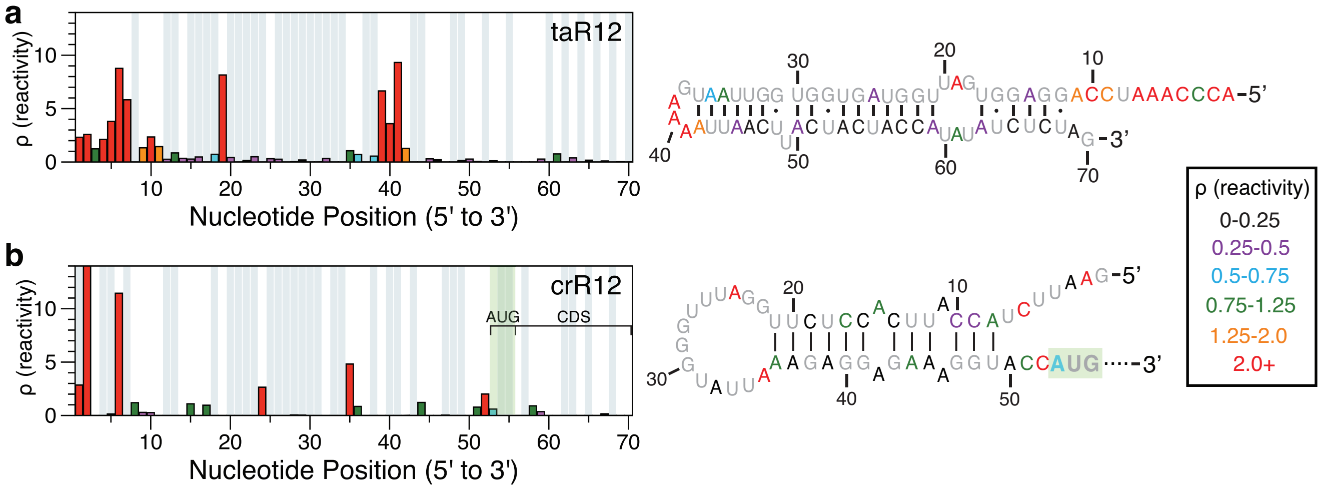

Supplementary Figure 8. Structure-function characterization of the taR10/crR10 synthetic riboregulator RNA translational activator system. Reactivity maps (a) and a suggested RNA-RNA interaction structure with taR10 (b) are shown for crR10 of the synthetic riboregulator activator system. (a) Color-coded reactivity spectra for crR10 expressed in conjunction with the control plasmid or taR10. Reactivities represent averages over three independent in-cell SHAPE-Seq experiments, with error bars representing one standard deviation. Average fluorescence (FL/OD) values (normalized to crR10 expressed with the control antisense plasmid) on the right show a 5-fold activation of gene expression when taR10 interacts with crR10, with error bars representing one standard deviation. The RBS (determined in Supplementary Figure S10) and start codon (AUG) locations are boxed. (b) Structural model of the crR10/taR10 binding complex derived from the mechanism proposed by Isaacs, et al. (5), refined using the average crR10 reactivities with taR10 present in (a). Nucleotides for crR10 are color coded by reactivity intensity. (c) Reactivity and functional data of the RBS region, showing an increase in RBS reactivity (left) and fluorescence (right) when taR10 is co-expressed with crR10. Positions that are statistically significantly different ($p < 0.05$) according to a one-sided Welch's t-test are indicated with *.

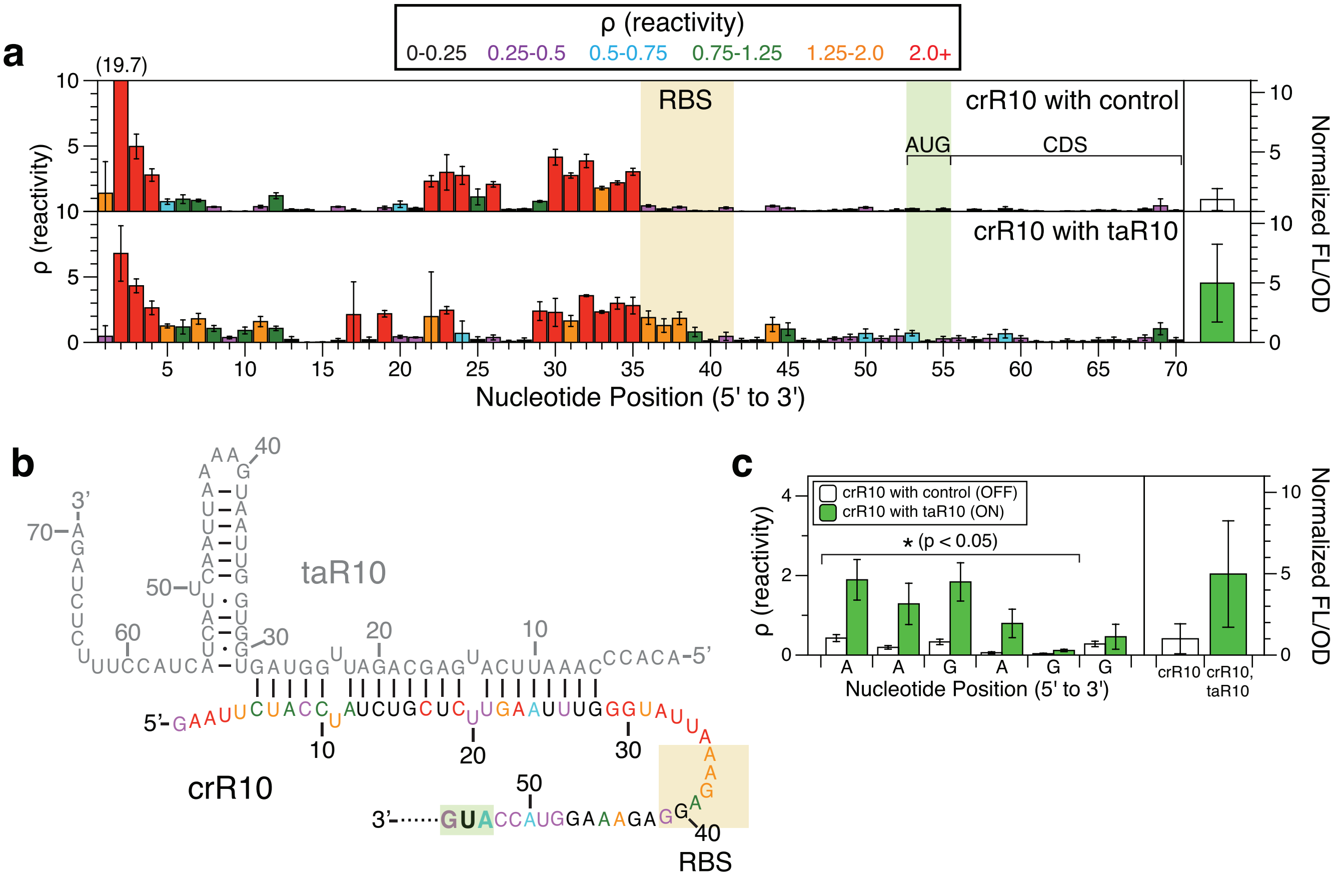

Supplementary Figure 9. In-cell SHAPE-Seq reactivities for the trans-activating RNA variants. In-cell SHAPE-Seq reactivity spectra for variants taR12 and taR10 of the trans-activating RNAs from the riboregulator system (5). Color-coded reactivity spectra represent averages over three independent in-cell SHAPE-Seq experiments, with error bars representing one standard deviation of the reactivities at each position. (a) The reactivity spectra for the taR12 variant with and without its cognate crR12 show clusters of high reactivity in the GAAA loop (nt 39-43) and at the 5' end, which is predicted to be single-stranded when expressed alone (Supplementary Figure S5) and interact with the crR12 apical loop when it is expressed with crR12. Yet, no major changes in reactivity are observed when crR12 is present, likely due to the higher copy number of taR12 in the cell relative to crR12. (b) Reactivity spectra for the taR10 variant with and without the cognate crR10. The pattern is similar to that of taR12 above and also shows little change in the presence of the sense RNA crR10. The GAAA tetraloop is in between nucleotides 40-44 in taR10.

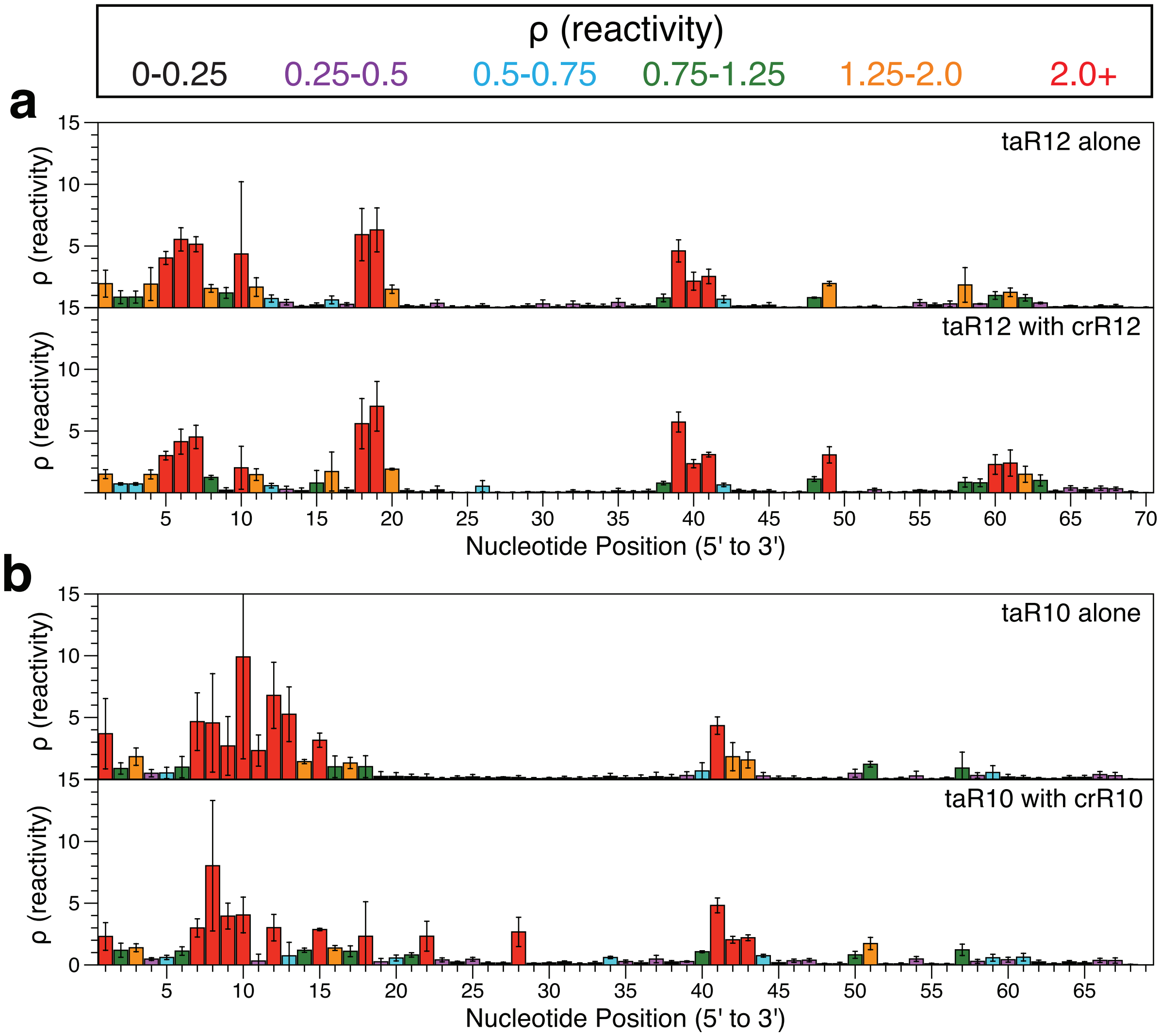

Supplementary Figure 10. Determining the dominant RBS sequence in crRNAs using in-cell SHAPE-Seq reactivities. Each crRNA contains an AG-rich region with the potential to contain multiple six-nucleotide Shine-Dalgarno (SD) sequences (8). (a) To determine which six nucleotides comprise the dominant SD sequence in crR12, a sliding window was used to analyze reactivity changes in the AG-rich region when crR12 is interacting with taR12. The reactivity of each window was calculated by summing the reactivities for the six nucleotides in the window. Windows were calculated separately for the OFF (crR12 with control plasmid) and ON (crR12 and taR12) states. The differences between the ON and OFF state for each window were used to determine which window displayed the largest change in reactivity. Nucleotides 36-41 of crR12 demonstrated the largest increase in reactivity (3.88) and are one nucleotide away from the consensus AGGAGG, suggesting 36-41 is likely the dominant SD sequence. (b) The same analysis was used to find the dominant SD sequence for crR10. The 36-41 nucleotide window was again found to have the highest increase in reactivity (5.07).

a

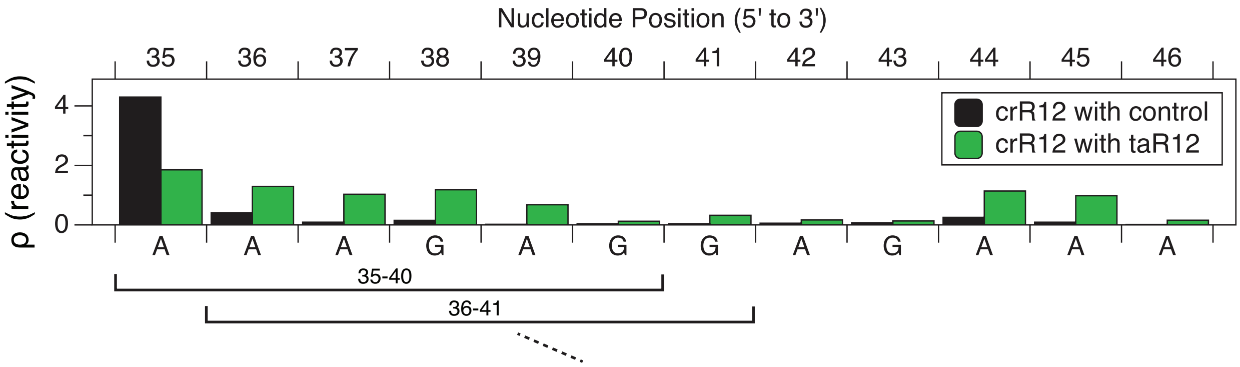

Shine-Dalgarno Reading Frame Reactivity

	35-40	36-41	37-42	38-43	39-44	40-45	41-46
crR12 with control	5.01	0.75	0.40	0.38	0.48	0.55	0.53
crR12 with taR12	6.16	4.63	3.50	2.60	2.56	2.86	2.90
Difference in ρ	1.15	**3.88**	3.10	2.22	2.08	2.31	2.37

b

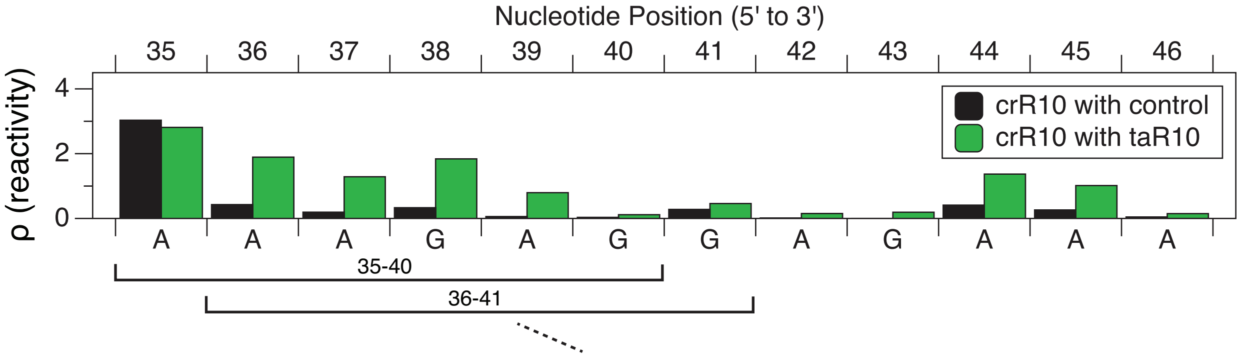

Shine-Dalgarno Reading Frame Reactivity

	35-40	36-41	37-42	38-43	39-44	40-45	41-46
crR10 with control	4.08	1.32	0.91	0.72	0.80	1.00	1.01
crR10 with taR10	8.74	6.39	4.65	3.55	3.08	3.30	3.34
Difference in ρ	4.66	**5.07**	3.74	2.84	2.29	2.30	2.33

Supplementary Figure 11. Mechanism of the translation-repressing RNA-IN/OUT system. (a) In the RNA-IN/OUT system modified by Mutalik, et al. (9), the sense RNA (RNA-IN) is expressed upstream of SFGFP and contains an exposed RBS that allows the translation of SFGFP in the ON state. When the antisense RNA (RNA-OUT) is present, RNA-IN is predicted to base-pair to the 5' half of RNA-OUT, causing the RBS to become double-stranded and block the translation of SFGFP, switching to the OFF state. As depicted, RNA-OUT is predicted to be an extended stem-loop structure with two inner bulges (9). (b) By mutating the interaction region between RNA-IN (first 5 nt of 5' end) and RNA-OUT (apical loop), Mutalik, et al. (9) generated many orthogonal, or independently acting, pairs of regulators. For example, the RNA-IN variant S3 will be repressed by A3, but the mutations between A3 and A4 do not allow A4 to significantly repress S3.

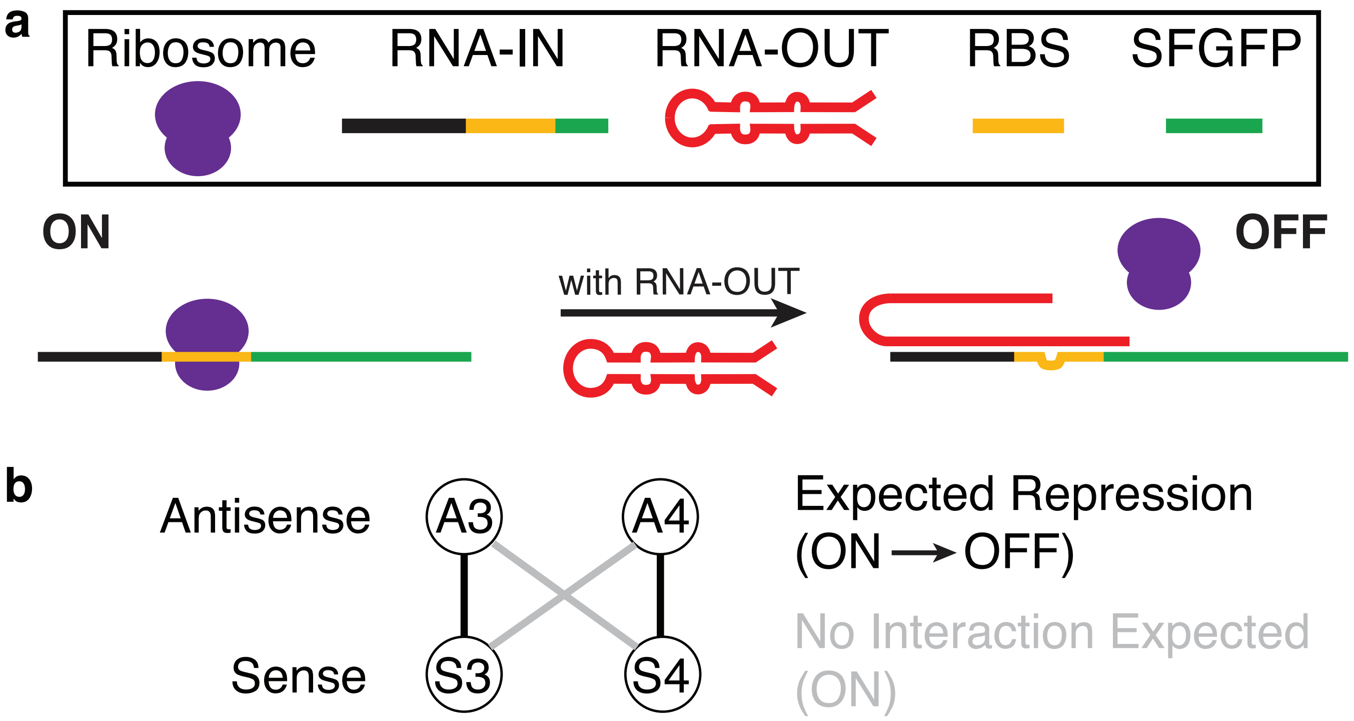

Supplementary Figure 12. Characterization of the cellular structures of the S3/A3 RNA-IN/OUT translational repressor system. Color-coded reactivity spectra are averaged over three independent in-cell SHAPE-Seq experiments, with error bars representing one standard deviation of the reactivities at each position. RNA structures represent minimum free energy structures generated by RNAStructure (6) using in-cell SHAPE-Seq reactivity data as constraints (see Methods). Nucleotides are color-coded by reactivity intensity. (a) Reactivity spectrum of the first 60 nts of RNA-IN S3 (top), with nucleotides color-coded by reactivity on a single-stranded structural model of this region (bottom). RBS and start codon (AUG) are boxed. (b) Reactivity spectra (top) and reactivity-constrained secondary structure model (bottom) for RNA-OUT A3 (antisense). As observed with RNA-OUT A4 in Figure 4b, the interacting loop is predicted to be larger than originally proposed by Mutalik, et al. (9, 10) when SHAPE constraints are applied. The terminators following RNA-OUT were not included in the structural analysis. CDS = coding sequence.

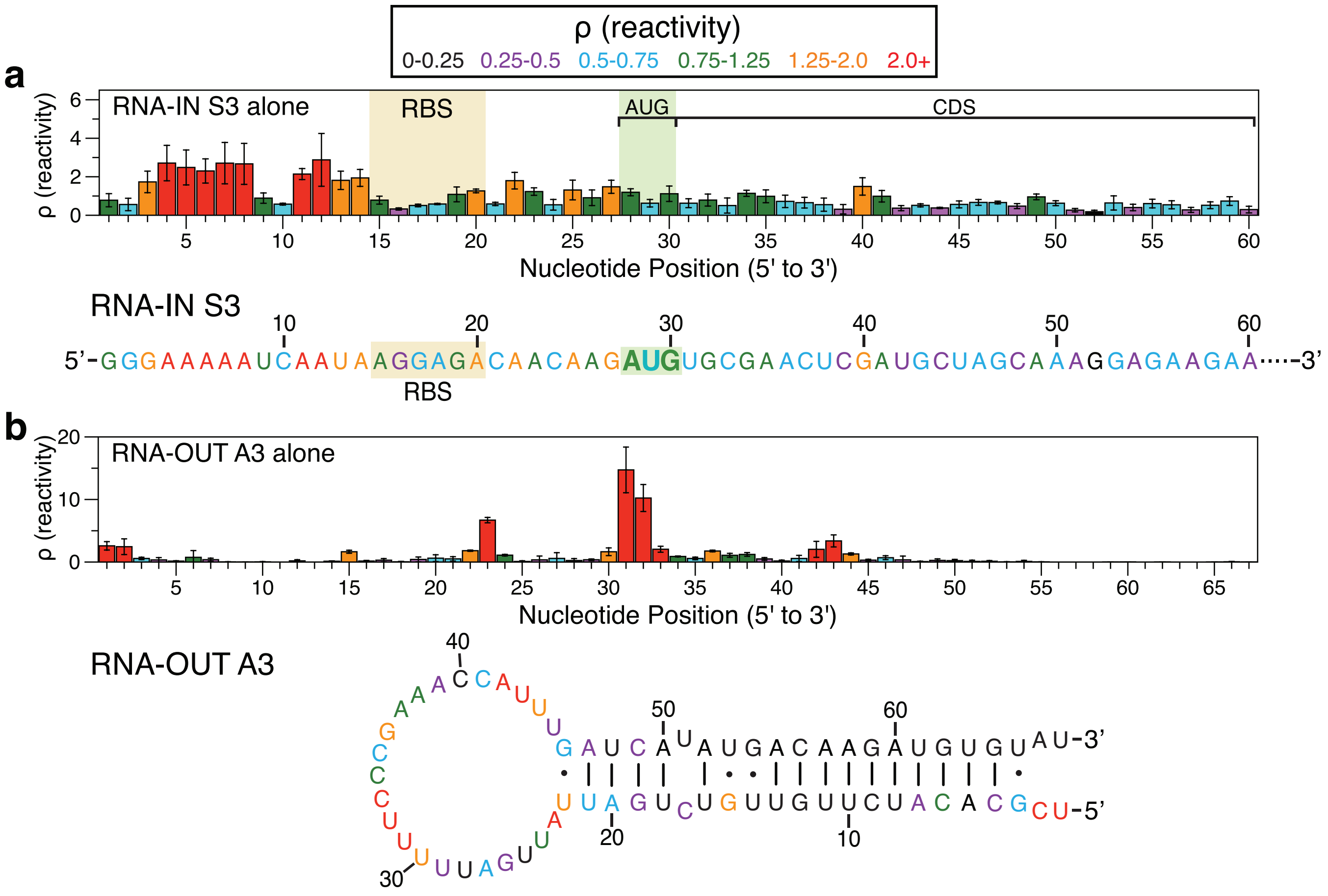

Supplementary Figure 13. RNA-IN/OUT S4/A4 interaction complexes appear to be cleaved by a double-stranded RNase in the cell. Reactivity maps (colored bars), (-) control fragment distributions (black bars), and percent expression (white bars) for three independent replicates of in-cell SHAPE-Seq experiments co-expressing both RNA-IN S4 and RNA-OUT A4. Despite all of the replicates having a similar level of translational repression, the reactivity maps have different patterns. We hypothesized that this was caused by the large peak aligning to nucleotide 26 in the (-) control fragment length distributions in each replicate. The wt RNA-IN/OUT interacting duplex is known to contain an RNase III cut site before nucleotide 15 in RNA-IN (9, 10), although this cut site was removed in our system by Mutalik, et al. by mutating nucleotides 15 and 16 to 'GG', causing a local mismatch with RNA-OUT (see Supplementary Figure S16) (9, 10). We hypothesized that there may be a second cut site for a double-stranded RNase between nucleotides 25 and 26, which would prevent reverse transcriptase from reading all the way to the 5' end of RNA-IN. If true, this would suggest that our measurement would be probing two major states of the interacting RNAs between nucleotides 1-25 at once: the uncut and cut - states. Variability in the relative level of dsRNA cleavage would then cause the variability in the reactivity maps.

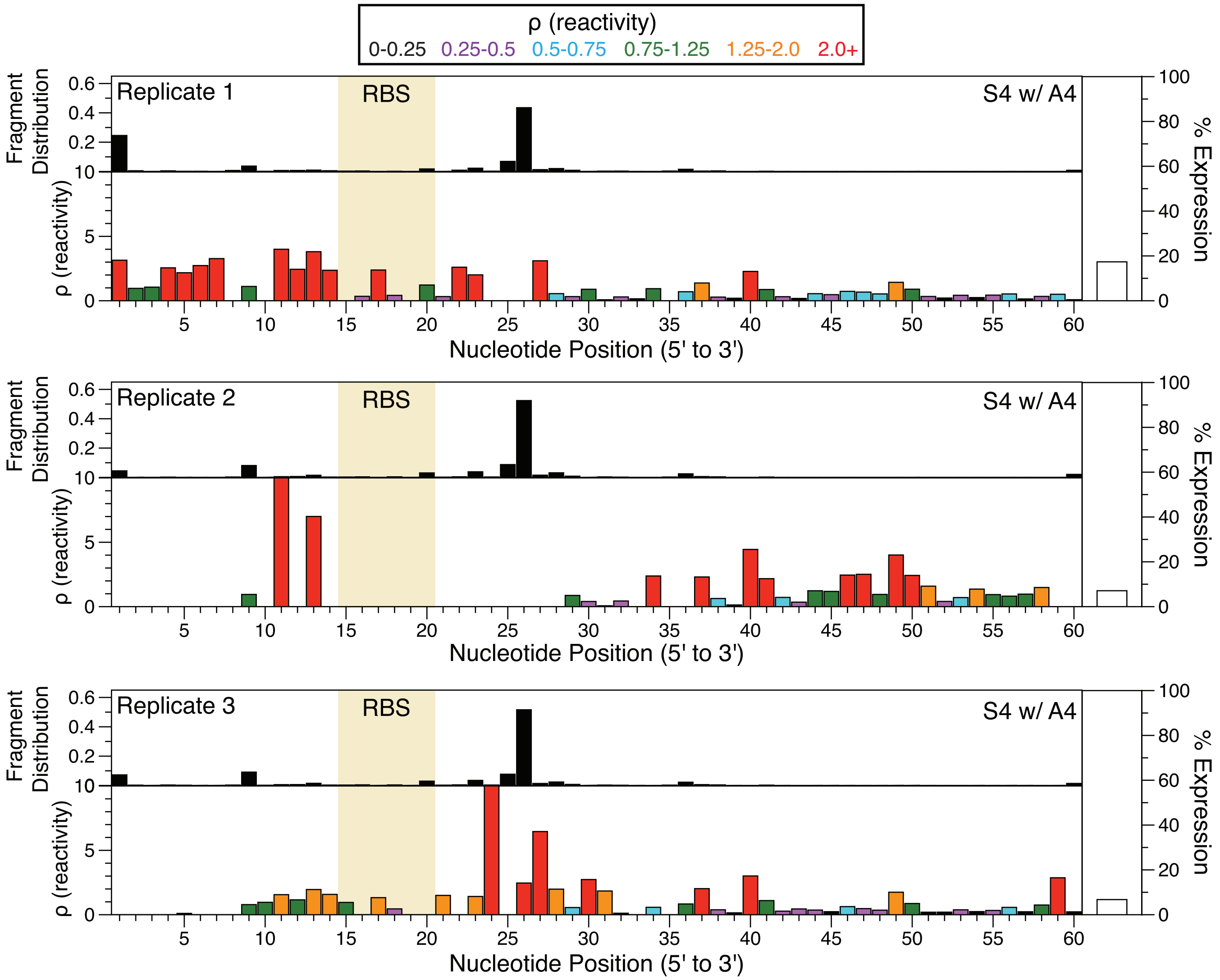

Supplementary Figure 14. Structure-function analysis of the S4/A3 interaction from the RNA-IN/OUT translational repressor. Color-coded reactivity spectra represent averages over three independent in-cell SHAPE-Seq experiments, with error bars representing one standard deviation of the reactivities at each position. (a) Reactivity maps and fluorescence (FL/OD) of RNA-IN S4 (normalized to average S4 alone fluorescence) expressed alone (top) and with RNA-OUT A3 (bottom). RBS and start codon (AUG) are boxed. Note that RNA-OUT A3 is orthogonal to S4 and should therefore not cause a change in gene expression. As expected, the reactivity patterns and fluorescence of S4 do not show major changes between the two conditions, indicating that the two RNAs are indeed not interacting. (b) (-) control fragment distributions for one replicate of the libraries sequenced to produce the reactivity maps in (a). Note that the degradation peak observed at nucleotide 26 (Supplementary Figure S13) is not observed for the non-interacting RNAs and thus is not due to the fact that both RNA-IN and RNA-OUT variants were co-expressed. CDS = coding sequence.

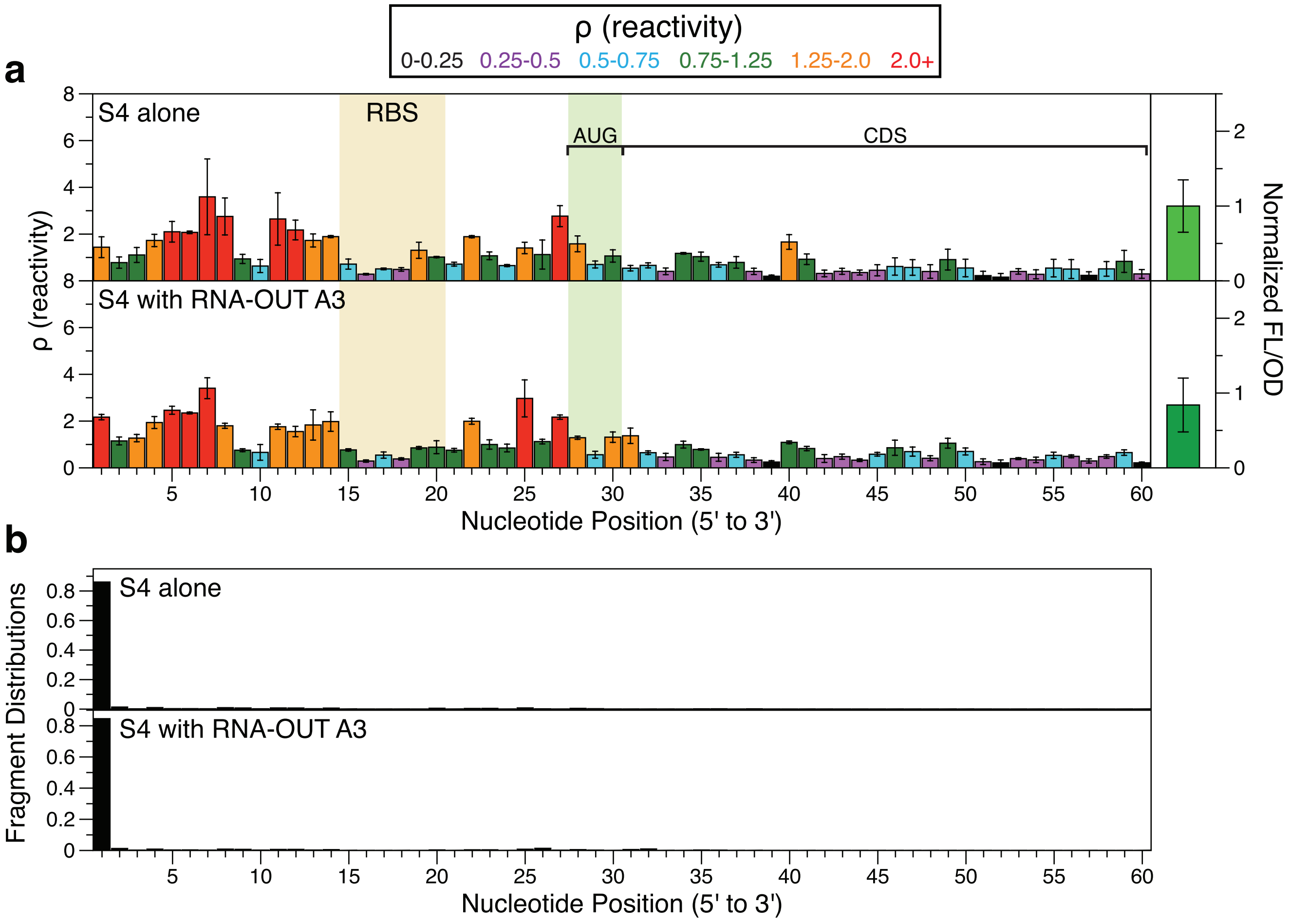

Supplementary Figure 15. Structure-function analysis of the S3/A4 interaction from the RNA-IN/OUT translational repressor. Color-coded reactivity spectra represent averages over three independent in-cell SHAPE-Seq experiments, with error bars representing one standard deviation of the reactivities at each position. (a) Reactivity maps and fluorescence (FL/OD) for RNA-IN S3 (normalized to average of S3 fluorescence) expressed alone (top) and with RNA-OUT A4 (bottom). RBS and start codon (AUG) are boxed. Unlike for the S4/A3 pair (Supplementary Figure S14), the S3/A4 pair shows small changes in the reactivity maps of RNA-IN S3 and in the expression of SFGFP in the presence of RNA-OUT A4, although both exhibit a higher level of noise. (b) (-) control fragment distributions for one replicate of the libraries sequenced to produce the reactivity maps in (a). The fragment distribution shown for the S3/A4 interaction is for a replicate deviating somewhat from the other two in terms of nucleotide reactivities and fluorescent output, generating most of the observed noise. CDS = coding sequence.

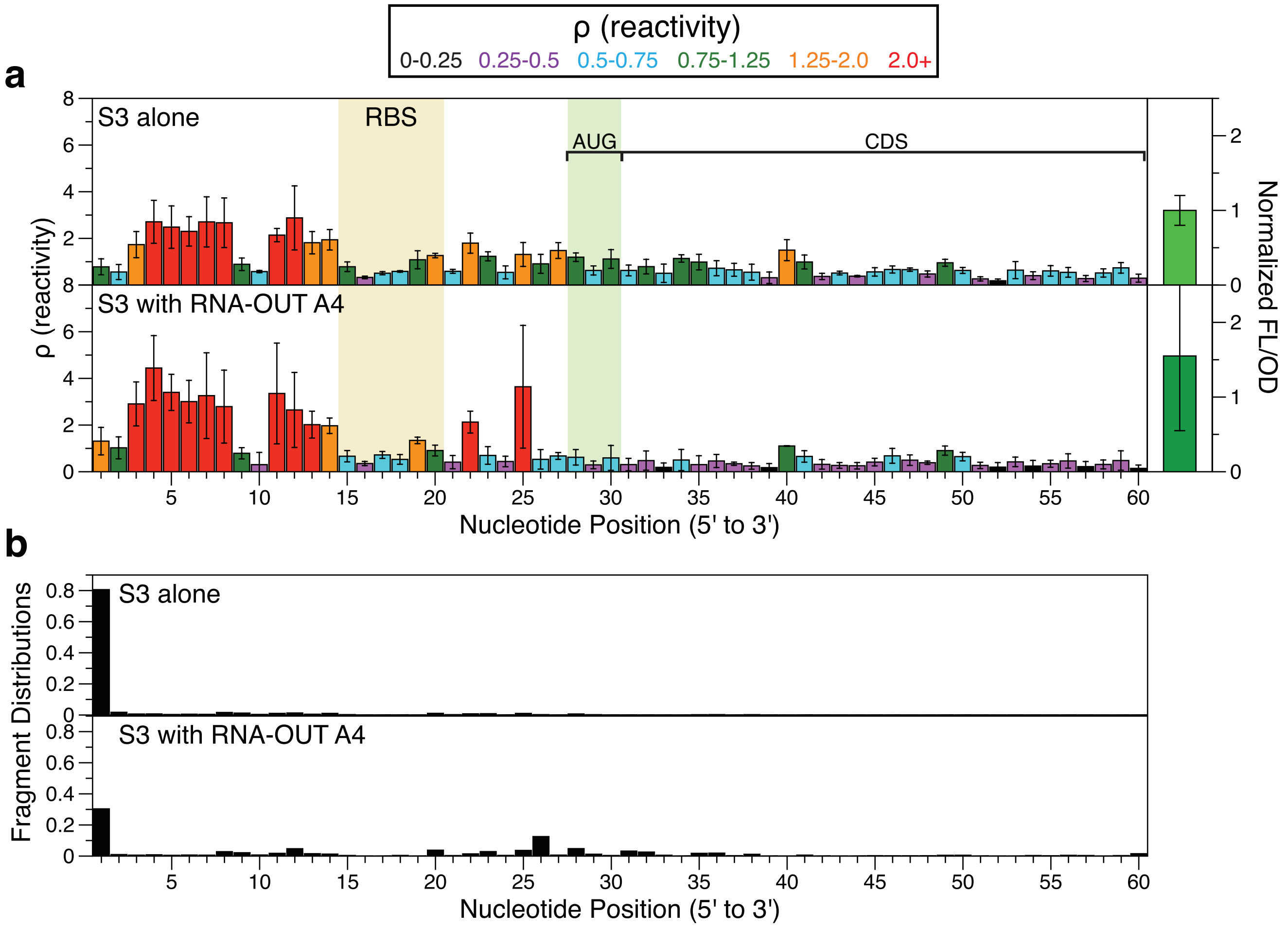

Supplementary Figure 16. RNA-IN mutations to resist RNase cleavage between nucleotides 25 and 26. The structure on the far left depicts the interaction between RNA-IN S4 and RNA-OUT A4 as proposed by Mutalik, et al. after they blocked a known RNase III cleavage site with an interior loop (9, 10). Suspecting that RNase III may also be involved in the observed cleavage between RNA-IN nucleotides 25 and 26 (Supplementary Figure S13), we mutated the positions that would most directly block RNase III cleavage between RNA-IN and RNA-OUT. We introduced the C24A and A25C mutations, tested them (Supplementary Figure S17), and ultimately characterized the double mutant in triplicate with in-cell SHAPE-Seq (Figure 4c, Supplementary Figure S18).

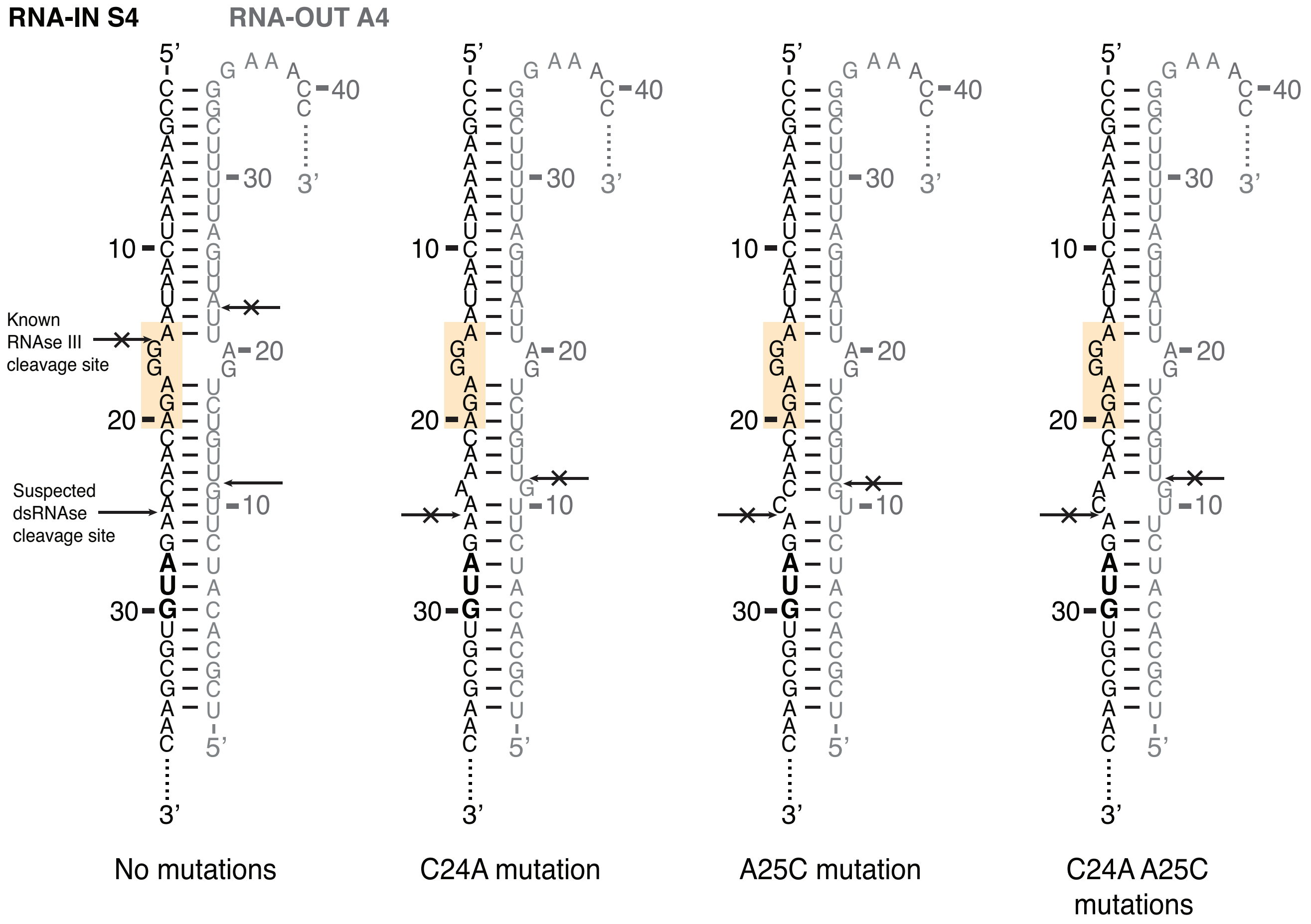

Supplementary Figure 17. RNA-IN S4 mutations resist cleavage and maintain functionality. Color-coded reactivity spectra represent one independent in-cell SHAPE-Seq experiment with fluorescence (FL/OD) measurements on the right (ON state normalized to one). (a) The RNA-IN S4 A25C mutant exhibits ~70% repression, which is lower than the original S4. However, mutation A25C does not exhibit a peak in the fragment distribution at nucleotide 26 in the presence of RNA-OUT A4, as was observed with the original S4 sequence in Supplementary Figure S13. Reactivities decrease near the 5' end, and increase modestly between nucleotides 21-27 when S4 A25C interacts with A4. (b) RNA-IN S4 mutant C24A shows a similar reactivity decrease at the 5' end as S4 A25C in (a) and the absence of a peak in the fragment distribution at nucleotide 26 in the presence of RNA-OUT A4. However, the C24A mutant exhibited better repression (87%). (c) The C24A A25C double mutant shows the same general features of (a) and (b), and was used for replicate in-cell SHAPE-Seq experiments (Figure 4c). RBS and start codon (AUG) are boxed. CDS = coding sequence.

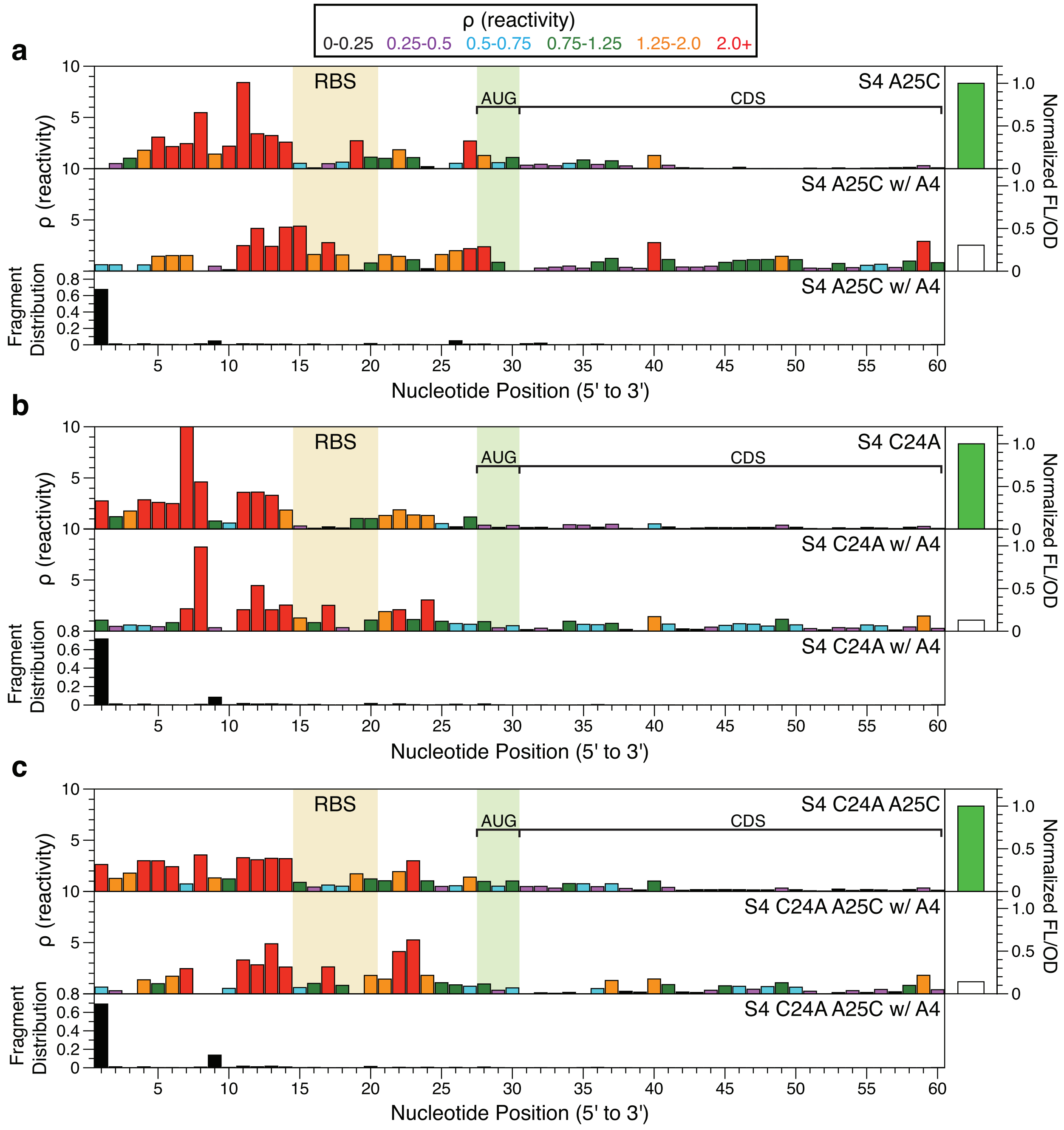

Supplementary Figure 18. Ribosome binding site analysis of RNA-IN S4 mutant C24A A25C. The fluorescence measured (FL/OD; left) and ribosome binding site (RBS) reactivities (right) of RNA-IN S4 mutant C24A A25C represent the average of three independent in-cell SHAPE-Seq experiments, with error bars representing one standard deviation. The S4 C24A A25C RBS nucleotides show significant reactivity changes in the presence of RNA-OUT A4 at nucleotides 16 and 17 ($p < 0.10$), which increase in response to antisense A4 binding. The increase in nucleotides 16 and 17 is expected due to the designed double bulge at the previously known RNase III cut site (9, 10). Positions that are significantly different ($p < 0.10$) according to a one-sided Welch's t-test are indicated with *.

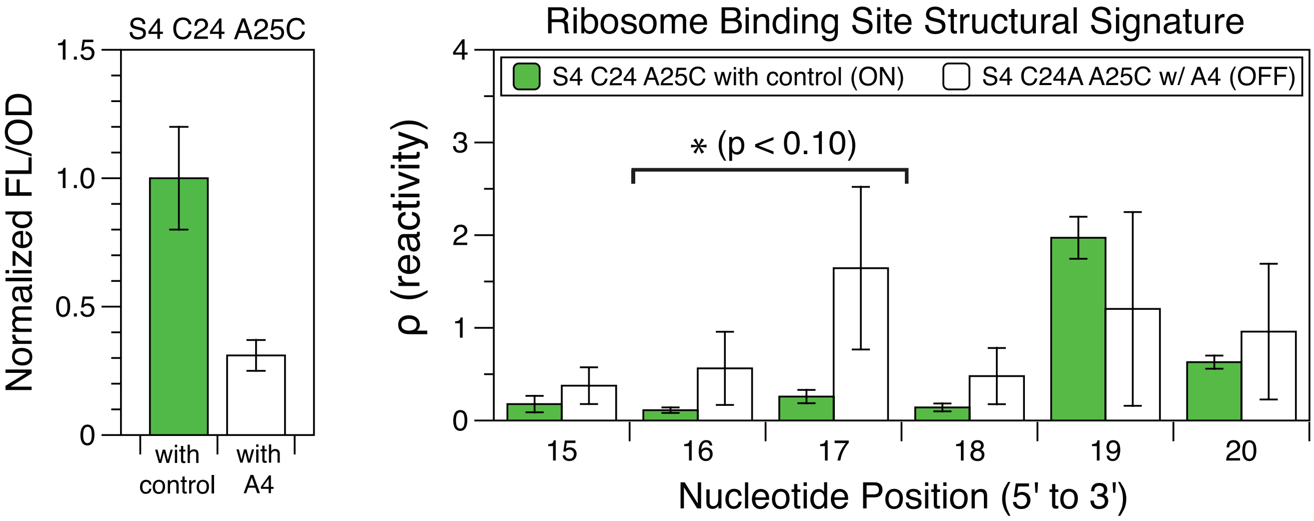

Supplementary Figure 19. In-cell SHAPE-Seq reactivities for the antisense RNA-OUT A4 with RNA-IN variants. Color-coded reactivity spectra represent averages over three independent in-cell SHAPE-Seq experiments, with error bars representing one standard deviation of the reactivities at each position. The SHAPE-Seq reactivities of RNA-OUT A4 are shown without RNA-IN, with RNA-IN S3 (remains ON), and with RNA-IN S4 mutant C24A A25C (turns OFF). There are no major differences between A4 alone or with the orthogonal S3. When S4 C24A A25C is present to interact with A4, there are decreases in the reactivities of nucleotides 1, 2, and 31-34 and increases in nucleotides 19, 26, 27, and 47. The nucleotides with decreasing reactivities correspond well to regions of A4 expected to interact with S4 C24A A25C (Supplementary Figure S16). However, the locations of increasing reactivities are generally unexpected except for nucleotide 19, which forms part of the double bulge originally designed to prevent RNase III cleavage (9). It should be noted that the copy number difference between the RNA-OUT and the RNA-IN plasmids should cause RNA-OUT to be present in excess, such that the dominating structure is likely the non-interacting one, lessening the changes that are observable for RNA-OUT binding to RNA-IN.

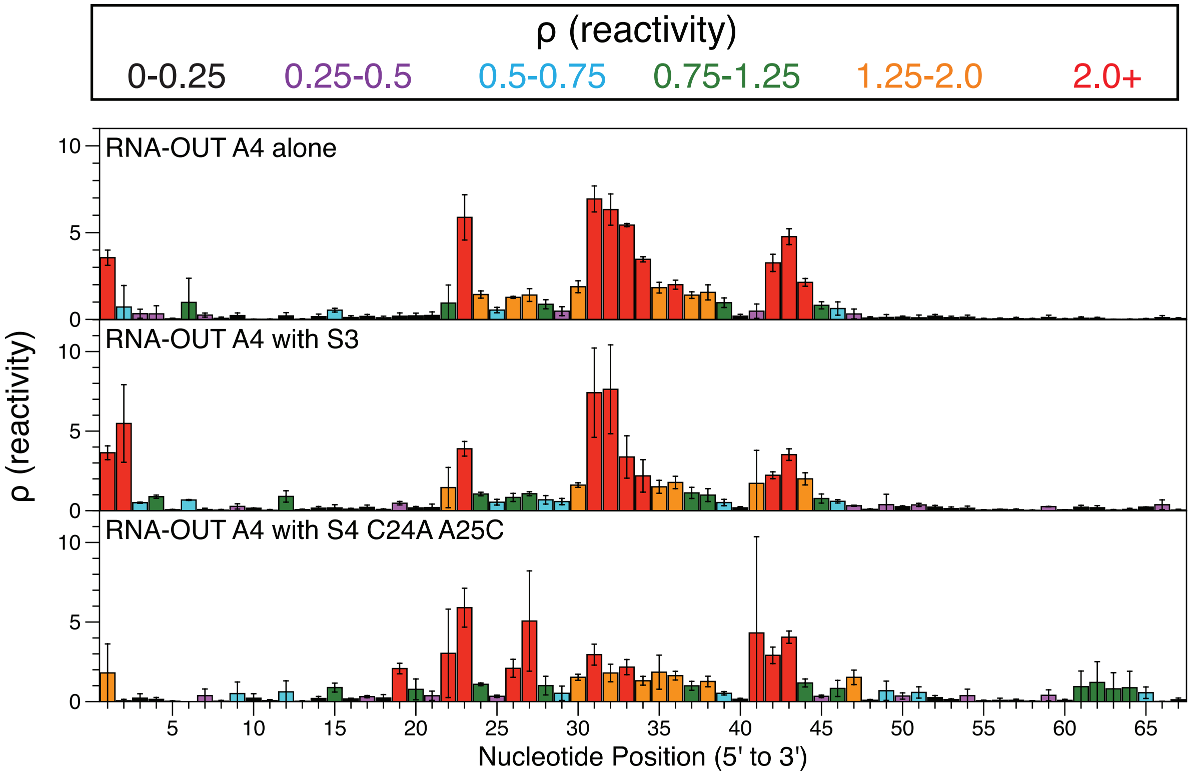

Supplementary Figure 20. Increasing PCR selection does not affect reactivity calculation. (a) Reactivity map comparing the same crR12 replicate (expressed alone) sequenced using 15 cycles of PCR with all oligonucleotides included (15x) or 15 cycles of PCR without oligonucleotide I (Supplementary Table S3) followed by another 15 cycles after adding oligonucleotide I. The reactivity maps show very close agreement as depicted in the correlation plot in (b), with a Pearson Correlation Coefficient of 0.996.

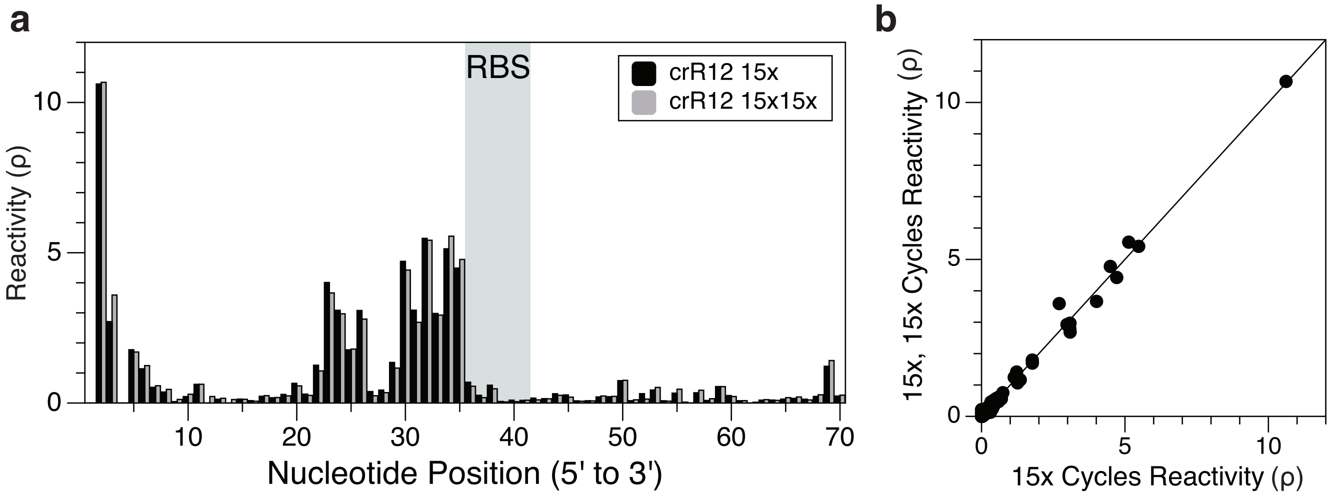

Supplementary Figure 21. Reactivity maps for endogenous RNA targets. Bar charts depicting the reactivity values for the 5S rRNA, RNase P specificity region, and *btuB* riboswitch domain used to color Figure 5. Color-coded reactivity spectra represent averages over three (five for *btuB* riboswitch) independent in-cell SHAPE-Seq experiments, with error bars representing one standard deviation of the reactivities at each position. (a) 5S rRNA shows clusters of reactivity in the 5' half when probed within the cell in contrast to equilibrium folded RNA *in vitro* (Supplementary Figure S24). The RT priming site on the 3' end, where no reactivity information was obtained, is marked. (b) The specificity region of RNase P shows well-distributed, highly reactive peak clusters. (c) Reactivity map for the *btuB* adenosylcobalamin riboswitch, which displays more unstructured nucleotides in the 5' half.

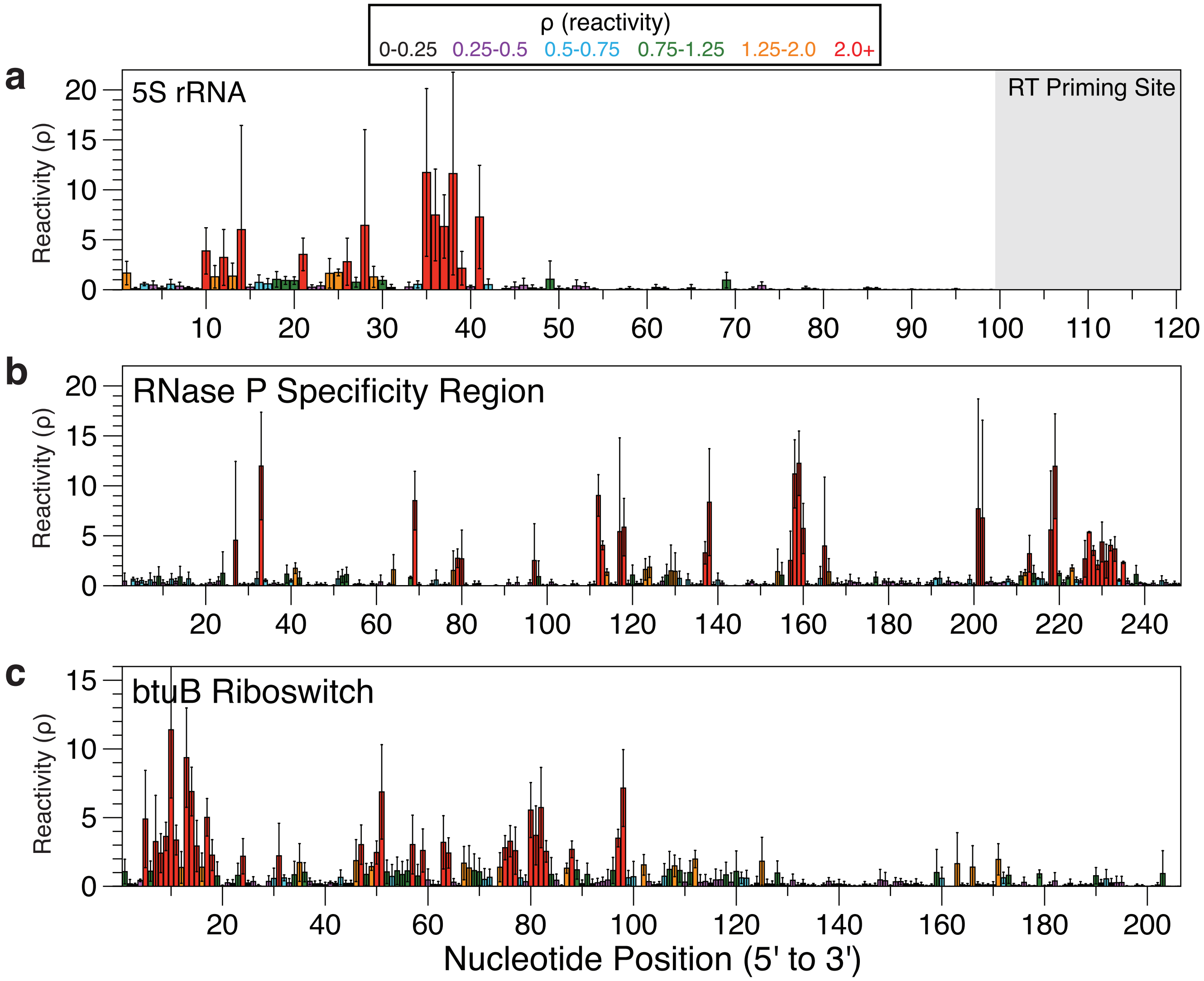

Supplementary Figure 22. In-cell vs. equilibrium *in vitro* refolding reactivity maps for the riboregulators. Bar charts comparing reactivities measured for the riboregulator variant 12 RNAs in-cell (measured in triplicate) and an equilibrium refolding experiment with *in vitro* purified RNA. Error bars for the in-cell measurements represent one standard deviation. (a) Few differences are observed for the crR12 RNA between in-cell (with control antisense plasmid) and equilibrium measurements. The most notable difference is near the 5' end where the intensities differ somewhat and the most 5' nucleotide is reactive *in vitro*. (b) Like the crR12 alone case in (a), there are few observable differences in the crR12 with taR12 case. We observe that the average reactivity between nucleotides 24-40 is somewhat higher for in-cell reactivities in some positions. Also, there is a reactive spike at position 10 *in vitro*, but it is likely a spurious peak and not representative. (c) taR12 has a similar reactivity profile *in vitro* and in-cell. Nucleotides 4, 9, and 18-19 are somewhat higher in-cell while nucleotides 41, 44, 49, and some within 60-68 are higher *in vitro*.

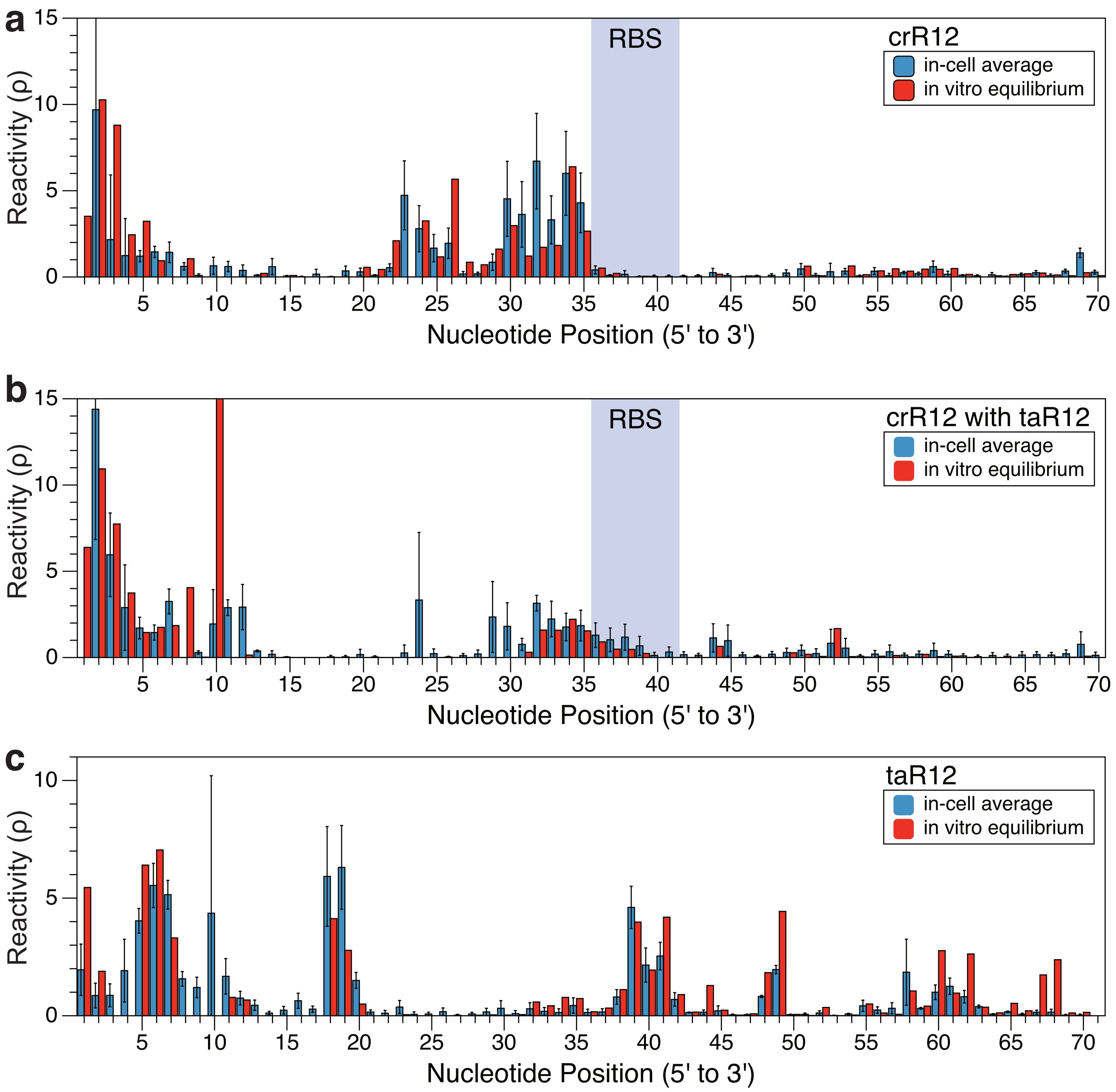

Supplementary Figure 23. In-cell vs. equilibrium *in vitro* refolding reactivity maps for RNA-IN/OUT. Bar charts comparing reactivities measured in-cell (RNA-IN S4 C24A A25C) and *in vitro* (RNA-IN S3 C24A A25C) for the RNA-IN double mutants and RNA-OUT A3. Error bars for the in-cell measurements represent one standard deviation. RNA-IN S3 C24A A25C was used in place of S4 C24A A25C in refolding experiments to ease *in vitro* transcription with T7 polymerase. (a) Few differences are observed for RNA-IN between in-cell (with control antisense plasmid) and *in vitro* equilibrium measurements. Nucleotides 6 and 7 are higher in-cell, while nucleotides around positions 20 and 25 are higher *in vitro*. (b) The differences between RNA-IN in-cell and *in vitro* are more pronounced when the cognate RNA-OUT is added. The reactivities around the RBS region are generally higher *in vitro*, while other nucleotides in RNA-IN are higher in-cell. (c) The reactivity pattern for RNA-OUT A3 matches well between in-cell and *in vitro* except for nucleotides in the 5' end, which appear more reactive *in vitro*.

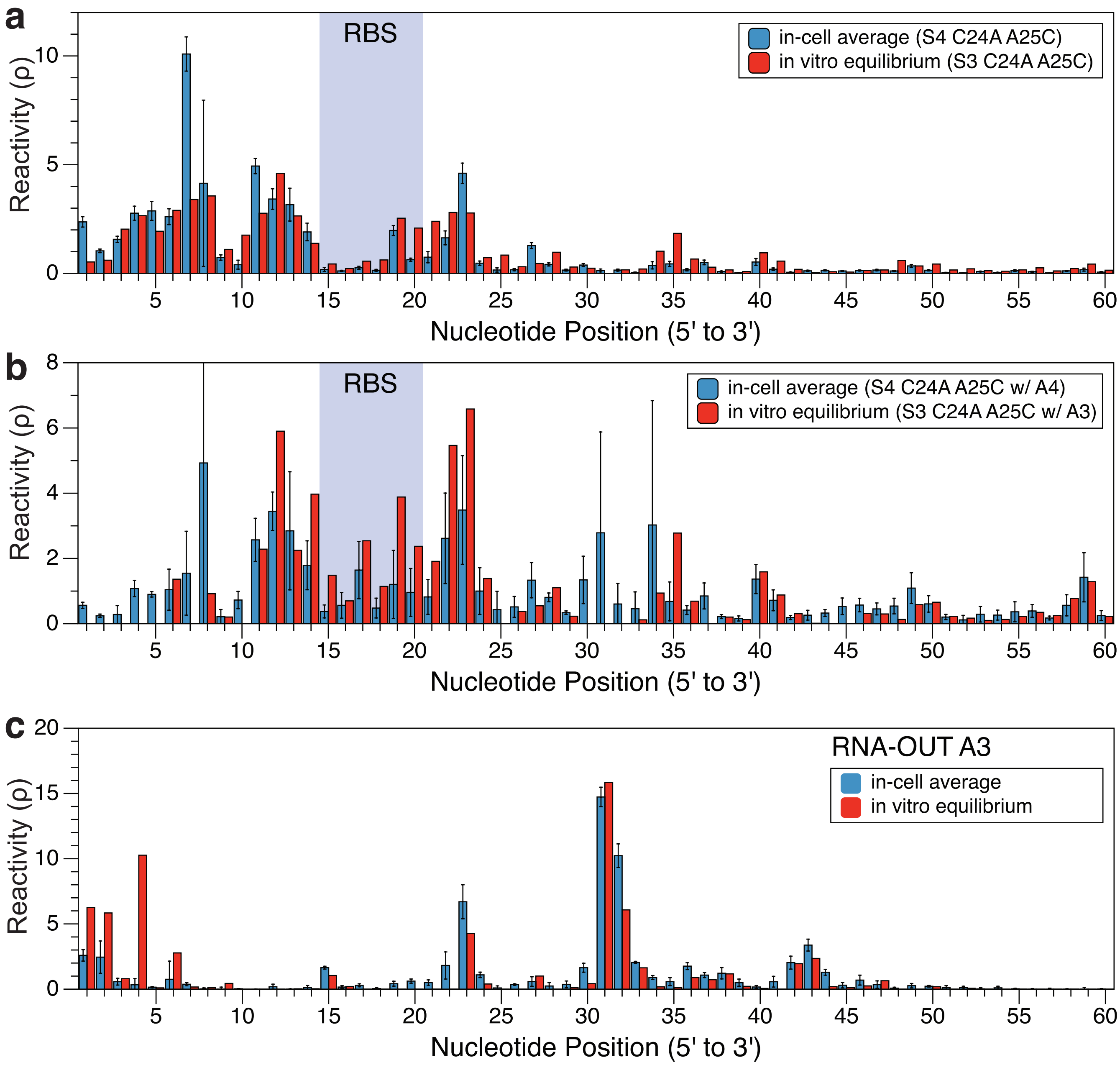

Supplementary Figure 24. In-cell vs. equilibrium *in vitro* refolding reactivity maps of 5S rRNA from *E. coli*. Reactivity maps comparing the 5S rRNA measured from in-cell and *in vitro* experiments. Error bars represent one standard deviation. *In vitro* data is taken from Loughrey, et al. (11) and can be found in the RMDB (12) with IDs: 5SRRNA_1M7_0001, 5SRRNA_1M7_0002, and 5SRRNA_1M7_0003. There are many reactivity differences between the in-cell and *in vitro* conditions. Most notably, nucleotides 35-38 have greatly increased reactivity in the cell, while nucleotides from position 44 to the 3' end all greatly decrease in the cell. In contrast, we observe that the reactivity clusters near positions 12 and 25 are similar between the *in vitro* and in-cell conditions. Few reactivity changes in those regions would be expected because those nucleotides are in loop regions of the 5S rRNA that face away from the ribosome into the solvent (Figure 5a, Supplementary Movie S1). The region around nucleotides 35-55 fits in a groove near the L5 protein (13, 14) and is likely remodeled as it fits into the ribosome, which could cause the differences in the reactivities in that region of the reactivity map.

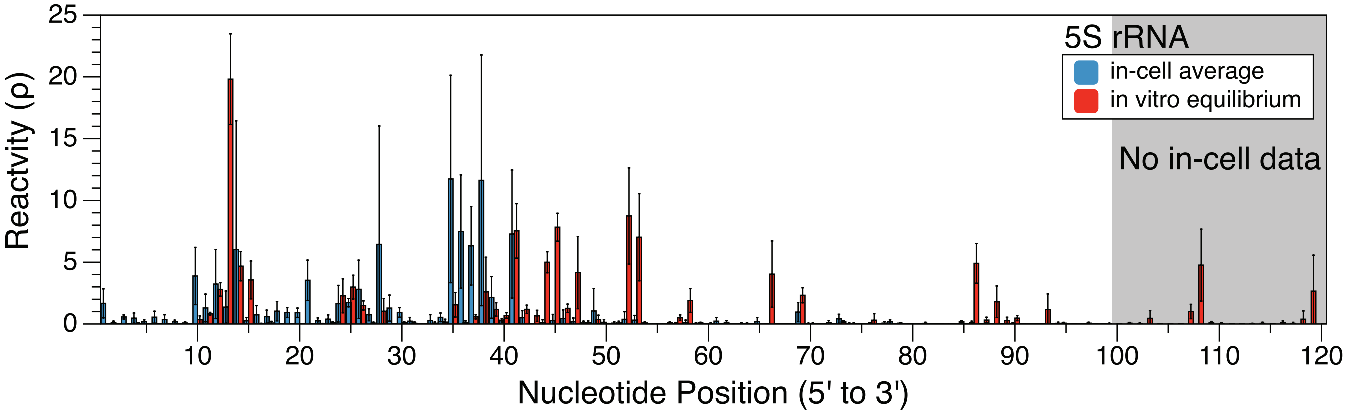

Supplementary Table 2. RNA sequences and plasmids used in this study. The sense RNAs (crRNAs and RNA-IN) are expressed before the superfolder GFP (SFGFP) sequence (1). The ribosome binding sites (RBS) within the regulator sequences and the sense RT primer binding site are near the 5' end of the SF-GFP sequence. The antisense RNAs (taRNAs and RNA-OUT) are terminated by a double terminator composed of the ECK120051404 terminator (2), which contains the antisense RT primer binding site, and the t500 terminator. See Supplementary Figure S1 for plasmid construction order. For the endogenously expressed RNAs the entire transcript is shown, with each specific RT priming site bolded and underlined.

Name	Sequence
btuB mRNA	GCCGGTCCTGTGAGTTAATAGGGAATCCAGTGCGAATCTGGAGCTGACGCGCAGCGGTAAGGAAAGGTGCGATGATTGCGTTATGCGGACA<br>CTGCCATTCGGTGGGAAGTCATCATCTCTTAGTATCTTAGATACCCCTCCAAGCCCGAAGACCTGCCGGCCAACGTCGCATCTGGTTCTCA<br>TCATCGCGTAATATTGATGAAACCTGCG**GCATCCTTCTTCTATTGTGGATGC**TTTACAATGATTAAAAAGCTTCGCTGCTGACGGCGTGT<br>TCCGTCACGGCATTTTCCGCTTGGGCACAGGATACCAGCCCGGATACTCTCGTCGTTACTGCTAACCGTTTTGAACAGCCGCGCAGCACTG<br>TGCTTGCACCAACCACCGTTGTGACCCGTCAGGATATCGACCGCTGGCAGTCGACCTCGGTCAATGATGTGCTGCGCCGTCTTCCGGGCGT<br>CGATATCACCCAAAACGGCGGTTCAGGTCAGCTCTCATCTATTTTTATTCGCGGTACAAATGCCAGTCATGTGTTGGTGTTAATTGATGGC<br>GTACGCCTGAATCTGGCGGGGGTGAGTGGTTCTGCCGACCTTAGCCAGTTCCCTATTGCGCTTGTCCAGCGTGTTGAATATATCCGTGGGC<br>CGCGCTCCGCTGTTTATGGTTCCGATGCAATAGGCGGGGTGGTGAATATCATCACGACGCGCGATGAACCCGGAACGGAAATTTCAGCAGG<br>GTGGGGAAGCAATAGTTATCAGAACTATGATGTCTCTACGCAGCAACAACTGGGGGATAAGACACGGGTAACGCTGTTGGGCGATTATGCC<br>CATACTCATGGTTATGATGTTGTTGCCTATGGTAATACCGGAACGCAAGCGCAGACAGATAACGATGGTTTTTTAAGTAAAACGCTTTATG<br>GCGCGCTGGAGCATAACTTTACTGATGCCTGGAGCGGCTTTGTGCGCGGCTATGGCTATGATAACCGTACCAATTATGACGCGTATTATTC<br>TCCCGGTTCACCGTTGCTCGATACCCGTAAACTCTATAGCCAAAGTTGGGACGCCGGGCTGCGCTATAACGGCGAACTGATTAAATCACAA<br>CTCATTACCAGCTATAGCCATAGCAAAGATTACAACTACGATCCCCATTATGGTCGTTATGATTCGTCGGCGACGCTCGATGAGATGAAGC<br>AATACACCGTCCAGTGGGCAAACAATGTCATCGTTGGTCACGGTAGTATTGGTGCGGGTGTCGACTGGCAGAAACAGACTACGACGCCGGG<br>TACAGGTTATGTTGAGGATGGATATGATCAACGTAATACCGGCATCTATCTGACCGGGCTGCAACAAGTCGGCGATTTTACCTTTGAAGGC<br>GCAGCACGCAGTGACGATAACTCACAGTTTGGTCGTCATGGAACCTGGCAAACCAGCGCCGGTTGGGAATTCATCGAAGGTTATCGCTTCA<br>TTGCTTCCTACGGGACATCTTATAAGGCACCAAATCTGGGGCAACTGTATGGCTTCTACGGAAATCCGAATCTGGACCCGGAGAAAAGCAA<br>ACAGTGGGAAGGCGCGTTTGAAGGCTTAACCGCTGGGGTGAACTGGCGTATTTCCGGATATCGTAACGATGTCAGTGACTTGATCGATTAT<br>GATGATCACACCCTGAAATATTACAACGAAGGGAAAGCGCGGATTAAGGGCGTCGAGGCGACCGCCAATTTTGATACCGGACCACTGACGC<br>ATACTGTGAGTTATGATTATGTCGATGCGCGCAATGCGATTACCGACACGCCGTTGTTACGCCGTGCTAAACAGCAGGTGAAATACCAGCT<br>CGACTGGCAGTTGTATGACTTCGACTGGGGTATTACTTATCAGTATTTAGGCACTCGCTATGATAAGGATTACTCATCTTATCCTTATCAA<br>ACCGTTAAAATGGGCGGTGTGAGCTTGTGGGATCTTGCGGTTGCGTATCCGGTCACCTCTCACCTGACAGTTCGTGGTAAAATAGCCAACC<br>TGTTCGACAAAGATTATGAGACAGTCTATGGCTACCAAACTGCAGGACGGGAATACACCTTGTCTGGCAGCTACACCTTCTGA
5S rRNA	TGCCTGGCGGCCGTAGCGCGGTGGTCCCACCTGACCCCATGCCGAACTCAGAAGTGAAACGCCGTAGCGCCGATGGTAGTGTGGGGTCTCC<br>CCATGCGAGAG**TAGGGAACTGCCAGGCAT**
RNase P	GAAGCTGACCAGACAGTCGCCGCTTCGTCGTCGTCCTCTTCGGGGGAGACGGGCGGAGGGGAGGAAAGTCCGGGCTCCATAGGGCAGGGTG<br>CCAGGTAACGCCTGGGGGGGAAACCCACGACCAGTGCAACAGAGAGCAAACCGCCGATGGCCCGCGCAAGCGGGATCAGGTAAGGGTGAAA<br>GGGTGCGGTAAGAGCGCACCGCGCGGCTGGTAACAGTCCGTGGCACGGTAAACTCCACCCGGAGCAAGG**CCAAATAGGGGTTCATAAGGTA**<br>**CGG**CCCGTACTGAACCCGGGTAGGCTGCTTGAGCCAGTGAGCGATTGCTGGCCTAGATGAATGACTGTCCACGACAGAACCCGGCTTATCG<br>GTCAGTTTCACCT
crR10	GAATTCTACCTATCTGCTCTTGAATTTGGGTATTAAAGAGGAGAAAGGTACC – SFGFP – TrrnB
crR12	GAATTCTACCATTCACCTCTTGGATTTGGGTATTAAAGAGGAGAAAGGTACC – SFGFP – TrrnB
taR10	ACACCCAAATTCATGAGCAGATTGGTAGTGGTGGTTAATGAAAATTAACTTACTACTACCTTTCTCTAGAG – ECK404 – t500
taR12	ACCCAAATCCAGGAGGTGATTGGTAGTGGTGGTTAATGAAAATTAACTTACTACTACCATATATCTCTAGAG – ECK404 – t500
RNA-IN S3	GGGAAAAATCAATAAGGAGACAACAAGATGTGCGAACTCGATGCT – SFGFP (w/o AUG) – Double terminator
RNA-IN S4	GCCAAAAATCAATAAGGAGACAACAAGATGTGCGAACTCGATGCT – SFGFP (w/o AUG) – Double terminator
RNA-IN S4 A25C	GCCAAAAATCAATAAGGAGACAACCAGATGTGCGAACTCGATGCT – SFGFP (w/o AUG) – Double terminator
RNA-IN S4 C24A	GCCAAAAATCAATAAGGAGACAAAAAGATGTGCGAACTCGATGCT – SFGFP (w/o AUG) – Double terminator
RNA-IN S4 C24A A25C	GCCAAAAATCAATAAGGAGACAAACAGATGTGCGAACTCGATGCT – SFGFP (w/o AUG) – Double terminator
RNA-OUT A3	TCGCACATCTTGTTGTCTGATTATTGATTTTTCCCGAAACCATTTGATCATATGACAAGATGTGTATG – ECK404 – t500

RNA-OUT A4	TCGCACATCTTGTTGTCTGATTATTGATTTTTGGCGAAACCATTTGATCATATGACAAGATGTGTATG – ECK404 – t500
Superfolder GFP (SFGFP)	ATGAGCAAAGGAGAAGAACTTTTCACTGGAGTTGTCCCAATTCTTGTTGAATTAGATGGTGATGTTAATGGGCACAAATTTTCTGTCCGTG<br>GAGAGGGTGAAGGTGATGCTACAAACGGAAAACTCACCCTTAAATTTATTTGCACTACTGGAAAACTACCTGTTCCGTGGCCAACACTTGT<br>CACTACTCTGACCTATGGTGTTCAATGCTTTTCCCGTTATCCGGATCACATGAAACGGCATGACTTTTTCAAGAGTGCCATGCCCGAAGGT<br>TATGTACAGGAACGCACTATATCTTTCAAAGATGACGGGACCTACAAGACGCGTGCTGAAGTCAAGTTTGAAGGTGATACCCTTGTTAATC<br>GTATCGAGTTAAAGGGTATTGATTTTAAAGAAGATGGAAACATTCTTGGACACAAACTCGAGTACAACTTTAACTCACACAATGTATACAT<br>CACGGCAGACAAACAAAAGAATGGAATCAAAGCTAACTTCAAAATTCGCCACAACGTTGAAGATGGTTCCGTTCAACTAGCAGACCATTAT<br>CAACAAAATACTCCAATTGGCGATGGCCCTGTCCTTTTACCAGACAACCATTACCTGTCGACACAATCTGTCCTTTCGAAAGATCCCAACG<br>AAAAGCGTGACCACATGGTCCTTCTTGAGTTTGTAACTGCTGCTGGGATTACACATGGCATGGATGAGCTCTACAAATAA
TrrnB operon fragment	GGATCTGAAGCTTGGGCCCGAACAAAAACTCATCTCAGAAGAGGATCTGAATAGCGCCGTCGACCATCATCATCATCATCATTGAGTTTAA<br>ACGGTCTCCAGCTTGGCTGTTTTGGCGGATGAGAGAAGATTTTCAGCCTGATACAGATTAAATCAGAACGCAGAAGCGGTCTGATAAAACA<br>GAATTTGCCTGGCGGCAGTAGCGCGGTGGTCCCACCTGACCCCATGCCGAACTCAGAAGTGAAACGCCGTAGCGCCGATGGTAGTGTGGGG<br>TCTCCCCATGCGAGAGTAGGGAACTGCCAGGCATCAAATAAAACGAAAGGCTCAGTCGAAAGACTGGGCCTTTCGTTTTATCTGTTGTTTG<br>TCGGTGAACTGGATCCTTACTCGAGTCTAGA
ECK1200-51404 (ECK404) terminator	CCTCTACCTGCTTCGGCCGATAAAGCCGACGATAATACTCC
t500 terminator	CAAAGCCCGCCGAAAGGCGGGCTTTTTTT
Double terminator	GGATCCAAACTCGAGTAAGGATCTCCAGGCATCAAATAAAACGAAAGGCTCAGTCGAAAGACTGGGCCTTTCGTTTTATCTGTTGTTTGTC<br>GGTGAACGCTCTCTACTAGAGTCACACTGGCTCACCTTCGGGTGGGCCTTTCTGCGTTTATACCTAGGGTACGGGTTTTGC
ColE1 ori fragment	GGATCCTTACTCGAGTCTAGACTGCAGGCTTCCTCGCTCACTGACTCGCTGCGCTCGGTCGTTCGGCTGCGGCGAGCGGTATCAGCTCACT<br>CAAAGGCGGTAATACGGTTATCCACAGAATCAGGGGATAACGCAGGAAAGAACATGTGAGCAAAAGGCCAGCAAAAGGCCAGGAACCGTAA<br>AAAGGCCGCGTTGCTGGCGTTTTTCCACAGGCTCCGCCCCCCTGACGAGCATCACAAAAATCGACGCTCAAGTCAGAGGTGGCGAAACCCG<br>ACAGGACTATAAAGATACCAGGCGTTTCCCCCTGGAAGCTCCCTCGTGCGCTCTCCTGTTCCGACCCTGCCGCTTACCGGATACCTGTCCG<br>CCTTTCTCCCTTCGGGAAGCGTGGCGCTTTCTCATAGCTCACGCTGTAGGTATCTCAGTTCGGTGTAGGTCGTTCGCTCCAAGCTGGGCTG<br>TGTGCACGAACCCCCCGTTCAGCCCGACCGCTGCGCCTTATCCGGTAACTATCGTCTTGAGTCCAACCCGGTAAGACACGACTTATCGCCA<br>CTGGCAGCAGCCACTGGTAACAGGATTAGCAGAGCGAGGTATGTAGGCGGTGCTACAGAGTTCTTGAAGTGGTGGCCTAACTACGGCTACA<br>CTAGAAGAACAGTATTTGGTATCTGCGCTCTGCTGAAGCCAGTTACCTTCGGAAAAAGAGTTGGTAGCTCTTGATCCGGCAAACAAACCAC<br>CGCTGGTAGCGGTGGTTTTTTTGTTTGCAAGCAGCAGATTACGCGCAGAAAAAAAGGATCTCAAGAAGATCCTTTGATCTTTTCTACGGGG<br>TCTGACGCTCAGTGGAACGAAAACTCACGTTAAGGGATTTTGGTCATGA
p15A ori fragment	GGTGAGAATCCAAGCCTCCGATCAACGTCTCATTTTCGCCAAAAGTTGGCCCAGGGCTTCCCGGTATCAACAGGGACACCAGGATTTATTT<br>ATTCTGCGAAGTGATCTTCCGTCACAGGTATTTATTCGGCGCAAAGTGCGTCGGGTGATGCTGCCAACTTACTGATTTAGTGTATGATGGT<br>GTTTTTGAGGTGCTCCAGTGGCTTCTGTTTCTATCAGCTGTCCCTCCTGTTCAGCTACTGACGGGGTGGTGCGTAACGGCAAAAGCACCGC<br>CGGACATCAGCGCTAGCGGAGTGTATACTGGCTTACTATGTTGGCACTGATGAGGGTGTCAGTGAAGTGCTTCATGTGGCAGGAGAAAAAA<br>GGCTGCACCGGTGCGTCAGCAGAATATGTGATACAGGATATATTCCGCTTCCTCGCTCACTGACTCGCTACGCTCGGTCGTTCGACTGCGG<br>CGAGCGGAAATGGCTTACGAACGGGGCGGAGATTTCCTGGAAGATGCCAGGAAGATACTTAACAGGGAAGTGAGAGGGCCGCGGCAAAGCC<br>GTTTTTCCATAGGCTCCGCCCCCCTGACAAGCATCACGAAATCTGACGCTCAAATCAGTGGTGGCGAAACCCGACAGGACTATAAAGATAC<br>CAGGCGTTTCCCCCTGGCGGCTCCCTCGTGCGCTCTCCTGTTCCTGCCTTTCGGTTTACCGGTGTCATTCCGCTGTTATGGCCGCGTTTGT<br>CTCATTCCACGCCTGACACTCAGTTCCGGGTAGGCAGTTCGCTCCAAGCTGGACTGTATGCACGAACCCCCCGTTCAGTCCGACCGCTGCG<br>CCTTATCCGGTAACTATCGTCTTGAGTCCAACCCGGAAAGACATGCAAAAGCACCACTGGCAGCAGCCACTGGTAATTGATTTAGAGGAGT<br>TAGTCTTGAAGTCATGCGCCGGTTAAGGCTAAACTGAAAGGACAAGTTTTGGTGACTGCGCTCCTCCAAGCCAGTTACCTCGGTTCAAAGA<br>GTTGGTAGCTCAGAGAACCTTCGAAAAACCGCCCTGCAAGGCGGTTTTTTCGTTTTCAGAGCAAGAGATTACGCGCAGACCAAAACGATCT<br>CAAGAAGATCATCTTATTAATCAGATAAAATATTTCTAGATTTCAGTGCAATTTATCTCTTCAAATGTAGCACCTGAAGTCAGCCCCATAC<br>GATATAAGTTGTAATTCTCATGTTTGACAGCTTATCATCGATAAGCTTCCGATGGCGCGCCGAGAGGCTTTACACTTTATGCTTCCGGCT
Amp^R fragment	GATTATCAAAAAGGATCTTCACCTAGATCCTTTTAAATTAAAAATGAAGTTTTAAATCAATCTAAAGTATATATGAGTAAACTTGGTCTGA<br>CAGTTACCAATGCTTAATCAGTGAGGCACCTATCTCAGCGATCTGTCTATTTCGTTCATCCATAGTTGCCTGACTCCCCGTCGTGTAGATA<br>ACTACGATACGGGAGGGCTTACCATCTGGCCCCAGTGCTGCAATGATACCGCGAGACCCACGCTCACCGGCTCCAGATTTATCAGCAATAA<br>ACCAGCCAGCCGGAAGGGCCGAGCGCAGAAGTGGTCCTGCAACTTTATCCGCCTCCATCCAGTCTATTAATTGTTGCCGGGAAGCTAGAGT<br>AAGTAGTTCGCCAGTTAATAGTTTGCGCAACGTTGTTGCCATTGCTACAGGCATCGTGGTGTCACGCTCGTCGTTTGGTATGGCTTCATTC<br>AGCTCCGGTTCCCAACGATCAAGGCGAGTTACATGATCCCCCATGTTGTGCAAAAAAGCGGTTAGCTCCTTCGGTCCTCCGATCGTTGTCA<br>GAAGTAAGTTGGCCGCAGTGTTATCACTCATGGTTATGGCAGCACTGCATAATTCTCTTACTGTCATGCCATCCGTAAGATGCTTTTCTGT<br>GACTGGTGAGTACTCAACCAAGTCATTCTGAGAATAGTGTATGCGGCGACCGAGTTGCTCTTGCCCGGCGTCAATACGGGATAATACCGCG<br>CCACATAGCAGAACTTTAAAAGTGCTCATCATTGGAAAACGTTCTTCGGGGCGAAAACTCTCAAGGATCTTACCGCTGTTGAGATCCAGTT<br>CGATGTAACCCACTCGTGCACCCAACTGATCTTCAGCATCTTTTACTTTCACCAGCGTTTCTGGGTGAGCAAAAACAGGAAGGCAAAATGC<br>CGCAAAAAAGGGAATAAGGGCGACACGGAAATGTTGAATACTCATACTCTTCCTTTTTCAATATTATTGAAGCATTTATCAGGGTTATTGT<br>CTCATGAGCGGATACATATTTGAATGTATTTAGAAAAATAAACAAATAGGGGTTCCGCGCACATTTCCCCGAAAAGTGCCACCTGACGTCT<br>AAGAAACCATTATTATCATGACATTAACCTATAAAAATAGGCGTATCACGAGGCAGAATTTCAGATAAAAAAAATCCTTAGCTTTCGCTAA<br>GGATGATTTCTG
Cm^R fragment	CTGCAGTTGATCGGGCACGTAAGAGGTTCCAACTTTCACCATAATGAAATAAGATCACTACCGGGCGTATTTTTTGAGTTATCGAGATTTT<br>CAGGAGCTAAGGAAGCTAAAATGGAGAAAAAAATCACTGGATATACCACCGTTGATATATCCCAATGGCATCGTAAAGAACATTTTGAGGC<br>ATTTCAGTCAGTTGCTCAATGTACCTATAACCAGACCGTTCAGCTGGATATTACGGCCTTTTTAAAGACCGTAAAGAAAAATAAGCACAAG<br>TTTTATCCGGCCTTTATTCACATTCTTGCCCGCCTGATGAATGCTCATCCGGAATTTCGTATGGCAATGAAAGACGGTGAGCTGGTGATAT<br>GGGATAGTGTTCACCCTTGTTACACCGTTTTCCATGAGCAAACTGAAACGTTTTCATCGCTCTGGAGTGAATACCACGACGATTTCCGGCA<br>GTTTCTACACATATATTCGCAAGATGTGGCGTGTTACGGTGAAAACCTGGCCTATTTCCCTAAAGGGTTTATTGAGAATATGTTTTTCGTC<br>TCAGCCAATCCCTGGGTGAGTTTCACCAGTTTTGATTTAAACGTGGCCAATATGGACAACTTCTTCGCCCCCGTTTTCACCATGGGCAAAT<br>ATTATACGCAAGGCGACAAGGTGCTGATGCCGCTGGCGATTCAGGTTCATCATGCCGTTTGTGATGGCTTCCATGTCGGCAGAATGCTTAA<br>TGAATTACAACAGTACTGCGATGAGTGGCAGGGCGGGGCGTAATTTGATATCGAGCTCGCTTGGACTCCTGTTGATAGATCCAGTAATGAC<br>CTCAGAACTCCATCTGGATTTGTTCAGAACGCTCGGTTGCCGCCGGGCGTTTTTTATT

J23119 promoter variants	GAATTC(TAAAGATC/GAA)TTTGACAGCTAGCTCAGTCCTAGGTATAAT(ACTAGT/GAATTC)<br>(riboregulators/RNA-OUT-RNA-IN) (riboregulators/RNA-IN-RNA-OUT)
Example plasmid (crR10)	GAATTCTAAAGATCTTTGACAGCTAGCTCAGTCCTAGGTATAATACTAGTGAATTCTACCTATCTGCTCTTGAATTTGGGTATTAAAGAGG<br>AGAAAGGTACCATGAGCAAAGGAGAAGAACTTTTCACTGGAGTTGTCCCAATTCTTGTTGAATTAGATGGTGATGTTAATGGGCACAAATT<br>TTCTGTCCGTGGAGAGGGTGAAGGTGATGCTACAAACGGAAAACTCACCCTTAAATTTATTTGCACTACTGGAAAACTACCTGTTCCGTGG<br>CCAACACTTGTCACTACTCTGACCTATGGTGTTCAATGCTTTTCCCGTTATCCGGATCACATGAAACGGCATGACTTTTTCAAGAGTGCCA<br>TGCCCGAAGGTTATGTACAGGAACGCACTATATCTTTCAAAGATGACGGGACCTACAAGACGCGTGCTGAAGTCAAGTTTGAAGGTGATAC<br>CCTTGTTAATCGTATCGAGTTAAAGGGTATTGATTTTAAAGAAGATGGAAACATTCTTGGACACAAACTCGAGTACAACTTTAACTCACAC<br>AATGTATACATCACGGCAGACAAACAAAAGAATGGAATCAAAGCTAACTTCAAAATTCGCCACAACGTTGAAGATGGTTCCGTTCAACTAG<br>CAGACCATTATCAACAAAATACTCCAATTGGCGATGGCCCTGTCCTTTTACCAGACAACCATTACCTGTCGACACAATCTGTCCTTTCGAA<br>AGATCCCAACGAAAAGCGTGACCACATGGTCCTTCTTGAGTTTGTAACTGCTGCTGGGATTACACATGGCATGGATGAGCTCTACAAATAA<br>GGATCTGAAGCTTGGGCCCGAACAAAAACTCATCTCAGAAGAGGATCTGAATAGCGCCGTCGACCATCATCATCATCATCATTGAGTTTAA<br>ACGGTCTCCAGCTTGGCTGTTTTGGCGGATGAGAGAAGATTTTCAGCCTGATACAGATTAAATCAGAACGCAGAAGCGGTCTGATAAAACA<br>GAATTTGCCTGGCGGCAGTAGCGCGGTGGTCCCACCTGACCCCATGCCGAACTCAGAAGTGAAACGCCGTAGCGCCGATGGTAGTGTGGGG<br>TCTCCCCATGCGAGAGTAGGGAACTGCCAGGCATCAAATAAAACGAAAGGCTCAGTCGAAAGACTGGGCCTTTCGTTTTATCTGTTGTTTG<br>TCGGTGAACTGGATCCTTACTCGAGTCTAGACTGCAGTTGATCGGGCACGTAAGAGGTTCCAACTTTCACCATAATGAAATAAGATCACTA<br>CCGGGCGTATTTTTTGAGTTATCGAGATTTTCAGGAGCTAAGGAAGCTAAAATGGAGAAAAAAATCACTGGATATACCACCGTTGATATAT<br>CCCAATGGCATCGTAAAGAACATTTTGAGGCATTTCAGTCAGTTGCTCAATGTACCTATAACCAGACCGTTCAGCTGGATATTACGGCCTT<br>TTTAAAGACCGTAAAGAAAAATAAGCACAAGTTTTATCCGGCCTTTATTCACATTCTTGCCCGCCTGATGAATGCTCATCCGGAATTTCGT<br>ATGGCAATGAAAGACGGTGAGCTGGTGATATGGGATAGTGTTCACCCTTGTTACACCGTTTTCCATGAGCAAACTGAAACGTTTTCATCGC<br>TCTGGAGTGAATACCACGACGATTTCCGGCAGTTTCTACACATATATTCGCAAGATGTGGCGTGTTACGGTGAAAACCTGGCCTATTTCCC<br>TAAAGGGTTTATTGAGAATATGTTTTTCGTCTCAGCCAATCCCTGGGTGAGTTTCACCAGTTTTGATTTAAACGTGGCCAATATGGACAAC<br>TTCTTCGCCCCCGTTTTCACCATGGGCAAATATTATACGCAAGGCGACAAGGTGCTGATGCCGCTGGCGATTCAGGTTCATCATGCCGTTT<br>GTGATGGCTTCCATGTCGGCAGAATGCTTAATGAATTACAACAGTACTGCGATGAGTGGCAGGGCGGGGCGTAATTTGATATCGAGCTCGC<br>TTGGACTCCTGTTGATAGATCCAGTAATGACCTCAGAACTCCATCTGGATTTGTTCAGAACGCTCGGTTGCCGCCGGGCGTTTTTTATTGG<br>TGAGAATCCAAGCCTCCGATCAACGTCTCATTTTCGCCAAAAGTTGGCCCAGGGCTTCCCGGTATCAACAGGGACACCAGGATTTATTTAT<br>TCTGCGAAGTGATCTTCCGTCACAGGTATTTATTCGGCGCAAAGTGCGTCGGGTGATGCTGCCAACTTACTGATTTAGTGTATGATGGTGT<br>TTTTGAGGTGCTCCAGTGGCTTCTGTTTCTATCAGCTGTCCCTCCTGTTCAGCTACTGACGGGGTGGTGCGTAACGGCAAAAGCACCGCCG<br>GACATCAGCGCTAGCGGAGTGTATACTGGCTTACTATGTTGGCACTGATGAGGGTGTCAGTGAAGTGCTTCATGTGGCAGGAGAAAAAAGG<br>CTGCACCGGTGCGTCAGCAGAATATGTGATACAGGATATATTCCGCTTCCTCGCTCACTGACTCGCTACGCTCGGTCGTTCGACTGCGGCG<br>AGCGGAAATGGCTTACGAACGGGGCGGAGATTTCCTGGAAGATGCCAGGAAGATACTTAACAGGGAAGTGAGAGGGCCGCGGCAAAGCCGT<br>TTTTCCATAGGCTCCGCCCCCCTGACAAGCATCACGAAATCTGACGCTCAAATCAGTGGTGGCGAAACCCGACAGGACTATAAAGATACCA<br>GGCGTTTCCCCCTGGCGGCTCCCTCGTGCGCTCTCCTGTTCCTGCCTTTCGGTTTACCGGTGTCATTCCGCTGTTATGGCCGCGTTTGTCT<br>CATTCCACGCCTGACACTCAGTTCCGGGTAGGCAGTTCGCTCCAAGCTGGACTGTATGCACGAACCCCCCGTTCAGTCCGACCGCTGCGCC<br>TTATCCGGTAACTATCGTCTTGAGTCCAACCCGGAAAGACATGCAAAAGCACCACTGGCAGCAGCCACTGGTAATTGATTTAGAGGAGTTA<br>GTCTTGAAGTCATGCGCCGGTTAAGGCTAAACTGAAAGGACAAGTTTTGGTGACTGCGCTCCTCCAAGCCAGTTACCTCGGTTCAAAGAGT<br>TGGTAGCTCAGAGAACCTTCGAAAAACCGCCCTGCAAGGCGGTTTTTTCGTTTTCAGAGCAAGAGATTACGCGCAGACCAAAACGATCTCA<br>AGAAGATCATCTTATTAATCAGATAAAATATTTCTAGATTTCAGTGCAATTTATCTCTTCAAATGTAGCACCTGAAGTCAGCCCCATACGA<br>TATAAGTTGTAATTCTCATGTTTGACAGCTTATCATCGATAAGCTTCCGATGGCGCGCCGAGAGGCTTTACACTTTATGCTTCCGGCT

Supplementary Table 3. Oligonucleotides used in this study. Below is a table of oligonucleotides used during the in-cell SHAPE-Seq experiments with the platform described in Supplementary Figure S1 and the endogenously expressed RNAs. Primers used during platform construction are not included. This table is mainly meant to serve as a reference for the Supplementary Methods. Abbreviations within primer sequences are as follows: '/5Biosg/' is a 5' biotin moiety, '/5Phos/' is a 5' monophosphate group, '/3SpC3/' is a 3' 3-carbon spacer group, VIC and NED are fluorophores (ABI), and asterisks indicate a phosphorothioate backbone modification. These abbreviations were used for compatibility with the Integrated DNA Technologies ordering notation.

**Reverse Transcription**		
ECK120051404 Terminator (ECK404)	/5Biosg/TTTATCGGCCGAAGCAGGTAG	A
Super Folder GFP (SFGFP)	/5Biosg/CAACAAGAATTGGGACAACTCCAGTG	B
5S rRNA (E. coli)	ATGCCTGGCAGTTCCCTA	C
RNase P specificity region (E. coli)	CCGTACCTTATGAACCCCTATTTGG	D
btuB riboswitch 5' UTR (E. coli)	GCATCCACAATAGAAGAAGGATGC	E
**Adapter Ligation**		
A_adapter_b (A_b) (ssDNA adapter)	/5Phos/AGATCGGAAGAGCACACGTCTGAACTCCAGTCAC/3SpC3/	F
**Fluorescent Quality Analysis**		
Reverse QA primer (+)	VIC-GTGACTGGAGTTCAGACGTGTGCTC	G
Reverse QA primer (-)	NED-GTGACTGGAGTTCAGACGTGTGCTC	H
**Primers for Building dsDNA Libraries**		
PE_forward[†]	AATGATACGGCGACCACCGAGATCTACACTCTTTCCCTACACGACGCTCTTCCGATCT	I
ECK404 (+) selection primer (forward)	CTTTCCCTACACGACGCTCTTCCGATCTRRRYTTTATCGGCCGAAGCAGGTAgA*G*G*C	J
ECK404 (-) selection primer (forward)	CTTTCCCTACACGACGCTCTTCCGATCTYYYRtTTATCGGCCGAAGCAGGTAgA*G*G*C	K
SF-GFP (+) selection primer (forward)	CTTTCCCTACACGACGCTCTTCCGATCTRRRYCAACAAGAATTGGGACAACTCCAGT*G*A*A*A*A*G	L
SF-GFP (-) selection primer (forward)	CTTTCCCTACACGACGCTCTTCCGATCTYYYRCAACAAGAATTGGGACAACTCCAGT*G*A*A*A*A*G	M
5S rRNA (+) selection primer (forward)	CTTTCCCTACACGACGCTCTTCCGATCTRRRYATGCCTGGCAGTTCCCTA*C*T*C	N
5S rRNA (-) selection primer (forward)	CTTTCCCTACACGACGCTCTTCCGATCTYYYRATGCCTGGCAGTTCCCTA*C*T*C	O
RNase P (+) selection primer (forward)	CTTTCCCTACACGACGCTCTTCCGATCTRRRYCCGTACCTTATGAACCCCTATTTGG*C*C*T	P
RNase P (-) selection primer (forward)	CTTTCCCTACACGACGCTCTTCCGATCTYYYRCCGTACCTTATGAACCCCTATTTGG*C*C*T	Q
btuB (+) selection primer (forward)	CTTTCCCTACACGACGCTCTTCCGATCTRRRYGCATCCACAATAGAAGAAGGATGC*C*G*C*A	R
btuB (-) selection primer (forward)	CTTTCCCTACACGACGCTCTTCCGATCTYYYRGCATCCACAATAGAAGAAGGATGC*C*G*C*A	S
**Illumina Multiplexing Primers**		
Illumina Index #1[†]	CAAGCAGAAGACGGCATACGAGATCGTGATGTGACTGGAGTTCAGACGTGTGCTC	T
Illumina Index #2[†]	CAAGCAGAAGACGGCATACGAGATACATCGGTGACTGGAGTTCAGACGTGTGCTC	U
Illumina Index #3[†]	CAAGCAGAAGACGGCATACGAGATGCCTAAGTGACTGGAGTTCAGACGTGTGCTC	V

Illumina Index #4†	CAAGCAGAAGACGGCATACGAGATTGGTCAGTGACTGGAGTTCAGACGTGTGCTC	W
Illumina Index #5†	CAAGCAGAAGACGGCATACGAGATCACTGTGTGACTGGAGTTCAGACGTGTGCTC	X
Illumina Index #6†	CAAGCAGAAGACGGCATACGAGATATTGGCGTGACTGGAGTTCAGACGTGTGCTC	Y
Illumina Index #7†	CAAGCAGAAGACGGCATACGAGATGATCTGGTGACTGGAGTTCAGACGTGTGCTC	Z
Illumina Index #8†	CAAGCAGAAGACGGCATACGAGATTCAAGTGTGACTGGAGTTCAGACGTGTGCTC	AA
Illumina Index #9†	CAAGCAGAAGACGGCATACGAGATCTGATCGTGACTGGAGTTCAGACGTGTGCTC	AB
Illumina Index #10†	CAAGCAGAAGACGGCATACGAGATAAGCTAGTGACTGGAGTTCAGACGTGTGCTC	AC
Illumina Index #11†	CAAGCAGAAGACGGCATACGAGATGTAGCCGTGACTGGAGTTCAGACGTGTGCTC	AD
Illumina Index #12†	CAAGCAGAAGACGGCATACGAGATTACAAGGTGACTGGAGTTCAGACGTGTGCTC	AE
Illumina Index #13†	CAAGCAGAAGACGGCATACGAGATTTGACTGTGACTGGAGTTCAGACGTGTGCTC	AF
Illumina Index #14†	CAAGCAGAAGACGGCATACGAGATGGAACTGTGACTGGAGTTCAGACGTGTGCTC	AG
Illumina Index #15†	CAAGCAGAAGACGGCATACGAGATTGACATGTGACTGGAGTTCAGACGTGTGCTC	AH
Illumina Index #16†	CAAGCAGAAGACGGCATACGAGATGGACGGGTGACTGGAGTTCAGACGTGTGCTC	AI
Illumina Index #18†	CAAGCAGAAGACGGCATACGAGATGCGGACGTGACTGGAGTTCAGACGTGTGCTC	AJ
Illumina Index #19†	CAAGCAGAAGACGGCATACGAGATTTTCACGTGACTGGAGTTCAGACGTGTGCTC	AK
Illumina Index #20†	CAAGCAGAAGACGGCATACGAGATGGCCACGTGACTGGAGTTCAGACGTGTGCTC	AL
Illumina Index #21†	CAAGCAGAAGACGGCATACGAGATCGAAACGTGACTGGAGTTCAGACGTGTGCTC	AM
Illumina Index #22†	CAAGCAGAAGACGGCATACGAGATCGTACGGTGACTGGAGTTCAGACGTGTGCTC	AN
Illumina Index #23†	CAAGCAGAAGACGGCATACGAGATCCACTCGTGACTGGAGTTCAGACGTGTGCTC	AO
Illumina Index #25†	CAAGCAGAAGACGGCATACGAGATATCAGTGTGACTGGAGTTCAGACGTGTGCTC	AP
Illumina Index #27†	CAAGCAGAAGACGGCATACGAGATAGGAATGTGACTGGAGTTCAGACGTGTGCTC	AQ

†Oligonucleotide sequences © 2007-2013 Illumina, Inc. All rights reserved.

Supplementary Equations 1-4. Normalization of reactivities and constrained structure prediction. The Spats software package (http://spats.sourceforge.net/) uses input sequencing reads (11, 15) to determine the θ_i value for each nucleotide, i, where θ_i represents the fraction of modifications occurring at position i in an RNA of length L (3). However, because θ_i is dependent on L, we introduced a new normalization method in Loughrey, et al. (11) to convert θ_i to ρ_i, which is the normalized reactivity intensity for a nucleotide i in an RNA of length L (which does not include the RT priming site), where $\langle\rho_i\rangle$ = 1. A brief explanation of the normalization method is presented below:

First, determine L, the length of the RNA region under study that does not include the bases specific to the RNA of interest in the selection primer. These bases constitute the RT priming site and the extension into the cDNA that provides selection against the dimer side products. For example, the sequence GCCTCTACCTGCTTCGGCCGATAAA should be excluded from the end of libraries that are generated from the ECK120051404 RT priming site. Next, determine a normalization constant, n, such that all of the θ_i values in L sum to 1 (Eq. 1). These steps are necessary because proper alignment of the sequencing reads to the RNA target requires the inclusion of the RT priming site sequence, since it is read by the Illumina platform. However, the RT priming site and the selection primer extension into the cDNA contain no structural information although they are automatically included in the calculation of θ_i by Spats. Thus, subtle differences in the (+) and (-) samples can cause nucleotides in the RT priming site to appear reactive, although they are simply an artifact of the data processing steps and contain no structural information. To exclude these spurious nucleotides from the original calculation of θ_i generated by Spats, n is calculated as:

$$n = \sum_{i=1}^{L} \theta_i \tag{1}$$

where L is the length of the RNA exclusive of the bases specific to the RNA of interest in the selection primer used to generate the dsDNA libraries. Here, an index of 1 refers to the 5' end of the RNA. Next, θ_i is converted to ρ_i by multiplying by the length, L, and dividing by the normalization factor (Eq. 2) to set the total average of ρ to 1 (Eq. 3).

$$\rho_i = \frac{\theta_i \cdot L}{n} \tag{2}$$

$$\bar{\rho} = \frac{\sum_{i=1}^{L} \rho_i}{L} = \frac{\sum_{i=1}^{L} \frac{\theta_i \cdot L}{n}}{L} = \frac{1}{n} \sum_{i=1}^{L} \theta_i = \frac{\sum_{i=1}^{L} \theta_i}{\sum_{i=1}^{L} \theta_i} = 1 \tag{3}$$

The normalized ρ_i values can be used to constrain the RNAstructure (11, 16) secondary structure predication program using a pseudo free energy term, ΔG (Eq. 4). Slope and intercept values were fit in Loughrey, et al. (11) to be m = 1.1, b = -0.3, which are the values used in this work.

$$\Delta G = m \cdot \ln(\rho_i + 1) + b \tag{4}$$

Supplementary Table 4. RMDB data deposition table. SHAPE-Seq reactivity spectra generated in this work are freely available from the RNA Mapping Database (RMDB) (http://rmdb.stanford.edu/repository/) (12), accessible using the RMDB ID numbers indicated in the table below. Any other reactivity data from this work can be provided upon request.

RMDB ID	Contents	Figure(s) used in
CRR10_1M7_0001	Triplicate data of crR10 co-expressed with the antisense control plasmid	3, S5, S6, S8, S10
CRR10_1M7_0002	Triplicate data of crR10 co-expressed with taR10	3, S8, S10
CRR12_1M7_0001	Triplicate data of crR12 co-expressed with the antisense control plasmid	2, 3, S5, S10, S22
CRR12_1M7_0002	Triplicate data of crR12 co-expressed with taR12	3, S10, S22
TAR10_1M7_0001	Triplicate data of taR10 expressed alone	S5, S6, S9
TAR12_1M7_0001	Triplicate data of taR12 expressed alone	2, S5, S9, S22
RNAINS3_1M7_0001	Triplicate data of RNA-IN S3 expressed alone	S12, S15
RNAINS4_1M7_0001	Triplicate data of RNA-IN S4 expressed alone	4, S14
INS4DBL_1M7_0001	Triplicate data of RNA-IN S4 C24A A25C double mutant co-expressed with the antisense control plasmid	4, S18, S23
INS4DBL_1M7_0002	Triplicate data of RNA-IN S4 C24A A25C double mutant co-expressed with RNA-OUT A4	4, S18, S23
RNAOUT3_1M7_0001	Triplicate data of RNA-OUT A3 expressed alone	S12, S23
RNAOUT4_1M7_0001	Triplicate data of RNA-OUT A4 expressed alone	4, S19
5SRRNA_1M7_0007	Triplicate data of endogenous *E. coli* 5S rRNA	5, S21, S24, SI Movie 1
BTUBR_1M7_0001	Five replicates of endogenous *E. coli* btuB riboswitch leader sequence	5, S21
RNASEP_1M7_0001	Triplicate data of endogenous *E. coli* RNase P specificity region	5, S21

Supplementary Methods. Detailed in-cell SHAPE-Seq protocol.

MATERIALS

- *E. coli* strain NEBTurbo (cloning strain)
- *E. coli* strain TG1 (experimental strain)
- Sense and antisense platform vectors – See Supplementary Figure S1
- Oligonucleotides, see Supplementary Table 3
- Cloning Reagents
    - 1.33X Gibson Assembly master mix (See below) (17)
    - Phusion High-Fidelity PCR kit (NEB, cat. no. E0553S)
    - Restriction enzyme DpnI (NEB, cat. no. R0176S)
    - T4 DNA ligase (NEB, cat. no. M0202S)
    - QIAprep spin Miniprep kits (Qiagen, cat. no. 27104)
    - QIAquick PCR purification kit (Qiagen cat. no. 28104)
    - Qiagen plasmid mini kit (Qiagen cat. no. 12123)
    - LB medium (Life Technologies, cat. no. 12795-084)
    - LB agar (BD, cat. no. 214530)
    - 5X KCM solution (0.5 M KCl, 0.15 M $CaCl_2$, 0.25 $MgCl_2$)
    - 2-YT medium (Invitrogen, cat. no. 22712-020)
    - Agarose gel electrophoresis reagents
    - Carbenicillin (Sigma-Aldrich, cat. no. C3416)
    - Chloramphenicol (Sigma-Aldrich, cat. no. C1919)
- 1-methyl-7-nitroisatoic anhydride – see (18)
- Anhydrous dimethyl sulfoxide (Sigma-Aldrich, cat. no. D8418)
- PBS solution (pH 7.4)
- Kanamycin sulfate (Sigma-Aldrich, cat. no. K1377)
- TRIzol Max Bacterial Enchancement Reagent (Ambion, cat. no. 16096-040)
- TRIzol reagent (Ambion, cat. no. 15596-026)
- Chloroform (Sigma-Aldrich, cat. no. 372978)
- Isopropyl alcohol (Sigma-Aldrich, cat. no. I9516)
- Ethanol (Sigma-Aldrich, cat. no. E7023)
- 10 mM dNTPs (NEB, cat no. N0447S)
- 0.1 M dithiothreitol (DTT) (Sigma-Aldrich, cat. no. D0632)
- Super Script III (SSIII) reverse transcriptase (Invitrogen, cat. no. 18080093)
- 5X SSIII First Strand Buffer (supplied with SSIII)
- Sodium Hydroxide (Sigma-Aldrich, cat. no. S8045)
- Hydrochloric Acid (Sigma-Aldrich, cat. no. H1758)
- CircLigase ssDNA Ligase, 10x CircLigase reaction buffer, 1mM ATP, and 50mM $MnCl_2$ (Epicentre, cat. no. CL4111K)
- 20 mg/mL glycogen (Invitrogen, cat. no. 10814-010)
- 3.0 M NaOAc (pH 5.5)
- Agencourt AMPure XP beads (Beckman Coulter, cat. no. A63880)
- TE Buffer (10 mM Tris-HCl, 1 mM EDTA, pH 7.5)
- GeneScan 500 LIZ standard (Applied Biosystems, cat. no. 4322682)
- Deionized formamide (Ambion, cat. no. AM9342)
- ExonucleaseI (ExoI) (NEB, cat. no. M0293S)

- Qubit dsDNA High Sensitivity Kit (Invitrogen, cat. no. Q32854)

EQUIPMENT (exact items used in this study in parentheses)
- 100 mm x 15 mm Petri dishes
- 0.7 mL, 1.5 mL microcentrifuge tubes
- QIAquick purification columns (Qiagen) or similar silica purification columns
- Thin-walled PCR tubes
- Optically clear 96-well reaction plates (Applied Biosystems, cat. no. N8010560)
- Clear bottom, black-sided 96-well plates (Corning, cat. no. 3631)
- 2 mL 96-well culture block (Corning, cat. no. 29445-164)
- Breathable adhesive cover for 96-well culture blocks (Sigma-Aldrich, cat. no. Z763624)
- Refrigerated microcentrifuge (to 4 °C)
- 96-well capable fluorescent spectrophotometer
- Thermal cycler
- Agarose gel electrophoresis setup
- Dry heating block
- Magnetic 96-well plate stand
- Shaking Incubator (96 deep-well compatible)
- Qubit 2.0 Fluorometer (Invitrogen, cat. no. Q32866)
- Unix, Linux or Mac OSX system for data analysis

REAGENT SETUP

1X Kanamycin in PBS: Prepare a 1000X solution of 100 mg/mL (working solution 100 µg/mL) kanamycin by dissolving in water. Add 10 µL of 1000X kanamycin to 10 mL of PBS. Refrigerate dilution until ready to use.

Gibson assembly master mix: Mix 320 µL isothermal reaction buffer (25% PEG-8000, 500 mM Tris-HCl, 50 mM $MgCl_2$, 50 mM DTT, 1 mM dNTPs, 5 mM NAD, pH 7.5), 0.64 µL of 10 U/µL T5 exonuclease, 20 µL of 2 U/µL Phusion DNA polymerase, 160 µL of 40 U/µL Taq DNA Ligase, and enough water to bring the total volume to 1.2 mL. Aliquot 15 µL at a time into microcentrifuge tubes and store at -20°C. Pre-made Gibson assembly master mix can be alternatively be purchased from New England Biolabs (cat. no. E2611S).

Super Script III master mix: Mix 4 volumes of 5X SSIII First Strand Buffer (supplied with SSIII), 1 volume of 0.1 M DTT, and 1 volume of 10 mM dNTPs. Store at -20°C.

Procedure for in-cell SHAPE-Seq in *E. coli*

Cloning (~1 week) – This section should be skipped unless adding a new sense/antisense RNA to platform for expression in *E. coli*.

1| Design primers suitable for Gibson Assembly of the sense/antisense insert and platform vectors.
   a. To insert an RNA sequence in the *antisense* platform, amplify the antisense vector with 5'-GCCTCTACCTGCTTCGGCCG-3' and 5'-ACTAGTATTATACCTAGGACTGAGCTAGC-3'. Design primers for the insert using 5'-GCTCAGTCCTAGGTATAATACTAGTxxxxx-3' for the forward primer and 5'-GCCGAAGCAGGTAGAGGCzzzzzz-3' for the reverse (replace x's with primer sequence specific to 5' end of antisense RNA and z's with reverse complement of 3' end, both with a T_m of about 58°C).
   b. To insert an RNA sequence in the *sense* platform, amplify the sense vector with 5'-ATGCTAGCAAAGGAGAAGAACTTTTC-3' and 5'-ACTAGTATTATACCTAGGACTGAGCTAGC-3'. Design primers for the insert using 5'-GCTCAGTCCTAGGTATAATACTAGTxxxxx -3' for the forward primer and 5'-GAAAAGTTCTTCTCCTTTGCTAGCATzzzzzz-3' for the reverse (replace x's with primer sequence specific to 5' end of sense RNA and z's with reverse complement of 3' end, both with a T_m of about 58°C). Note that the overlap for SFGFP contains the start codon - modify these overlaps if other sequences are required near the start codon.

2| Perform PCR using the Phusion PCR kit (NEB) according to the manufacturer's instructions, using the following thermal cycling conditions:

Cycle Number	Denature	Anneal	Extend	Hold
1	98°C, 30 s			
2-25	98°C, 15 s	3°C above primer's T_m	72°C, 15 s/kb	
26			72°C, 5 min	
				4°C

3| Digest PCR products with 1 µL DpnI at 37°C for 30 min.
4| Check on agarose gel to verify the size of the PCR products.
5| Purify the PCR products using the QIAquick PCR purification kit.
6| Measure the DNA concentration of the DNA fragments to be assembled with the Qubit or Nanodrop.
7| Thaw a tube of 1.33X Gibson assembly master mix on ice for each construct to assemble. Keep on ice until use.
8| Add approximately 3:1 (molar ratio) insert:platform vector to be assembled to a total volume of 5 µL, and a to 15 uL of 1.33X Gibson assembly master mix.
9| Incubate at 50°C for 1 hr
10| Place assembly products on ice to cool to least room temperature.
11| Transform assembled DNA into chemically competent *E. coli* cells (we use KCM competent NEBTurbo).
12| Incubate on ice for 20 min. Heat shock at 42°C for 1.5 min and then place back on ice for 1 min.
13| Add 50 µL 2YT medium (or other rich media such as SOB or SOC) and incubate the cells with vigorous shaking at 37°C for 1 hr. While shaking, warm agar plates with appropriate antibiotic at 37°C.

14| Plate the cells and incubate overnight at 37°C.
15| Grow and extract plasmids for sequencing, using the QIAQuick plasmid purification kit.
16| Store sequence confirmed plasmids at -20°C until ready to use.

Preparation for in-cell probing (3 days, ~3 hours of active effort) – Skip to step 19 if targeting endogenously expressed RNAs.

17| Determine which combinations of sense and antisense to test.
   a. Due to how intensive future steps are, it is advised to not do more than 10-12 total in-cell SHAPE-Seq experiments at a time. We recommend 1-4 for those new to the protocol.
      i. If characterizing more than 6 samples, it may be helpful to get a second person to help during the modification and extraction steps.

18| (Co-)Transform the sense/antisense plasmid(s) into the strain being used for testing functionality.
   a. Include empty plasmid controls to correct for autofluorescence later.
   b. We recommend co-transforming sense plasmids with a control antisense plasmid when only the sense RNA is being studied to maintain consistency between fluorescence measurements.

19| Plate transformed cells on an LB+Agar plate with the appropriate antibiotics and grow overnight at 37°C.
   a. If targeting endogenously expressed RNAs, plate cells directly with no antibiotics. Alternatively, this step could be skipped and cells could be grown directly from glycerol stocks, etc.

20| To an autoclaved 96-well culture block, add 1 mL of LB with antibiotic (if required). Pick individual colonies from the plates and inoculate the media with the corresponding antibiotic resistance(s). Grow the cells overnight on an incubator shaker at 37°C and 1,000 rpm.
   a. It is recommended to start this growth late in the day.
   b. Replace LB with other medium if desired.

21| The next day, add 24 µL of the primary inoculum to 1.2 mL fresh, pre-warmed LB with antibiotic(s) (if required). Allow to grow for at least 3 hours at 37°C and 1,000 rpm on the shaker.
   a. The time for growth can be adjusted based on the desired functional testing assay and speed of growth of the cells.
   b. An OD of 0.3 or higher is recommended for probing, but not necessarily required.
   c. Replace LB with other medium if desired.
   d. Alternative dilution factors and growing times can be used instead so long as a suitable OD is reached.

RNA Modification & Fluorescence Assay (10-25 min)

22| During the 3 hour (or greater) subculture, prepare 15 µL of 250 mM 1M7 in neat DMSO for each in-cell SHAPE-Seq experiment being performed. Also preheat 200 µL of TRIzol Max Bacterial Enhancement reagent at 95°C for each in-cell SHAPE-Seq experiment.

23| After the 3 hour (or greater) incubation is complete, take 150 µL of each culture and put into labeled 1.5 mL microcentrifuge tubes. Spin these tubes at ≥ 15,000 x g for 1 min and discard the used media. Set aside until step 26.

24| Add 13.3 µL of 250 mM 1M7 to clean, empty adjacent wells for each culture. Also add 13.3 uL of anhydrous DMSO to a second empty well for each culture. The modification experiment will be performed in these wells. Using a multichannel pipet, quickly add 500 µL of culture to the wells containing either 1M7 (positive/(+) channel) or DMSO (negative/(-) channel).

25| Return the 96-well culture block to the shaker for 3 min.

26| During the 3 min incubation, resuspend the cultures from Step 23 in 200 µL cold PBS with 1X kanamycin. Store these at 4°C until Step 31.

27| Remove the block from the shaker and pipet the (+) and (-) samples into individual, labeled 1.5 mL microcentrifuge tubes.

28| Spin the tubes at ≥ 15,000 x g for 1 min and aspirate the used media. Resuspend each pellet in 100 µL of the preheated TRIzol Max Bacterial Enhancement reagent.

29| Heat the samples in the TRIzol Max Bacterial Enhancement reagent at 95°C for 4 min.

30| Remove from heat and add 500 µL TRIzol reagent to each. Shake vigorously for ≥ 5 s to mix.

   a. Be careful that tubes are well sealed when shaking.

CAUTION TRIzol contains phenol, which causes burns, lung edema, and kidney damage.

CAUTION TRIzol contains chloroform, a known carcinogen, and must be handled with care.

(Pause Point)

31| At this time, pipet the cells in PBS (from step 26) into a 96-well plate with a clear bottom. Include a well containing 200 µL of PBS with 1X kanamycin as a blank.

   a. Skip Steps 31-32 if not measuring a fluorescent output.

32| Measure OD_{600} and fluorescence (485 nm excitation, 520 nm emission – this may be adjusted if you are using a different reporter system). Calculate the fold activation/repression by subtracting the PBS blank from the OD and fluorescence measurements for all cells. Normalize all of the wells for growth rate by dividing fluorescence intensity (FL) by OD. Then subtract cell autofluorescence from all wells using a non-fluorescent cell control well's FL/OD. The resulting FL/OD values can be used to compare ON and OFF levels between different conditions.

RNA Extraction (~1.5 hours)

33| Incubate the TRIzol-containing tubes for ≥ 5 min at room temperature.

34| Add 100 µL chloroform to each tube, shake vigorously for 10 s, and incubate at room temperature for another 2 min.

   a. Be careful that tubes are well sealed when shaking.

35| Centrifuge the TRIzol-containing tubes at 12,000 x g and 4°C for 15 min.

36| Carefully transfer the clear aqueous phases (up to 350 µL) to new 0.7 mL microcentrifuge tubes.

37| Add 250 µL cold 100% isopropyl alcohol and mix by inverting the tubes 6-8 times. Incubate at room temperature for 10 min.

   a. Add 1 µL of 20 mg/mL glycogen if RNA yield is expected to be low.

38| Spin the tubes at ≥ 15,000 x g and 4°C for 10 min. Aspirate the alcohol and wash with 500 µL 70% EtOH. Respin the tubes for 2 min and aspirate the EtOH. Respin again for 2 min and aspirate the remaining ethanol, allowing traces of remaining EtOH to air dry.

39| Resuspend each resulting RNA sample (will be 2 for each in-cell SHAPE-Seq experiment) in 10 µL RNase-free water.

   a. **(Pause Point)** The RNAs can be stored at -20°C overnight.

Reverse Transcription (1-1.5 hours)

40| Prepare a thermal cycler and start the following protocol (RT) to preheat the block:

Step Number	Step name	Temperature	Time
1	Hot start	95°C	∞
2	Denature	95°C	2 min
3	Denature	65°C	5 min
4	Cooling	45°C	∞
5	Pre-heat	45°C	1 min
6	Extend	52°C	25 min
7	Inactivate	65°C	5 min
8	Infinite hold	4°C	∞

41| Add 3 µL of 0.5 µM reverse transcription primer. If using the two-plasmid system, use primer A for antisense RNA only, primer B for sense RNA only, or a mixture of both primers A and B for sense and antisense mixtures. If targeting endogenously expressed RNAs, use primers designed specifically for those RNAs, such as primers C-E.
   a. For rare or weakly expressed RNAs, reduce the primer concentration to 50 nM.
   b. The primers in Supplementary Table S3 are for the RNAs described in this work, other sequences can also be used.
   c. Be mindful of plasmid copy numbers when mixing RT primers, in the work presented we used a 50-50 mixture. However, balancing the relative amount of RT primer for different target abundances is recommended for future work.

42| Start the RT protocol in Step 40.

43| When the thermal cycler block is preheated to 95°C, place each tube on the thermal cycler, and advance the steps.

44| During the first 7 min of incubation (2x Denature), mix 6 µL of SSIII Master Mix and 1 µL of 0.5X SSIII reverse transcriptase (diluted in its storage buffer) for each RNA sample in a microcentrifuge tube.
   a. We recommend mixing some extra SSIII Master Mix and enzyme to avoid running short during Step 45.

45| At the end of the first 65°C step (2nd Denature), move all the tubes to ice for 30 s. Then add 7 µL of the SSIII Master Mix/reverse transcriptase mix (from Step 44) to each tube, mix well, and return to ice. Once all of the RNAs have received the 7 µL, return all the tubes to the thermal cycler and continue the thermal cycler procedure.
   a. IMPORTANT - When adding the Master Mix/reverse transcriptase mix, make sure to knock down the condensed water from the sides of the tubes to maintain the reaction volume.

46| Upon completion of the RT protocol, remove all the tubes to ice and add 1 µL of 10 M NaOH.

47| Incubate the tubes at 95°C for 5 min to hydrolyze the RNA.

48| Partially neutralize the solutions by adding 5 µL of 1 M HCl.

49| Ethanol precipitate by adding 78 µL of ice-cold 100% ethanol to each tube, inverting 6-8 times, and incubating at -80°C for 15 min.
   a. **(Pause Point)** The cDNA can be stored at -80°C.

50| Centrifuge the tubes at ≥ 15,000 x g and 4°C for 15 min. Aspirate the ethanol and wash with 500 µL of 70% ethanol. Respin for 2 min, aspirate, respin for 2 min again, and aspirate the remaining ethanol. Allow to air dry.

51| Resuspend each cDNA in 22.5 µL of RNase-free water.

a. **(Pause Point)** The cDNA can be stored at -20°C.

A_adapter_B Ligation & Purification (~4 hours)

52| Add 3 µL of 10x CircLigase reaction buffer, 1.5 µL 50 mM $MnCl_2$, 1.5 µL 1 mM ATP, 1 µL CircLigase, and 0.5 µL of 100 µM oligonucleotide F to each tube with cDNA.

a. For samples with reduced RT primer concentrations (from Step 41), reduce oligonucleotide F concentration to 10 µM.

53| Incubate at 60°C for 2 hrs, followed by a 10 min incubation at 80°C to inactivate CircLigase I.

a. Meanwhile, thaw a tube of 20 mg/mL glycogen.

54| After the ligation is complete, add 70 µL RNase-free water, 10 µL 3.0 M sodium acetate pH 5.5, 1 µL 20 mg/mL glycogen (for visualization), and 300 µL of ice-cold 100% ethanol. Mix.

a. **(Pause Point)** The cDNA can be stored at -80°C.

55| Incubate at -80°C for 30 min then spin at ≥ 15,000 x g at 4°C for 30 min. Aspirate the ethanol, re-spin for 2 min, and aspirate any remaining ethanol. Resuspend each cDNA in 20 µL water.

56| Resuspend a bottle of Agencout AMPure XP beads. Add 36 µL of bead slurry to each tube containing cDNA and mix well by pipetting up and down.

57| Continue purification of ssDNA libraries according to manufacturer's protocol, eluting in 20 µL TE buffer.

a. We recommend using the 96-well plate method for a large number of samples.
b. **(Pause Point)** The eluted cDNA can be stored at -20°C.

Quality Control (QC) (1 hour + CE time) – An alternative method is mentioned in Step 76

58| Mix the following PCR or each sample: 1.5 µL ssDNA library, 1 µL of 1 µM primer G (for (+) samples) or primer H (for (-) samples), 1 µL of 0.1 µM selection primer [ex.: primer J (antisense samples), L (sense samples), or N, P, or R (endogenous)], 1.5 µL of 1 µM primer I, 5 µL of 5X Phusion reaction buffer (NEB), 0.5 µL of 10 mM dNTPs, 0.25 µL (0.5 U) Phusion Polymerase, and H_2O to 25 µL.

a. The selection primer chosen should match the RT primer sequence and contain a further 3' extension.
b. For rare transcripts, exclude primer I in the initial reaction mix (see Step 59b).
c. If using priming sites other than those designed in Supplementary Table S3, you will need to design an alternative set of mismatch primers instead of primers J-S. To do this, add the sequence CTTTCCCTACACGACGCTCTTCCGATCTYYYR to the 5' end of the RT primer for (-) samples, and CTTTCCCTACACGACGCTCTTCCGATCTRRRY for (+) samples. Then, extend the 3' end of primer a few nucleotides into the cDNA sequence such that it mismatches the 5' end of oligonucleotide F. Protect this mismatch from 3' -> 5' exonuclease activity with phosphorothioate modifications.
d. Either positive or negative primer can be used as they are the same length and will appear the same on the electropherogram output.
e. For cDNAs containing more than 1 RT primer, mix a separate QC library for each RT primer. For example, if using both sense and antisense, mix 2 QC reactions, one with primer J and

one with primer L. If using more than 3-4 RT primers, you may want to combine some of the selection primers together when doing QC and look for multiple full length peaks instead.

59| Run the LIB2CE15 protocol on a thermal cycler:

Cycle Number	Denature	Anneal	Extend	Hold
1	98°C, 30 s			
2-16	98°C, 15 s	63°C, 30 s	72°C, 30 s	
17			72°C, 5 min	
18				4°C

a. When analyzing low abundance transcripts (not those used in the platforms from Supplementary Figure S1), the number of cycles can be increased. However, non-specific amplification will start to occur at significant levels after 15 cycles of amplification when primer I is included in libraries derived from low abundance transcripts.
b. For rare transcripts, perform 15 cycles of amplification without primer I (more cycles can be done if necessary). Then, add the 1.5 µL of 1 µM primer I directly to the reaction and perform another 15 cycles of amplification to finish building the libraries.

60| Add 50 µL H_2O to the (+) reaction, then mix the (+) and (-) reaction products together.

61| Ethanol precipitate each (+/-) reaction mixture by adding 10 µL 3.0 M NaOAc pH 5.5 and 300 µL of ice-cold EtOH to each combined reaction pair.

62| Incubate the mixtures at -80°C for 15 min.

63| Centrifuge each at ≥ 15,000 x g and 4°C for 15 min.

64| Aspirate the EtOH, re-spin for 2 min, and aspirate the remaining EtOH. Air dry the pellet.

65| Dissolve each pellet in 10 µL of deionized formamide and add 0.2-0.3 µL of GeneScan 500 LIZ standard.

a. We recommend heating at 85-95°C for 5-10 min to aid dissolving.

66| Run each sample on a capillary electrophoresis (CE) machine.

67| Use the LIZ standard to identify peak lengths (We recommend SHAPEFinder (19) for easy viewing). There should be a full-length peak clearly visible at the length of the cDNA expected + RT primer length + 96 bp for the PCR overhangs for quality analysis. Peaks at RT primer length + 96 bp are indicative of RT primer-A_adapter_B dimers. A good library trace should show considerable peak height and a good full length:RT-A_adapter_B dimer ratio, with minor peaks in between for RT stops.

a. The antisense libraries have a dimer (no cDNA) length of 117 bp, while the sense libraries have a dimer length of 122 bp. The fully extended peak (to the 5' end of the RNA) should be expected at 96 nt + length of the RNA extended, including the RT primer length.
b. Shown below is a good QC trace for the antisense taR12 from the riboregulator system (96 nt long). Note that the dimer side product peak is low, but the full-length cDNA peak is high. A large concentration of unextended primer is normal, as the PCR described above does not consume all the fluorescent primer.

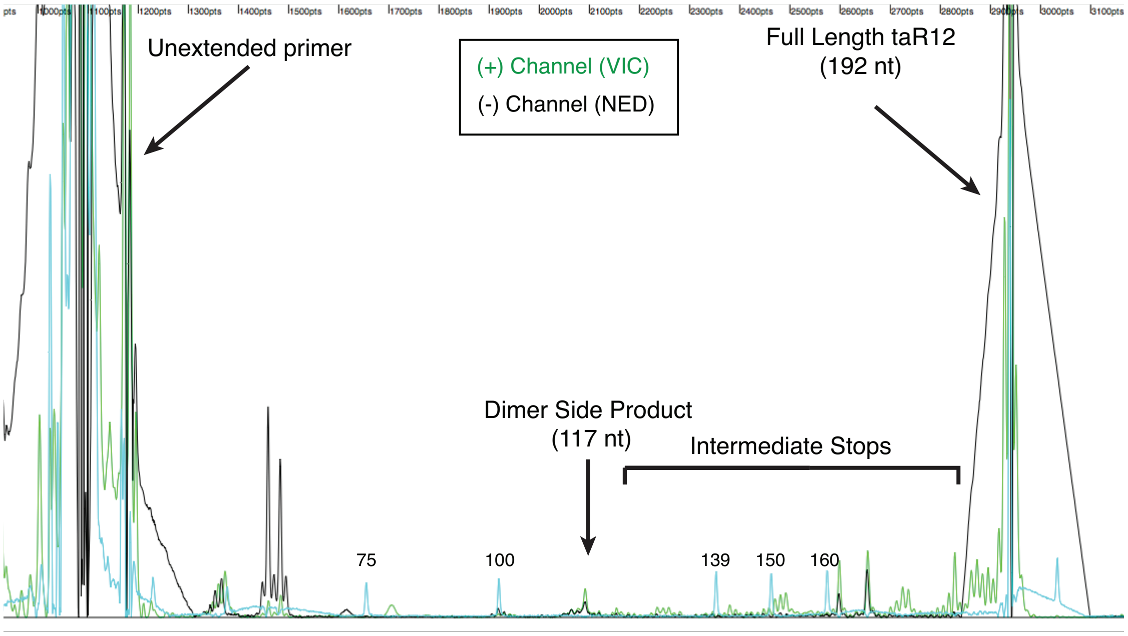

dsDNA Library Construction – (~1-2 hours)

68| Mix the following PCR for each sample: 3 µL ssDNA library, 2 µL of 0.1 µM primer J (+) or K (-) (antisense samples) or primer L (+) or M (-) (sense samples), 0.25 µL of 100 µM primer I, 0.25 µL of 100 µM Illumina indexing primer T-AQ (choose which one depending on indexes being used), 10 µL of 5X Phusion reaction buffer (NEB), 0.5 µL of 10 mM dNTPs, 0.5 µL (1 U) Phusion Polymerase, and H_2O to 50 µL.

a. As discussed in Step 58b, use the appropriate mismatch selection primers if not using RT primers A or B.
b. The relative amounts of primers or ssDNA library can be increased to increase yields if desired or initial yields are low.
c. If characterizing low abundance or rare transcripts and the split PCR method from Steps 58-59 was used, similarly exclude primer I from the first round of amplification, then add for the second as done in those steps (at the concentration listed in this step).

69| Run the SEQPHU15 protocol on a thermal cycler:

Cycle Number	Denature	Anneal	Extend	Hold
1	98°C, 30 s			
2-16	98°C, 15 s	65°C, 30 s	72°C, 30 s	
17			72°C, 5 min	
18				4°C

a. If a different number of cycles, other than 15, were used in step 59, use that number of cycles here as well. Also see Note 68c.

70| Chill the reactions at 4°C for 5 min.

71| Add 0.25 µL (5 U) of ExoI to each library reaction.

72| Incubate at 37°C for 30 min.

73| Resuspend a bottle of Agencout AMPure XP beads. Add 90 µL of bead slurry to each tube containing cDNA and mix well by pipetting up and down.

74| Continue purification of dsDNA libraries according to manufacturer's protocol, eluting in 20 μL TE buffer. We recommend using a 96-well plate method for a large number of samples.

75| Measure the concentration of each dsDNA library using Qubit or another preferred method for quantifying concentration of DNA.

   a. **(Pause Point)** The eluted libraries can be stored at -20°C.

76| *(Optional) Alternative Quality Control Method:* Run 1 μL of the dsDNA libraries on a BioAnalyzer high sensitivity dsDNA chip. Look for the same features described in Step 67, except that the cDNA full length should appear at 125 bp + RT primer length + the cDNA length (instead of cDNA expected + RT primer length + 96 bp).

   a. The dimer (no cDNA) length of dsDNA for the antisense is 146 bp, while the sense is 151 bp for the platform used in this work.

Next-Generation Sequencing – (1 day – 2 weeks, depending on resources available)

77| Use the concentration measurements from each library and the quality control traces to estimate the molarity of each library.

78| Mix a few microliters of each library together to pool all of the SHAPE-Seq libraries such that they are all balanced by mole.

79| Run on the Illumina platform, using 2x35 bp paired end reads. We recommend the MiSeq v3 kit for less than 30-40 libraries that are properly balanced between all indexes and RT primers.

   a. Longer read lengths can be used, but are generally unnecessary. The read length only needs to be long enough to uniquely align the 3' ends of all RNAs analyzed within a TruSeq index.
   b. Do not process the data using the native Illumina adapter trimming; it will be removed by downstream in the data processing steps.

Data Processing (~0.5-3 hours, if software is previously installed) – Requires Unix, Linux, or Mac OS X platform

80| If not previously installed, download and install Spats and its associated software from spats.sourceforge.net, using the instructions provided there.

   a. Other software required for running Spats includes: bowtie (http://bowtie-bio.sourceforge.net), the fastx toolkit and libgtextutils (http://hannonlab.cshl.edu/fastx_toolkit/download.html), boost (www.boost.org), and Python.
   b. Note: Future releases of Spats can be found at: http://spats.sourceforge.net/

81| Obtain the sequencing data from a local store or BaseSpace and unzip the files to get the .fastq files

   a. Future releases of Spats may contain library analysis tools.

82| Create a fasta (.fa) style formatted targets file as indicated in the Spats documentation. For each .fastq pair (Read 1 and Read 2), there should only be one .fa file, that contains all of the target RNAs.

   a. For each RNA, create a new line beginning with a caret '>' followed by the RNA name. The line beneath should then contain your sequence of interest from the 5' end to the 3' end of the cDNA product.

83| Run adapter_trimmer.py. By default, the script will determine a number of the parameters for Spats automatically by analyzing the targets input. However, some values must be set if differing from the default. adapter_trimmer.py will find and remove sequences containing Illumina sequencing adapters.

a. If using something other than 2x35 bp sequencing, be sure to include the flag '--read-len XX' where XX is the read length from the 2xXX bp paired end sequencing. adapter_trimmer.py assumes that both read lengths are equal.

84| Run Spats with the .fastq output from adapter_trimmer.py, where <rna.fasta> is the targets file, RRRY and YYYR are the treated and untreated handles (respectively). If adapter_trimmer.py was run, the adapter trimming capabilities in Spats itself are not necessary to use. The output directory will contain a text file named reactivities.out, which are the results.

a. We recommend always using adapter_trimmer.py, not the trimming algorithm in Spats itself.

85| Normalize the output θ_i values to ρ_i values according to Supplementary Equations 1-4. These ρ_i values can be plotted to obtain reactivity maps or used as secondary structure prediction constraints.

RNA Structure Prediction (20 minutes)

86| Download and install RNAStructure (http://rna.urmc.rochester.edu/RNAstructure.html) or use the webserver (http://rna.urmc.rochester.edu/RNAstructureWeb/Servers/Predict1/Predict1.html)

87| Create a .shape text file containing the ρ_i values for each position. To do this, create a tab separated text file where the first column is 1→L, where L is the length of the RNA, and the second column is the ρ_i value for that position (5' -> 3'). For nucleotides to be analyzed that do not have ρ_i values, enter '-999' instead.

a. Note: The length of the RNA analyzed will need to match the number of positions there is reactivity information for in the .shape file.

88| If using the webserver, copy and paste the RNA sequence to be analyzed in all caps into the 'Sequence' box, adjust any parameters as desired, then choose the .shape file to upload under 'Select SHAPE Constraints File:" Adjust the SHAPE Intercept (b) to be -0.3 and the SHAPE slope (m) to be 1.1 and submit the query. The minimum free energy structure appears after calculations complete.

a. Make sure the sequence is in all caps, lower case is forced to be single-stranded.

89| If using the GUI version, create a new .seq file using the RNA sequence of interest in all caps. Then choose "RNA..Fold RNA Single Strand" and select the sequence file for the RNA of interest. Then select Force..Read SHAPE Reactivity – Pseudo-Energy Constraints" and choose the .shape file containing the appropriate ρ_i values. Adjust the SHAPE Intercept to be -0.3 and the SHAPE slope to be 1.1 and hit 'OK'. Run the calculations by hitting 'Start'. The minimum free energy structure will appear after calculations are complete.

a. Make sure the sequence is in all caps, lower case is forced to be single-stranded.
b. The command line version also accepts SHAPE reactivity constraints. See http://rna.urmc.rochester.edu/Text/Fold.html for instructions.

References.

1. Pédelacq, J.-D., Cabantous, S., Tran, T., Terwilliger, T.C. and Waldo, G.S. (2006) Engineering and characterization of a superfolder green fluorescent protein. *Nat Biotechnol*, **24**, 79–88.
2. Chen, Y.-J., Liu, P., Nielsen, A.A.K., Brophy, J.A.N., Clancy, K., Peterson, T. and Voigt, C.A. (2013) Characterization of 582 natural and synthetic terminators and quantification of their design constraints. *Nat. Meth.*, **10**, 659–664.
3. Aviran, S., Trapnell, C., Lucks, J.B., Mortimer, S.A., Luo, S., Schroth, G.P., Doudna, J.A., Arkin, A.P. and Pachter, L. (2011) Modeling and automation of sequencing-based characterization of RNA structure. *Proc. Natl. Acad. Sci. U.S.A.*, **108**, 11069–11074.
4. Aviran, S., Lucks, J.B. and Pachter, L. (2011) RNA structure characterization from chemical mapping experiments. In *Proc. 49th Allerton Conf. on Communication, Control, and Computing*. 1743-1750, Monticello, IL. doi: 10.1109/Allerton.2011.6120379
5. Isaacs, F.J., Dwyer, D.J., Ding, C., Pervouchine, D.D., Cantor, C.R. and Collins, J.J. (2004) Engineered riboregulators enable post-transcriptional control of gene expression. *Nat. Biotechnol.*, **22**, 841–847.
6. Reuter, J.S. and Mathews, D.H. (2010) RNAstructure: software for RNA secondary structure prediction and analysis. *BMC Bioinformatics*, **11**, 129.
7. Wells, S.E., Hughes, J.M., Igel, A.H. and Ares, M. (2000) Use of dimethyl sulfate to probe RNA structure in vivo. *Meth. Enzymol.*, **318**, 479–493.
8. Steitz, J.A. and Jakes, K. (1975) How ribosomes select initiator regions in mRNA: base pair formation between the 3' terminus of 16S rRNA and the mRNA during initiation of protein synthesis in Escherichia coli. *Proc. Natl. Acad. Sci. U.S.A.*, **72**, 4734–4738.
9. Mutalik, V.K., Qi, L., Guimaraes, J.C., Lucks, J.B. and Arkin, A.P. (2012) Rationally designed families of orthogonal RNA regulators of translation. *Nat. Chem. Bio.*, **8**, 447–454.
10. Case, C.C., Simons, E.L. and Simons, R.W. (1990) The IS10 transposase mRNA is destabilized during antisense RNA control. *EMBO J.*, **9**, 1259–1266.
11. Loughrey, D., Watters, K.E., Settle, A.H. and Lucks, J.B. (2014) SHAPE-Seq 2.0: systematic optimization and extension of high-throughput chemical probing of RNA secondary structure with next generation sequencing. *NAR*, **42**, e165. doi:10.1093/nar/gku909
12. Cordero, P., Lucks, J.B. and Das, R. (2012) An RNA Mapping DataBase for curating RNA structure mapping experiments. *Bioinformatics*, **28**, 3006–3008.
13. Yusupov, M.M., Yusupova, G.Z., Baucom, A., Lieberman, K., Earnest, T.N., Cate, J.H. and Noller, H.F. (2001) Crystal structure of the ribosome at 5.5 A resolution. *Science*, **292**, 883–896.
14. Villa, E., Sengupta, J., Trabuco, L.G., LeBarron, J., Baxter, W.T., Shaikh, T.R., Grassucci, R.A., Nissen, P., Ehrenberg, M., Schulten, K., et al. (2009) Ribosome-induced changes in elongation factor Tu conformation control GTP hydrolysis. *Proc. Natl. Acad. Sci. U.S.A.*, **106**, 1063–1068.
15. Mortimer, S.A., Trapnell, C., Aviran, S., Pachter, L. and Lucks, J.B. (2012) SHAPE-Seq: High-Throughput RNA Structure Analysis. *Curr. Protoc. Chem. Biol.*, **4**, 275–297.
16. Mathews, D.H., Disney, M.D., Childs, J.L., Schroeder, S.J., Zuker, M. and Turner, D.H. (2004) Incorporating chemical modification constraints into a dynamic programming algorithm for prediction of RNA secondary structure. *Proc. Natl. Acad. Sci. U.S.A.*, **101**, 7287–7292.
17. Gibson, D.G., Young, L., Chuang, R.-Y., Venter, J.C., Hutchison, C.A. and Smith, H.O. (2009) Enzymatic assembly of DNA molecules up to several hundred kilobases. *Nat Meth*, **6**, 343–345.
18. Turner, R., Shefer, K. and Ares, M. (2013) Safer one-pot synthesis of the 'SHAPE' reagent 1-methyl-7-nitroisatoic anhydride (1m7). *RNA*, **19**, 1857–1863.
19. Vasa, S.M., Guex, N., Wilkinson, K.A., Weeks, K.M. and Giddings, M.C. (2008) ShapeFinder: a software system for high-throughput quantitative analysis of nucleic acid reactivity information resolved by capillary electrophoresis. *RNA*, **14**, 1979–1990.
